# Supplementary material for: Randomized clinical trial to assess the protective efficacy of a Plasmodium vivax CS synthetic vaccine
Source: Nat Commun. 2022 Mar 25;13:1603. doi: 10.1038/s41467-022-29226-3 (PMC8956637; doi:10.1038/s41467-022-29226-3)
Supplement: Supplementary file 1 — Supplementary Information [file 41467_2022_29226_MOESM1_ESM.pdf]

Evaluation of the protective efficacy of a synthetic vaccine derived from the *Plasmodium vivax* CS protein

Supplement Note 1

Protocol code: 2304-493-26202

Sponsor
Departamento Administrativo de Ciencia, Tecnología e Innovación, Colciencias.

Principal investigator: Sócrates Herrera, MD

Versión: 6.0
13 February 2015

STATEMENT OF COMPLIANCE

The study will be carried out in compliance with Good Clinical and Laboratory Practices (GCP and GLP) as required by the ISO 9000, 2001 guidelines. This study will be approved by the Institutional Review Board (IRB) (Comité de Ética Institucional del Centro Internacional de Vacunas (CECIV). This protocol contains informed consent (Supplement Note 1), which includes information about the volunteers' guarantees of participating in the study.

Volunteers’ recruitment and study activities will begin after approval of the protocol by the local IRBs. All aspects of the protocol involving human subject participation will be carried out under the NIAID clinical terms of awards and the ICH/GCP guidelines.

SIGNATURE PAGE

The signatures below constitute acknowledgment of the protocol and the attachments and provide the necessary assurances that this clinical study will be conducted according to all stipulations of the protocol, including all confidentiality statements and according to local legal and regulatory requirements and to the principles outlined in applicable ICH guidelines.

Principal Investigator – *Name of Site*:

Signed: ______________________________ Date: 17-02-2014

Socrates Herrera MD

TABLE OF CONTENT

STATEMENT OF COMPLIANCE 2
SIGNATURE PAGE 3
TABLE OF CONTENT 4
GLOSSARY OF ABBREVIATIONS 6
1. ROLES 8
2. 10
3. INTRODUC24
3.1 Error! Bookmark not defined.
3.2 Error! Bookmark not defined.4
3.3 LimitaError! Bookmark not defined.5
3.4 Error! Bookmark not defined.
3.4.1 Development of *P. vivax* malaria natural immunity 16
3.4.2 Identification and characterization of the CS protein 16
3.4.3 Development of the *P. vivax* CS protein as a malaria vaccine candidate 17
3.4.4 Preliminary preclinical studies in Colombia and other countries. 17
3.4.5 *P. vivax* CS protein phase I clinical trials 18
3.4.6 Infectious challenge models for *P. falciparum* and *P. vivax* 19
3.4.7 Standardization of a challenge model with *P. vivax* sporozoites 19
3.5 Scientific Justification 20
4. Error! Bookmark not defined.0
5. OBJEError! Bookmark not defined.1
5.1 General objective 21
5.2 Specific objectives Error! Bookmark not defined.1
6. Error! Bookmark not defined.2
6.1 Steps 1 and 3. Volunteers immunization and infectious challenge Error! Bookmark not defined.2
6.2 Step 2: Donation of infected blood Error! Bookmark not defined.4
7. 255
7.1 Step 1: Volunteers selection and immunization 25
7.2 Step 2: Donation of infected blood 29
7.3 Step 3. Sporozoite obtention and infectious challenge 32
8. LABORAT390
8.1 Malaria diagnosis: 40
8.2 Infectious diseases screening test. 40
8.2.1 Results interpretation 394
9. Error! Bookmark not defined.46
9.1 Records keeping 46
10. RISKS FOR THE VOLUNTEERS, RESEARCH GROUP AND THE ENVIRONMENT; PRECAUTIONS TO MINIMIZE RISKS 47
10.1 Risks for blood donor volunteers 47
10.2 Risks to volunteers associated with CHMI 48
10.3 Risks to volunteers associated with antimalarial treatmen. 49
10.4 Risks for those conducting the study 50
10.5 Risks and precautions associated with the environment 51
11. BENEFI491
11. 1 Benefits for blood donor volunteers. 51

11.2 Benefits for volunteers enrolled in the CHMI 51
12. COMPENSATION 51
12.1 Compensation for blood donors. 51
12.2 Compensation for volunteers enrolled in the malaria challenge. 52
13. CRITERIA FOR DROPPING/WITHDRAWAL OF VOLUNTEERS. 52
14. ADVERSE EVENTS. 52
14.1 Serious adverse event: 57
14.2 Classification of AEs- Association with study activities. 57
14.3 AEs report. 58
14.4 AEs follow-up period 59
15. ETHICAL CONSIDERATIONS. 59
15.1 Approval of the ethics committees and organization plan. 59
15.2 Ethics committees affiliation to the United States FWA. 60
15.3 Research-related injuries. 60
16. CIV AND ASOCLINIC GOOD CLINICAL PRACTICES (GCP) AND GOOD LABORATORY PRACTICES (GLP). 61
17. CONFIDENTIALITY 61
18. RULES FOR STUDY INTERRUPTION. 62
19. USE OF THE INFORMATION AND PUBLICATIONS ARISING FROM THE STUDY 62
20. DEVIATIONS AND MODIFICATIONS TO THE PROTOCOL. 62
21. WITHDRAWAL OF VOLUNTEERS FROM THE STUDY. 62
21.1 Follow-up of volunteers who do not continue in the study. 63
22. TIME TABLE 64
23. REFERENCES 65

GLOSSARY OF ABBREVIATIONS

ABBREVIATIONS	DEFINITIONS
A.	*Anopheles*
AE	Adverse Event
MFA	Artificial Membrane Feeding Assay
Anti-HBc	Hepatitis B core antibodies
BUN	Blood urea nitrogen
β-HCG	Human chorionic gonadotropin-beta subunit
CAB	Center for Applied Biotechnology
CIV/MVDC	Centro Internacional de Vacunas (Malaria Vaccine Development Center)
CHMI	Controlled Human Malaria Infection
CRF	Case Report Form
GCP	Good Clinical Practices
GLP	Good Laboratory Practices
DNA	Deoxyribonucleic acid
ECG	Electrocardiogram
ELISA	Enzyme-Linked ImmunoSorbent Assay
EPS/HPC	Empresa Promotora de Salud (Health Promoting Company)
FDA	Food and Drug Administration
FTA-ABS	Fluorescent-Treponemal Antibody Absorbed.
FWA	Federal Wide Assurance
G6PD	Glucose 6 Phosphate Dehydrogenase
HBsAg	Hepatitis B Surface Antigen
HBV	Hepatitis B Virus
HCV	Hepatitis C Virus
HIV	Human Immunodeficiency Virus
HTLV	Human T-lymphotropic virus
IC	Informed Consent
INS	Instituto Nacional de Salud (Colombia) - National Institute of Health
IFAT	Immunofluorescence Antibody Test

IPS	Institución Prestadora de Servicios de Salud - Health Services Provider Institution
IRB/EC	Institutional Review Board - Ethics Committee
LDH	Lactate Dehydrogenase
NIH	National Institute of Health (US)
NIAID	National Institute of Allergy and Infectious Diseases (US
P.	*Plasmodium*
*Pv*CS	*Plasmodium vivax* circumsporozoite protein
SAE	Serious Adverse Event
SOP	Standard Operating Procedure
spp.	Species
SP	Sulfadoxine Pyrimethamine
TBS	Thick Blood Smear
VES	Erythrosedimentation rate
WHO	World Health Organization

1. ROLES

Principal Investigator:

Sócrates Herrera, MD.
Managing Director,
Centro Internacional de Vacunas (CIV)
Km 6 Vía Cali – Puerto Tejada. Corregimiento El Hormiguero
Telephone: (57) 317-517 0552, 2-5216228
sherrera@inmuno.org

Co-Investigators:

Myriam Arévalo-Herrera, PhD.
Scientific Director,
Centro Internacional de Vacunas (CIV)
Km 6 Vía Cali – Puerto Tejada. Corregimiento El Hormiguero
Telephone: (57) 317-517 0557, 2-5216228
marevalo@inmuno.org

Nora Céspedes, PhD
Centro Internacional de Vacunas (CIV)
Km 6 Vía Cali – Puerto Tejada. Corregimiento El Hormiguero
Telephone: (57) 2-5216228
ncespedes@inmuno.org

Giampietro Corradin, PhD.
Biochemistry Department, Lausanne University
155 Chemin des Boveresses
Epalinges, 1066
Switzerland
Giampietro.Corradin@unil.ch

José Millán Oñate, MD. Infectologist
Imbanaco Medical Center
Carrera 38 A No. 5 A 100
Telephone: 57- 2 682 1000
Cali, Valle del Cauca
Colombia
millanonate@gmail.com

Entomology Unit:

Andrés Benito Amado, BSc.
Centro Internacional de Vacunas (CIV)
Km 6 Vía Cali – Puerto Tejada. Corregimiento El Hormiguero

Telephone: (57) 2-5216228
amado@inmuno.org

Clinical Monitor: Ricardo Palacios, MD, PhD Meridional R&D
Rua Fernão Dias, 128 / 34A São
Paulo, SP, Brazil
CEP 05427-000
Telephone: +55(11)939 40670
rpalacios@meridionalrd.com

Ethics Committee: Ethics Committee, CIV (CECIV)
IRB# IRB00007039 - IRB00007040 FWA:
FWA00016072
Telephone: (57)-(2)-518-5677 Fax: (57)-(2)--554284

Ethics Committee, Imbanaco Medical Center (CMI) Carrera
38 A No. 5 A 100. Cali, Colombia.
Telephone: (57)-(2)- 6821000
Fax (57)-(2)-5186000

Data Management: Álvaro Andrés Álvarez
Centro Internacional de Vacunas (CIV)
Km 6 Vía Cali – Puerto Tejada. Corregimiento El
Hormiguero
Telephone: 2-5216228 aalvarez@inmuno.org

Estimated Number of Admitted Volunteers: Step (2) Donation of infected blood: Minimum 5, maximum 15
Step (1 y 3) Immunizations and CHMI: 32 (plus 4-6 alternatives)

Clinical Laboratory: ASOCLINIC Inmunología Ltda.
Cra 37 2 Bis 5E-08 Templete Cali, Colombia
Telephone: (57)-(2) - 5574929

2. PROTOCOL SUMMARY

**Title**	Evaluation of the protective efficacy of a synthetic vaccine derived from the *Plasmodium vivax* CS protein
**Name of the Product**	*Pv*CSP
**Study Objectives**	**General Objective**: To determine the protective efficacy induced by the *Pv*CSP vaccine formulated in Montanide ISA-51 adjuvant, in malaria-naïve and previously exposed volunteers.<br><br>**Specific Objectives:**<br><br>1) To onfirm the safety of the vaccine in naïve volunteers immunized with the *Pv*CS.<br><br>2) To determine the immunogenicity of *Pv*CS in individuals previously exposed to malaria.<br><br>3) To determine the protective efficacy of the vaccine against the infectious challenge with viable *P. vivax* sporozoites in the previous groups.<br><br>4) To evaluate the infective capacity of gametocytes in the early stages of the blood cycle of *P. vivax* in *Anopheles albimanus* mosquitoes.<br>.
**Study design**	Phase II a/b randomized, double-blind, controlled clinical trial, comparing two groups of naïve volunteers and volunteers previously exposed to malaria.
**Schedule**	Three intramuscular injections on days 0, 60, and 180, followed by an infectious challenge (CHMI) on day 210
**No. volunteers**	32 Volunteers
**Population**	Healthy adults of both genders, 16 naïve to malaria, 16 pre-exposed to infection

**Study Methods**	**Step 1: Volunteers selection and immunization:** 32 subjects who meet the inclusion criteria will be selected, then they will be immunized by IM route at months 0, 2, and 6, with peptides derived from the *P. vivax* Circumsporozoite Protein (*Pv*CS) (150 µg) formulated in Montanide ISA- 51. Repeated blood sampling will be done to assess safety using kidney, liver, and hematologic function tests. Adverse events (AE) will be reported and quantified immediately after each injection and at any time between the immunization and a 7-day follow-up period for each injection.<br>The vaccine immunogenicity will be evaluated by the production of antibodies to the parasite and to the peptides used in immunization, and by the induction of specific cytokines (IFN-γ, TNF-α, IL-2, IL-4, IL-6, and IL-10). Also, the populations of monocytes (MO), T lymphocytes (LT), and B lymphocytes (LB) in the study subjects will be characterized. Additionally, the antibodies' *in vitro* functionality will be evaluated through sporozoite-invasion inhibition assays to hepatocytes.<br><br>**Step 2: Donation of infected blood:** A group of parasite donor patients (n = 5-15) will be selected among *P. vivax* infected people who attend diagnostic centers located in endemic regions or Cali, with detected parasitemias ≥0.1%. They will be asked to donate a 35 mL blood sample that will be subjected to laboratory tests to rule out co-infections and will be used for the feeding and infection of *Anopheles* mosquitoes.<br><br>**<u>Step 3. Sporozoite obtention and infectious challenge</u>:** To evaluate the immunogen's protective-efficacy, volunteers will be challenged by the bite of 3+1 *P. vivax* sporozoites infected mosquitoes. From the 5th day after the bite, medical and parasitological monitoring will be carried out to determine the infection's appearance. All procedures in this study will be performed under GLP and GCP principles. Additionally, to evaluate the infective capacity of *P. vivax* gametocytes-infectivity during the early stages of the blood cycle, the volunteers will be exposed to the bite of 20 healthy mosquitoes every two-days from the 5th-day post-infective bite, until the moment of diagnosis or until the 15th day.

**Procedures to minimize CHMI related risks**	● Detailed analysis of the clinical history and laboratory tests to evaluate the inclusion- and exclusion-criteria of volunteers.<br>● Standard Blood Bank infectious tests on donated blood destined for mosquito infection.<br>● Closed post-CHMI hematological, parasitological, and blood chemistry follow-up will be performed at the time of diagnosis, and 28 days after the antimalarial treatment is finished; physical examination and personalized communication.<br>● Antimalarial treatment established immediately after the presence of thick blood smear (TBS) parasitemia is documented.<br>● Restriction of the entry of personnel into infected-mosquito rooms to minimize the risk of malaria transmission to the community.<br>● Immediate treatment of any individual exposed to accidental malaria infection.<br>● For the protection of health and laboratory personnel, testing of volunteers' blood samples for antibodies against HIV, hepatitis B, and hepatitis C.<br>● Standardized biosafety procedures will be followed for handling blood and body fluid samples.
**Data handling and analysis**	The data obtained from the study will be entered into an online database with the REDCap program (http://project-redcap.org/). The final processing of the data will be done using the statistical software STATA®, SPSS®, or R®.
**Study Duration**	36 months

<table>
<tr>
<td>Facilities were the trial will be conducted</td>
<td>
ASOCLINIC Inmunología
Carrera 37 2Bis No. 5E-08, Cali, Colombia
Telephone: (57)-(2)-5574929-5574921
Fax: (57)-(2)-5560141
Clinical Laboratory

Malaria diagnosis by TBS.
Screening laboratory tests: blood chemistry, hematology tests, and urinalysis.
IFAT P. vivax serology.

CIV/MVDC
Km 6 Vía Cali – Puerto Tejada. Corregimiento el Hormiguero
Telephone: (57) (2) 5216228/521 4060
Clinical Trials Unit

Site for recruitment of volunteers.

Center for Applied Biotechnology (CAB)
Corregimiento el Hormiguero, Km. 6 Vía Cali- Puerto Tejada. Tel: (57) (2) 521 6228/ 521 4060
Cali, Valle del Cauca
Entomology Unit:

Facility for infected mosquitoes challenge

Imbanaco Medical Center (IMC)
Carrera 38 A No. 5 A 100. Cali, Colombia.
Telephone: (57)-(2)- 6821000
Fax (57)-(2)-5186000

Health provider for medical care in the emergency room, hospitalization, and surgery, if required.
Place where the volunteers' immunizations will take place

</td>
</tr>
</table>

.

3. INTRODUCTION

Malaria produces approximately 230 million clinical cases/year worldwide, of which about 1 million results in deaths (WHO, 2010). Because of the failure of classical control measures, such as the use of insecticides and antimalarial drugs, in the last two decades, intensive work has been done to identify molecules that can be used as additional control strategies as vaccines. Considerable evidence supports their feasibility: 1) Individuals from endemic areas become clinically immune through repeated malaria exposure. 2) Passive transfer of specific antibodies or immune cells has been shown to confer protection on non-immune individuals. 3) The protective efficacy of several vaccine candidates against *P. falciparum* has recently been documented.
Although there are numerous experimental vaccines against *P. falciparum*, only a limited number of *P. vivax* antigens have been described. The most studied is the Circumsporozoite Protein (*Pv*CS) and the *Pv*s25 protein expressed in ookinetes. Our group has immunologically characterized the CS protein and has carried out both preclinical studies in primates and phase Ia clinical trials. Long synthetic peptides (LSP) derived from this protein have been used. A model for infection of human volunteers has been established using infected mosquitoes carrying mature and viable sporozoites. This infectious challenge model allows the evaluation of the protective efficacy of *P. vivax* vaccines in humans.

3.1 MALARIA EPIDEMIOLOGY

Malaria is a disease that affects approximately 230 million people. It is responsible for 1 million deaths per year, representing a substantial economic impact for populations living in developing regions, especially in sub-Saharan African countries (Breman et al., 2001; Sachs and Malaney, 2002), but also in some areas of Asia and Latin America (LA). Epidemiological indicators report that *P. vivax* infections remain widely distributed worldwide, even more than the ones due to *P. falciparum,* so that they create a significant cause of morbidity and mortality among the 2.85 billion people living at the risk of infection (Guerra et al., 2010). The majority of *P. vivax* cases are reported from Southeast Asia and the West Pacific regions and account for about ~ 70% in LA and a lesser extent (5-20%) in some African countries (Guerra et al., 2010; Mendis et al., 2001).

P. vivax malaria incidence has been increasing over the last 30 years and resistance to standard antimalarial therapy (Guerra et al., 2010). Also, in most endemic areas, both *P. falciparum* and *P. vivax* share the same vector. Therefore, *Anopheles* insecticide resistance affects the transmission and control of the two species of *Plasmodium* (Rodriguez et al., 2009). On the other hand, although *P. vivax* disease is less lethal than *P. falciparum's*, the development of silent or latent hepatic parasitic forms (hypnozoites) makes it more complicated since they constitute a reservoir that could lead to periodic reactivations (Sattabongkot et al., 2004).

3.2 VACCINES AS MALARIA CONTROL ALTERNATIVES.

Due to the flaws in classical malaria control strategies described before, vaccines are considered

a complementary strategy. Over the past two decades, ~ 30 *P. falciparum* antigens have been identified, and their immunogenicity and protective efficacy have been assessed in animals and humans (Richie and Saul, 2002). Some of these antigens are differentially expressed in sporozoites (CSP, SSP2 / TRAP) (Rogers et al., 1992), during the hepatic stage (LSA1, LSA3, EXP1), in the erythrocytic phase (MSP-1, MSP-2, AMA-1), and the sexual forms (*Pf*s25, *Pf*s45/48) among others. Most of these antigens have been identified by genomic libraries screening, by epitope-mapping using sera against complete parasites or parasite fractions as well as monoclonal antibodies (Mabs). Platforms for vaccine development encompass, among others, synthetic peptides and recombinant proteins formulated in different adjuvants, live recombinant viruses, and DNA vaccines. The immunogenicity and protective efficacy of various malaria antigens have been experimentally tested in animals and humans (Kumar et al., 2002), displaying a wide range of immunogenicity and/or protection (Genton and Corradin, 2002). Among the vaccine candidates under development, the RTS,S/ASO2A, a recombinant vaccine hybridized with hepatitis B surface antigen, has shown significant protection in clinical trials and currently represents the most advanced experimental vaccine. Phase I/IIa studies have indicated its safety immunogenicity and protective efficacy in healthy volunteers from the United States (Stoute et al., 1997). Furthermore, recent Phase IIb trials carried out in Mozambique reaffirmed this vaccine is highly immunogenic and produces protection in semi-immune individuals with a decrease in the development of severe disease in African children (Bojang et al., 2001; Macete et al., 2007; Stoute et al., 2006; Stoute et al., 1998). More recently, the same vaccine was evaluated in Phase III multicenter trials that included Burkina Faso, Ghana, Gabon, Kenya, Malawi, Mozambique, Tanzania, and other African countries (http://www.kemri-wellcome.org/projects/99). These studies and other ongoing studies have conferred great expectations about the feasibility of developing antimalarial vaccines based on subunits of the parasite (Alonso et al., 2004).

3.3 LIMITATIONS FOR *P. VIVAX* MALARIA VACCINE DEVELOPMENT.

In contrast to *P. falciparum's*, only a limited number of *P. vivax* antigens has been described, including MSP1 (del Portillo et al., 1991), AMA1 (Thomas et al., 1994), MSP3, MSP4, MSP5, RBP, and DBP on the asexual blood stages (Barnwell and Galinski, 1995; Barnwell et al., 1999; Chitnis, 2001; Galinski et al., 1999; Galinski et al., 2001; Miller et al., 1977); *Pv*s25 and *Pv*s28 on ookinetes/oocysts; and the *Pv*CSP and *Pv*SSP2/TRAP antigens on the pre-erythrocytic phase (Templeton and Kaslow, 1997). Of this last phase of the cycle, only *Pv*CSP has been extensively analyzed in preclinical and clinical studies (Arévalo-Herrera et al., 2010).

The limited availability of *P. vivax* antigens, in general, is explained by several factors, such as 1) the impossibility of obtaining continuous (*in vitro*) cultures of the parasite's blood forms, which would allow experimental mosquito infection; 2) difficulties for achieving a constant and adequate sporozoites production for CHMI studies; and 3) the absence of a *P. vivax* radiation attenuated sporozoites (RAS) vaccine model, which in the *P. falciparum'*s case has represented a valuable system for improving the current understanding of the immune response. Despite these hardships, our group has made progress in Colombia developing a *P. vivax* infectious challenge model and has carried out two successful studies that have demonstrated its safety and reproducibility for the evaluation of different malaria vaccine candidates efficacy (Herrera et al., 2011b; Herrera et al., 2009c). Simultaneously, it has concentrated efforts on *Pv*CS development as a vaccine candidate, a protein orthologous to the *Pf*CS, which is a component of Pf-RTS,S. The *Pv*CS, and is the subject of the present study.

3.4 BACKGROUND AND RATIONALE

3.4.1 Development of *P. vivax malaria* natural immunity.

Like *P. falciparum*, exposure to repeated *P. vivax* infections in a short period leads to clinical immunity development. Individuals from highly endemic regions develop a moderate degree of immunity between 10 and 15 years of age, which is associated with a decrease in the appearance of the infection's clinical manifestations (Cattani et al., 1986b). In other regions where the prevalence of *P. vivax* is low, highly effective immunity is not achieved. On the other hand, although some epidemiological studies indicate that *P. vivax* infection could confer some immunity against *P. falciparum*, there is not enough evidence suggesting a cross-protection between *Plasmodium* species (Gunewardena et al., 1994; Williams et al., nineteen ninety-six). Furthermore, studies in which controlled human malaria infection has been experimentally induced have indicated that immunity is acquired in a species-specific manner (Collins and Jeffery, 1999). Because *P. vivax* biological properties differ from *P. falciparum*, it is unlikely that a heterologous vaccine will control the two parasite species. Therefore, the identification of *P. vivax* components should be used for species-specific vaccine development.

3.4.2 Identification and characterization of the CS protein

The CS proteins of *P. falciparum* and *P. vivax* were identified using sera from individuals immunized with irradiated sporozoites (Druihle et al., 1998; Hoffman and Doolan, 2000). The *P. vivax* CS was identified, and its gene was cloned in 1985 (Arnot et al., 1985). The chemical characterization of the protein indicated that its structure is similar to that of other *Plasmodium* species (Sinnis and Nussenzweig, 1996). The *P. vivax* CS protein is composed of 373 amino acids. It has a central domain (90-261 a. A.) made up of short repetitive units flanked by non-repetitive protein fragments at their amino (N) and carboxyl (C) terminals. The flanking regions contain small, highly conserved sequences called Region I (85-89aa) and Region II-plus (338-355aa). They have been identified as parasite-binding domains for the invasion of hepatocytes by sporozoites (Cerami et al. ., 1992; Frevert et al., 1993). The central domain of the protein is composed of 19 blocks of 9 amino acids each, of which two allelic forms can be found in nature, the VK210 or common type (GDRADGQPA) (Arnot et al., 1985) and the VK- 247 or variant type (ANGAGNQPG) (Tsuji and Zavala, 2001). In addition to this dimorphism, a limited polymorphism has been observed in the regions encoding the amino flanks amino (N) and carboxyl (C) of the protein. (Arnot et al., 1990; González et al., 2001; Kain et al., 1992; Machado and Povoa, 2000; Maheswary et al., 1992; Mann et al., 1994; Qari et al., 1992; Rosenberg et al., 1989; Wirtz et al., 1987). This polymorphism does not seem to significantly influence the immunogenic regions (epitopes) of the protein.

During the last two decades, different research groups, including ours, have carried out the extensive immunological characterization of this protein, using sera from individuals immunized with irradiated sporozoites and semi-immune individuals from endemic areas. These individuals antibodies recognize the CS protein and induce a precipitation reaction on the surface of living sporozoites (CSP reaction) (Cochrane et al., 1976). This leads to the neutralization of sporozoites' invasion into the hepatocytes (Nussenzweig et al., 1969). Different B epitopes have been

identified throughout the CS protein entire sequence using these sera (Arevalo-Herrera et al., 1998; Franke et al., 1992a). Sera from immune individuals from different endemic areas has recognized the VK-210 and VK-247 sequences, indicating their wide distribution (Arevalo-Herrera et al., 1998; Burkot et al., 1992; Cochrane et al., 1990; Franke et al., 1992b; Ramasamy et al., 1994; Wirtz et al., 1990). VK210 contains the AGDR sequence that is highly recognized by sera of individuals from malaria-endemic communities. Also monoclonal antibodies are capable of protecting Saimirí monkeys against challenge with infective *P. vivax* sporozoites (Charoenvit et al., 1991).

Multiple T helper cell epitopes have also been recognized in the context of Major Histocompatibility Complex (MHC) class II molecule haplotypes (Herrera et al., 1994; Nardin et al., 1991). Using nona- or deca-peptides containing binding motifs for MHC class I antigens, our group identified in the *P. vivax* CS protein five peptide sequences capable of stimulating human CD8+ lymphocytes from HLA-A*0201 individuals. These peptides induced the production of IFN-γ, a cytokine involved in protection against malaria, by mononuclear cells from individuals previously naturally infected with *P. vivax* malaria (Burkot et al., 1992; Franke et al., 1992b).

3.4.3 Development of the *P. vivax* CS protein as a malaria vaccine candidate

In 1987 the *P. vivax* CS protein was initially proposed as a vaccine candidate by R. Nussenzweig's group at New York University. It was tested as a recombinant protein (rPVCS-1) in mice that produced a strong neutralizing antibody response (Cattani et al., 1986b). Two clinical trials using recombinant proteins were later conducted but failed to induce significant immune responses, thus halting the motivation to continue the development of a CS protein-based vaccine candidate (Gunewardena et al., 1994; Williams et al., 1996); and during the next decade, no more clinical trials with this protein were reported.

3.4.4 Preliminary preclinical studies in Colombia and other countries

During the last years, the CIV has concentrated significant efforts on the *P. vivax* CS protein (described above) to obtain a rational design of the vaccine (Arevalo-Herrera and Herrera, 2001). Using the epitopes identified in the protein, a series of Multi Antigenic Peptides (MAPs) were constructed containing various B and Th epitopes. Two of the 7 MAPs designed produced strong specific antibody responses against CS and IFN-γ production. However, in preclinical trials conducted in primates, these MAPs could not be purified in the amount required for subsequent clinical trials (Herrera et al., 1997). Therefore, in the framework of cooperative studies with the group of Dr. G. Corradin from the University of Lausanne (Switzerland), we decided to use the Long Synthetic Peptides (LSP) strategy with sufficient extension (> 70-mer) to contain multiple B, Th, and CTL epitopes. LSP derived from the CS protein of *P. falciparum,* and *P. vivax* were synthesized separately and tested in preclinical trials in *Aotus* monkeys (Arevalo-Herrera et al., 1998). These studies indicated high immunogenicity and *P. vivax* sporozoites' ability to reinforce this immune response (boosting) (Herrera et al., 1997). In both tests, the animals produced high titers of specific antibodies capable of recognizing by immunofluorescence (IFAT) the native protein and stimulating the release of IFN-γ determined by the ELIspot technique.

Simultaneously with these studies in Colombia, in the United States, the Walter Reed Institute (WRAIR) group in Silver Spring (MD) developed a chimeric recombinant protein that contains sequences of different variants of *Pv*CS. The recombinant vaccine formulated in Montanide ISA

was highly immunogenic in mice, and sera recognized the CS protein from *P. vivax*-infected individuals (Yadava et al., 2007).

Our group developed a new preclinical study to test the vaccine immunogenicity in BALB/c mice and *Aotus* monkeys (Arevalo-Herrera et al., 2011a). For these studies, combinations of the three synthetic peptides corresponding to the amino (N), central (R), and carboxyl (C) regions of the CS protein were used, formulated in the adjuvants Montanide ISA 720 or Montanide ISA51. Both formulations were highly immunogenic in both species. Mice developed better antibody responses against C and R polypeptides, while N polypeptide was more immunogenic in primates. These studies stimulated progress towards the clinical development of this protein.

3.4.5 *P. vivax* CS protein phase I clinical trials

Based on these results, the CIV decided to initiate Phase I clinical studies to determine the safety, tolerability, and immunogenicity of 3 different LSP derived from the *Pv*CS protein in human volunteers. More recently, clinical studies aimed at standardizing a method to infect healthy volunteers with viable sporozoites in preparation for the development of Phase II trials to test vaccine protective efficacy. These trials were monitored by the WHO and financially supported by Colciencias, the Ministry of Social Protection, the National Institute of Health of the United States (NIH / NIAID); and are briefly described below.

First Phase Ia clinical trial

The safety, tolerability, and immunogenicity of *Pv*CS-derived LSPs were evaluated in a randomized, double-blind study. Sixty-nine healthy volunteers without exposure to malaria who met the inclusion criteria were immunized with three synthetic peptides corresponding to different regions (Amino = N; Central = R, Carboxyl = C) of the *Pv*CS protein formulated in the adjuvant Montanide ISA720 (Seppic, Inc). The three peptides administered in staggered doses of 10 µg, 30 µg, and 100 µg were safe, well-tolerated, and highly immunogenic (Herrera et al., 2005). The volunteers had minor signs and symptoms at the injection site, and none developed any serious or severe adverse event (AE). Although there were differences in the titers of antibodies against the different peptides, all the individuals were seroconverted (ELISA), and the antibodies recognized the native CS protein in sporozoites (IFAT). They demonstrated their ability to block sporozoites' invasion into viable liver cells (ISI).

Our group then subsequently performed a study on the cellular and humoral immune responses in 21 of the 69 patients in this clinical trial (Arevalo-Herrera et al., 2011b). The antibodies were predominantly IgG1 and IgG3 isotypes. They recognized some parasitic protein domains (IFAT) and partially blocked sporozoite invasion into hepatocyte lines in vitro. Most of the volunteers displayed a high antibody response, transmission-blocking activity, and the induction of IFN-γ production in vitro by the peripheral blood mononuclear cells, thus providing evidence for further studies. Therefore, the success of this first phase I clinical trial was successful prompted a new trial to optimize the vaccine formulation (Herrera et al., 2011a).

Second Phase Ia clinical trial with combined peptides

A study was designed in which the safety, tolerability, and immunogenicity of the mixture of LSP formulated in two of the most potent adjuvants available at the moment for use in humans were determined: Montanide ISA-720 and Montanide ISA-51 (Seppic, Inc) (Herrera et al.,

2011a). This clinical trial was proposed to identify which of these two adjuvants generated a better formulation to be used in subsequent Phase II trials.

For this trial, 40 healthy volunteers not previously exposed to malaria were randomized to receive three injections of placebo or the mixture of synthetic peptides N, R, and C at doses of 50 µg or 100 µg/dose/peptide formulated in the Montanide adjuvants already described. The first immunization consisted of a mixture of the N + C peptides. The second and third immunizations were mixtures of the N + C + R peptides, and the trial was designed as a controlled, randomized, double-blind, step-dose clinical study. Once again, the vaccines were well tolerated, and there was no association with serious or severe AE.

The antibody response determined by ELISA again showed seroconversion in all individuals, but peptide N, as in the previous test, induced earlier antibodies and higher titers. Although the response against peptides C and R appeared later, 97% of the volunteers responded against these peptides after the second immunization. The confirmation of the safety, tolerability, and immunogenicity of these formulations stimulated the initiation of studies oriented to establishing an infectious challenge model with sporozoites to be used in trials of the protective efficacy of the vaccine.

3.4.6 Infectious challenge models for *P. falciparum* and *P. vivax*

The possibility of infecting human volunteers experimentally with the malaria parasite has been a practice that has been carried out for 100 years (Grassi et al., 1899) (Fairley, 1947). *P. vivax* infection was routinely done as a treatment for neurosyphilis ("malaria-therapy") during the 1950s-60s (Glynn et al., 1995). Later, a challenge model with *P. falciparum* and *P. vivax* sporozoites was developed and used to test the protective efficacy of the radiation attenuated sporozoite vaccine (Clyde, 1975; Clyde et al., 1973; Egan et al., 1993; Herrington et al., 1991; Rieckmann et al., 1979). The *P. falciparum* challenge model has been used extensively to determine the protective efficacy of various vaccines in major research centers around the world: the United States Naval Medical Research Center (NMRC) (Rockville, MD) under the direction of Dr. S. Hoffman (Hoffman et al., 2002), the Center for Clinical Vaccinology and Tropical Medicine, Oxford University (Walther et al., 2005) and the Department of Medical Microbiology of the Nijmegen University Medical Center, The Netherlands. However, the challenge with *P. vivax* sporozoites has not been reported in the last three decades. For this reason, the CIV has invested significant effort in developing this system within biosafety control conditions, protection of volunteers, and statistical significance.

3.4.7. Standardization of a challenge model with *P. vivax* sporozoites

Due to the lack of continuous *P. vivax in-vitro* cultures, the infection procedure has been standardized using field isolates from infected patients to feed *An. Albimanus* mosquitoes adapted to laboratory conditions in the insectaries of the CIV in Cali. Using these mosquitoes, their susceptibility to experimental infection by different *Plasmodium* species was determined, achieving the establishment of optimal conditions to achieve experimental infections (Hurtado et al., 1997). In a first challenge clinical trial, 17 out of 18 volunteers were successfully infected using *P. vivax* sporozoites administered by biting batches of 2 to 10 mosquitoes. All infected volunteers developed pre-patent periods between 9-11 days, determined by the thick blood smear (TBS) and PCR. All volunteers were treated immediately after the diagnosis was confirmed. Volunteers were closely followed for early detection of AEs, which were most frequently related

to mosquito bites, were mild, and resolved within 24 and 72 hours (Herrera et al., 2009a; Herrera et al., 2009b). A second challenge clinical trial determined the reproducibility of *P. vivax* infection. In this trial, 24 healthy volunteers were randomly assigned to groups 1, 2, and 3, who were challenged with 3 ± one mosquito bites infected with three different parasites' isolates. The infection occurred this time in all volunteers with pre-patent periods similar to those of the first study, confirming the challenge system's reliability (Solarte et al., 2011). Again, the volunteers were treated as soon as the peripheral blood infection was diagnosed.

3.5 SCIENTIFIC JUSTIFICATION.

Except for the African continent, most of the world's endemic areas, including Colombia, present simultaneous transmission of both *P. falciparum* and *P. vivax*. For this reason, despite the important and valuable advances in the development of vaccines against *P. falciparum*, identification and production of antigenic components of *P. vivax* are required both for specific monovalent vaccines and for the production of multi-species vaccines. Considerable evidence indicates the feasibility of developing a vaccine against malaria. First, permanent exposure to malaria infections in endemic areas induces significant levels of immunity that prevent the development of severe and complicated malaria, and although this does not entirely prevent them against disease, it significantly decreases mortality from malaria (Cattani et al., 1986a; Gunewardena et al., 1994); Second, malaria-infected patients can be cured by passive transfer of malaria-specific immunoglobulins (Gysin et al., 1996); Third, sterile immunity can be achieved in immunized animals and humans (> 90%) with radiation-attenuated sporozoites of various species of parasites, including *P. falciparum* and *P. vivax*; Fourth, a significant number of trials conducted during the last decade have demonstrated the *P. falciparum* RTS,S vaccines protective capacity both in individuals with and without previous malaria exposure in endemic communities (Guinovart et al., 2009; Macete et al. al., 2007), thus currently representing the most advanced experimental vaccine. Fifth, although in the case of *P. vivax*, vaccine research is much more incipient, the CIV has successfully developed several clinical trials with the *Pv*CS (Herrera et al., 2011a; Herrera et al., 2005). The center has developed an infectious challenge system (Herrera et al., 2011b; Herrera et al., 2009c) that supports the proposed study's feasibility and potential success. *P. vivax* has unique biological characteristics and is known to cause infections and disease with clinical manifestations other than *P. falciparum*, among which relapses represent one of the essential aspects in the maintenance of *P. vivax* transmission (Collins et al. al., 1996). This species of the parasite develops forms that remain hibernating (hypnozoites) in the liver and is periodically reactivated, contributing to the burden of the disease and the increase in the transmission of the parasite. For this reason, inhibition of parasite development at the liver level during the asymptomatic phase of parasite development is ideal.

In this sense, the pre-clinical and Phase I clinical trials carried out in the CIV using peptides derived from the *Pv*CS that have proved to be safe, well-tolerated, and highly immunogenic fully justify their continuity towards the phase II trials proposed in this proposal.

Additionally, this assay represents a unique opportunity to evaluate *P. vivax* gametocytes' infective capacity in *Anopheles* mosquitoes by direct exposure to them to test the hypothesis that these forms of the parasite (gametocytes) develop in the early stages of the blood cycle. Even before being microscopically detectable in peripheral blood, from day five post-infection.

4. HYPOTHESIS

The application of peptides N, R, and C of *P. vivax* CS protein formulated in Montanide ISA-51 adjuvant offers protection against malaria infection in naïve individuals and previously exposed to malaria.

- Immunization with *Pv*CS protein is safe in volunteers previously exposed to malaria.
- The immunization of volunteers previously exposed to malaria with *Pv*CS protein produces a reinforcing effect on the previous immune response present in these volunteers.
- Vaccination with *Pv*CS in individuals previously exposed to malaria induces a response that may offer sterile immunity.
- *P. vivax* gametocytes develop early in the parasite's blood cycle, even before it is microscopically detectable in peripheral blood. These gametocytes are capable of infecting *Anopheles albimanus* mosquitoes.

5. OBJECTIVES

5.1 General objective

To determine the protective efficacy induced by the *Pv*CSP vaccine formulated in the adjuvant Montanide ISA-51 in naïve volunteers and volunteers previously exposed to malaria.

5.2 Specific objectives

1) Confirm the safety of the vaccine in naïve volunteers immunized with the *Pv*CS.
2) To determine the immunogenicity of *Pv*CS in individuals previously exposed to malaria.
3) To determine the protective efficacy of the vaccine against the infectious challenge with viable *P. vivax* sporozoites in the previous groups.
4) To evaluate the infective capacity of gametocytes in the early stages of the blood cycle of *P. vivax* in *Anopheles albimanus* mosquitoes.

6. STUDY POPULATION

6.1 Steps 1 and 3. Volunteers immunization and infectious challenge

A total of 32 volunteers (naive and semi-immune subjects from non-endemic and malaria-

endemic regions, respectively) who meet the following inclusion criteria and do not have any exclusion criteria will be enrolled in the study.

Naïve Group

Criteria for INCLUSION of volunteers

- Healthy men and non-pregnant women between 18-45 years old.
- Freely and voluntarily sign an informed consent, accompanied by two witnesses who will also sign.
- Have no history of malaria infection.
- For women, use of an adequate contraception method from the beginning of the trial until one of the study physicians lift the contraception prescription at the end of the study.
- Accept not to travel to areas considered endemic for malaria during the infectious challenge period (1 month) (Buenaventura, Tierralta, Quibdó, Tumaco, Urabá, and Bajo Cauca).
- Be reachable by phone throughout the study period.
- Being Duffy positive (Fy +).
- Hemoglobin (Hb) levels> 11 g / dl.
- Participation availability during the period in which the study will take place.
- Not be participating in another clinical study.

Criteria for EXCLUSION of volunteers

- Age under 18 or over 45.
- Women with pregnancy confirmed by a laboratory test, lactating, or with plans to be pregnant from the moment of recruitment.
- Negative Duffy phenotype.
- G6PD deficiency.
- Any hemoglobinopathy.
- Personal history of allergies to medications or insect bites.
- History of malaria infection.
- Have received vaccination against malaria.
- Clinical or laboratory abnormalities determined by the investigator (s).
- IFAT> 1:20 for P. vivax in screening tests.
- Living in a malaria-endemic region for six months before the study.
- Clinical or laboratory evidence of systemic disease, including kidney, liver, cardiovascular, pulmonary, psychiatric, or other diseases, may negatively impact and alter study results.
- Evidence of active hepatitis B and C or HIV infection.
- History of transfusion of any blood product in the 6 (six) months before the study.
- Plan to have surgery from the recruitment period to the end of the post-challenge follow-ups.
- Presence or history of autoimmune disease (lupus, rheumatoid arthritis, thyroiditis, or other).
- Splenectomized volunteers.
- Volunteers in treatment with drugs with activity on the immune system (steroids, immunosuppressive agents, or immunomodulators). A history of alcoholism or drug abuse is defined as a habit that interferes with the individual's normal social functioning.

- Any condition that may interfere with the ability to provide free and voluntary Informed Consent.

<u>Semi-immune group</u>

Criteria for INCLUSION of volunteers

- Healthy men and non-pregnant women between 18-45 years old.
- Freely and voluntarily sign an informed consent, accompanied by two witnesses who will also sign.
- Have a history of previous malaria infection (s) and positive *P. vivax* serologic tests.
- For women, use of an adequate contraception method from the beginning of the trial until one of the study physicians lift the contraception prescription at the end of the study.
- Accept not to travel to areas considered endemic for malaria during the infectious challenge period (1 month) (Buenaventura, Tierralta, Quibdó, Tumaco, Urabá, and Bajo Cauca).
- Be reachable by phone throughout the study period.
- Participation availability during the period in which the study will take place.

Criteria for EXCLUSION of volunteers

- IFAT negative (<1:20) for *P. vivax* in screening tests.
- The other criteria applied to naïve volunteers, except the antecedent of having lived in an endemic area during the last six months.

6.2 Step 2. Donation of infected blood

A total of 5-15 *P. vivax* infected patients who seek attention in malaria diagnostic centers located in Cali, Buenaventura, or other endemic areas, with detected parasitemia ≥ 0.1% and who meet the inclusion criteria of donors of the parasite, and do not meet any exclusion criteria will be included in the study.

Inclusion criteria for blood donor volunteers

- Healthy men and non-pregnant women between 15-60 years old.
- Have a positive diagnosis of *P. vivax* malaria determined by TBS examination.
- The patient must not have non-*P .vivax* circulating malaria parasites such as *P. falciparum* or *P. malariae*.
- Have a parasitemia ≥ of 0.1% per TBS.
- Hb ≥9 gr/dL at the time of malaria diagnosis.
- The patient must have the ability to provide informed consent freely and voluntarily. If you are illiterate, you can affirm your decision to participate by putting your fingerprint on the consent form. Minors who are between 15 and 17 years old and wish to participate must sign the informed consent, and one of their parents must sign the informed consent, accompanied by two witnesses who will also sign.

EXCLUSION criteria for blood donor volunteers

- Having chronic or acute illnesses, other than *P. vivax* malaria.
  Have a history of diseases or clinical conditions, which according to medical criteria may significantly increase the risk associated with participation in this study.
- Hb levels <9 g/dL at the time of recruitment.
- Have received anti-malarial treatment before diagnosis.

7. STUDY DESIGN

We propose to conduct a Phase IIa/b randomized, controlled, and double-blinded clinical trial, with 32 healthy adult men and non-pregnant women, grouped as follows: 16 volunteers with previous *P. vivax* malaria infection and 16 volunteers without malaria history (naïve). The 16-pre-immune volunteers will be selected from subjects who inhabit Colombian endemic areas, with malaria history and/or presence of anti-*P. vivax* blood-stage parasite antibodies > 1:20 by IFAT.

This study will be conducted in the CIV in Cali and will have a 36 months duration. The volunteers will be immunized at months 0, 2, and 6 with the LSP mixture (150 µg) or with placebo, formulated in the adjuvant Montanide ISA-51 (Figures 1 and 2).

The specific immune response against *P. vivax* will be evaluated in all volunteers before the first immunization and after each immunization at months 0, 1, 2, 3, 6, 7, and 8. Moreover, follow-up will be performed every 2 months until trial finalization to observe the antibody-titers evolution and their functional activity, as well as the induction of some specific cytokines like IFN- γ, TNF-α, IL-2; IL- 4, IL-6, IL-10, and to characterize the Monocytes (MO), T cells (TC) and B cells (BC). Furthermore, volunteers will be subjected to renal, hepatic, and hematologic function and pregnancy tests to determine any vaccine biological safety changes. Also, antibody functionality will be assessed *in vitro* through the Inhibition of Sporozoite Invasion (ISI) assay to Hep-G2 cells.

Thirty days after the last immunization, volunteers will be infected with *P. vivax* viable Sporozoites through infected mosquito biting (2-4 bites). The vaccine-induced protection will be determined, taking into account the prepatent period. The study will be divided into three steps as described next.

7.1 Step 1: Volunteers selection and immunization.

Recruitment

All study participants must meet the inclusion criteria and be excluded if they acquire any exclusion criteria. During the recruitment period, only the Health and Social Security General System affiliated subjects will be considered, as long as they can prove through an official document that they are affiliated in any of the system regimens. The eligible volunteers must sign an Informed Consent (IC) format after being duly informed of the study purpose. Also, a comprehension test will be applied to all volunteers to assure that they have understood the study nature, the risks and benefits related to participation and that they are signing an IC format on their free will; the evaluator will explain any element not fully understood. All IC procedures and volunteer questionnaires will be documented in the volunteer folder. Besides the IC for the vaccine trial, an IC for collecting a blood sample to perform HIV testing will be obtained. In the case the subject turns to be HIV positive, he will be informed and redirected to obtain counseling and treatment. A copy of the IC will be given to all volunteers.

Identification
A 5 characters identification code will be assigned to the study volunteers: the first character will be number 1, which corresponds to Step 1; the second and third characters will be the initials of the first name and surname followed by the number of inscription. For example, a volunteer named Mario Perdono, who is the third person recruited, will be assigned the identification code 1MP03.

Selection
The selection procedures (clinical history, physical examinations, and blood samples obtention) will be carried out only after the volunteer has signed the IC. In the case, a volunteer has been selected, and the immunization program has not started within the next 12 weeks, the selection tests will be repeated. In the selection visit, the medical antecedents and the concomitant treatment will be documented by one of the clinical investigators. A complete physical examination will be carried out, including sense organs, cardiovascular (CV), pulmonary, neurological, gastrointestinal (GI), musculoskeletal and dermatologic systems. We will take urine samples and 35 ml-blood samples to perform lab-screening-tests. Should a woman be considered for the study, she will be asked to use a contraceptive method during the clinical trial. At the end of the trial, she will be subjected to a pregnancy test.

Dr. José Millán Oñate, an infectious diseases medical doctor of the CIV, will be in charge of volunteers' clinical and paraclinical assessment during recruitment and selection processes. The following volunteer screening tests will be performed at ASOCLINIC within 12 weeks before the first immunization:

Hematologic Tests:
Hemogram, G6PD determination, Duffy phenotyping, hemoglobin electrophoresis, Rh and ABO group assessment, erythrocyte sedimentation rate (ESR), and C-reactive protein (CRP)

Renal function tests:
Urinalysis, creatinine, and blood urea nitrogen (BUN)

Infectious diseases other than *P. vivax:*
HIV (two rapid tests), Hepatitis B surface antigen (HBsAg), Hepatitis C Virus (HCV), Human T Lymphotropic Virus (HTLV) 1 and 2, RPR test for syphilis, and rapid test for Chagas disease.

Confirmatory tests:
If the result of any of the HIV-rapid tests is positive (or both), a confirmatory test will be performed: Western Blot.
If RPR is positive for syphilis (at any dilution), an FTA-ABS test will be performed.
If the HBsAg is positive, the Hepatitis B Core Antibody (Anti-HBc) test will be performed.

Hepatic function tests:
ALT, AST, total bilirubin, conjugated bilirubin, PT, and PTT.

Pregnancy test:
Urine and serum β-HCG determination

Others:
Blood glucose, electrocardiogram

Immunological tests:
IFAT Anticuerpos antimaláricos ANAs
(Anticuerpos Antinucleares)

Group constitution
We will recruit a total of 32 persons between 18 and 45 years of age who willingly accept to participate in the trial by signing an IC format. Two volunteers' main groups will be identified: **naïve** (group A, n = 16) and **pre-immune** (group B, n = 16); and randomly distributed in 4 subgroups as follows: two experimental subgroups (A1 and B1) of 10 subjects each, and two control subgroups (A2 and B2) of 6 subjects each. The volunteers will be stratified, as shown in Table 1.

Table 1. Group constitution. Step 1

	**Experimental**	**Control**
**Naïve (A)**	A1 (n=10)	A2 (n=6)
**Pre-immune (B)**	B1 (n=10)	B2 (n=6)
**Total**	20	12

Immunization
Subgroups A1 and B1 (n = 20) will be immunized with the vaccine, and subgroups A2 and B2 (n = 12) will be immunized with placebo (See Immunization Schedule in Figure 1). The assigned vaccine preparation will be pre-coded to minimize potential selection biases and balance potential cofounders, and code translation should remain blind to the investigator who performs the vaccination and to the clinical staff of the trial (Herrera et al., 2005). Before each immunization, each volunteer will be assessed by one of the research physicians. In case of an AE report, this will be registered in a Case Report Format (CRF). The safety and tolerability of the vaccine will be determined. Subgroups A1 and B1 will receive in the first immunization a mixture of N and C peptides (50 µg/peptide; total dose 100 µg/dose) formulated in Montanide ISA 51. For immunizations 2 and 3, the vaccine will be composed of peptides N, R, and C (50 µg peptide/dose; total 150 µg protein/dose). Volunteers of subgroups A2 and B2 will be injected with saline emulsified in the same adjuvant. This phase will last for six months.

The vaccine will be prepared as described in Supplement Note 1. Using a 1 mL 25G-needle syringe, a total of 500 µL solution will be taken. The vaccine will be injected intramuscularly (IM) into the deltoid muscle of the opposite arm to that previously used for blood sampling. The infectious diseases medical doctor, Dr. José Millán Oñate, will be in charge of applying the vaccine to the volunteers during this phase.

Post-immunization follow-up

- Immediate follow-up:

Volunteers will be under direct medical observation within the next hour after immunization to detect any adverse reaction to the vaccine administration. After the 1-hour observation period, a

physical examination will be performed. Eight hours post-immunization, each volunteer will receive a phone call to check his/her physical condition. Any manifestation of an AE will be reported as described in the Adverse Event section.

- Post-immunization follow-up

Personal follow-up will be carried the day after each immunization and again one or two weeks before the next one. These follow-ups will include a new clinical evaluation and if needed, an AE report. Volunteers will be provided with all the required information (including telephone numbers), so they can contact the research team members at any time. Moreover, they will be encouraged to ask questions if something is not clear.

In this study phase, the clinical and laboratory follow-up of the immunized volunteers will be in charge of the infectious diseases medical doctor.

Figure 1. Immunization Schedule

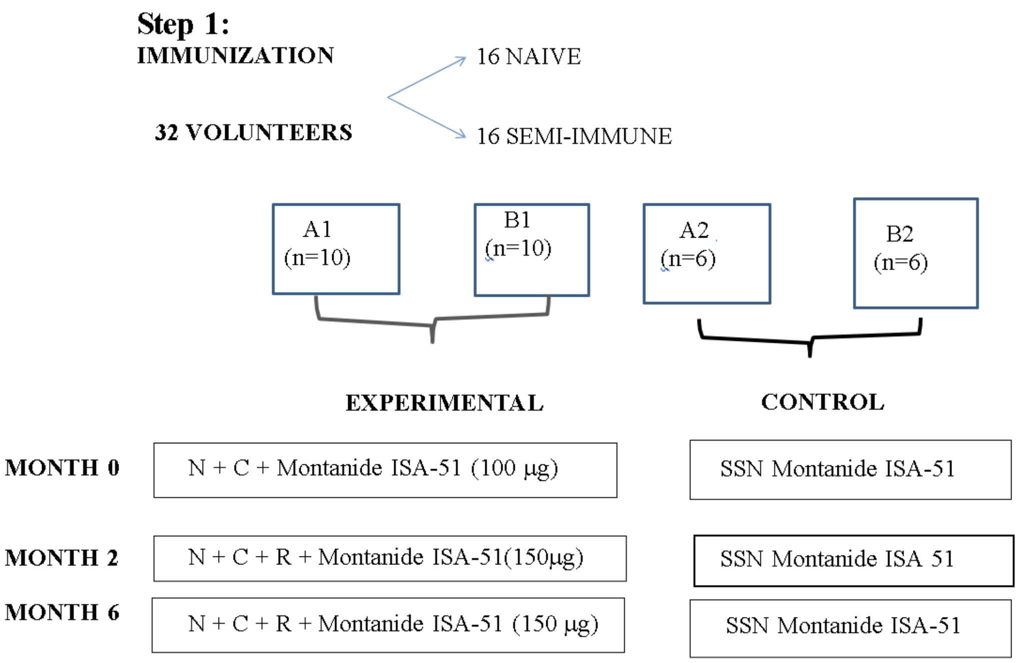

7.2 Stept 2: Donation of infected blood

In this step, *P. vivax* infected patients who attend CIV laboratories located in different endemic regions of the country or Cali (a non-endemic region) will be identified and asked to donate parasitized blood to conduct the feeding and infection of *An. albimanus* mosquitoes. Each one of the volunteers should sign an IC free and willingly. Next, 35 mL of blood from each donor will be obtained by venipuncture, which will be distributed in 5 mL that will be sent to the ASOCLINIC laboratory for the screening of infectious diseases, and a 30 mL fraction that will be used for feeding batches of adult *An. albimanus* mosquitoes.

Donors recruitment:
The patients will be attended by a certified bacteriologist or microscopist from the CIV, who will obtain and read the TBS test. If the result is positive for *P. vivax* without other co-infecting species, and if parasitemia is ≥ 0.1%, a study's physician will explain the trial methodology and objectives and invite the patient to participate in the clinical trial. If the patient agrees to participate in the study, he or she will be asked to freely and voluntarily sign two ICs; the first will be about selection and participation in the study and the second will be for conducting HIV tests. If the patient is underage (15 to 17 years old), an explanation will be given to both the minor and the father, mother, or legal guardian so that they accept the patient's participation in the study. The minor will sign an informed assent, and the IC must be signed by a parent or legal guardian. In the end, a copy of the documents will be given to the participants.

Identification:
Volunteers will be assigned a 6-character identification code: the first character will be the number 2, which will be used only for volunteers in the second step; the second and third characters will be the initial letters of the volunteer's name and surname, respectively, followed by the registration number. For example, a volunteer named Mario Perdomo, who was the tenth person selected, will be assigned the identification code 2MP010.

Blood donation:
Once the volunteer has signed the IC and an identification code for screening has been assigned, one of the research team physicians will conduct the correspondent clinical history and physical examination. This step will result in two possible scenarios:

- The subject does not meet the inclusion criteria: In this case, the subject will be offered antimalarial treatment as indicated in the Malaria Treatment National Guidelines. The reasons why he/she is not suitable for the study and therefore can not donate blood will be explained. Even if the subject is not included in the study, he/she will be asked to return two weeks later (Day 15 after starting the treatment) to perform a TBS that ensures malaria resolution. If the TBS is positive on day 15, the treatment schedule will be repeated. In the event of resistance to chloroquine, it will be managed as described in the section on treatment and follow-up (see below).
- The subject does meet the inclusion criteria: In this case, 35 mL of blood will be obtained by venipuncture, from which 5 mL will be used for screening of infectious diseases, and the remaining 30 mL will be used to perform the AMF in the CIV Entomology Unit.

Immediately after blood donation, volunteers will receive the medications, according to the current Malaria Treatment National Guidelines issued by the Ministry of Social Protection of Colombia.

Volunteers will be asked to come back to CIV or Buenaventura about one week later to claim their screening test results, including the HIV test. With a copy of them, the volunteer will be redirected to the correspondent health provider according to the health security regimen in which he/she is affiliated, so he/she can obtain counseling and medical assistance. If the volunteer has a private doctor, he/she will be remitted to him with a copy of all the results, and, if the subject is not affiliated to any social security regimen, he/she will be sent to one of the public health network hospitals attached to the Health Departmental Secretary.

Volunteers' treatment and follow-up:

- Chloroquine: 600mg (4 tablets of 150mg) on the first day, 450mg (three tablets) for the second, and 450mg (three tablets) on the third day.
- Primaquine: 30mg (2 tablets of 15mg) per day for 14 days.
- Follow-up: patients will be asked to return two weeks after starting the treatment (Day 15 after the first dose) to perform a TBS that ensures the cure of malaria. If the TBS is positive on day 15, the treatment will be repeated.

In case of resistance to chloroquine (*), the infection will be managed with the combination of sulfadoxine/pyrimethamine (Falcidar® 25mg pyrimethamine / 500 mg sulfadoxine) 3 single-dose tablets as an alternative treatment.

If the patient has a contraindication to Falcidar® (e.g. allergy to sulfa), he will receive amodiaquine in a dose of 3 tablets (each tablet has 200mg, dose of 600 mg/day), for three days (Bosman, et al., 2001), he will be asked to return a week later to obtain a TBS and confirm the cure.

(*) If resistance to chloroquine is confirmed, the batch of mosquitoes infected with such a sample will be discarded. If it has already been used for the challenge, the treatment of the volunteers in step 2 will be modified as described below.

Laboratory tests in donated blood

***Plasmodium* PCR:** 500 μL of the donor sample will be used to perform the PCR analysis for *P. vivax, P. falciparum,* and *P. malarie*, to rule out mixed malaria.

PCR has higher specificity and sensitivity values than any other currently available diagnostic method (WHO, 2000); and is considered the "Gold Standard" for malaria diagnosis in the research scenario. The test achieves sensitivities and specificities of up to 100% compared to other available diagnostic methods (Pöschl B., et al, 2009).

In the clinical setting, however, PCR is not the diagnostic method of choice since it is a test that requires expensive equipment and reagents, which is not readily available in many centers. Since it is a time-consuming test for its processing and reporting, there would be a delay in patient treatment.

This difficulty will not appear in this study since the diagnostic tool will be TBS, and as soon it turns out positive, the appropriate treatment will be immediately started.

The PCR to rule out infection by other *Plasmodium* species will be carried out on the donor samples after infecting mosquitoes and before using the infected batch for the challenge of healthy volunteers, to ensure that the blood has an exclusive presence of *P. vivax* parasites. In case PCR is positive for a non-*P. vivax Plasmodium* species, the batch of mosquitoes fed with this blood will be discarded according to biosecurity standards and will not be used for the challenge under any circumstance.

Although the possibility of PCR false negatives for *P. falciparum* is very low, if there is a positive donor for mixed malaria not initially diagnosed by PCR, this may be evidenced during the post-treatment follow-up since his symptoms will not improve with the *P. vivax* antimalarial treatment for this regimen is not effective against *P. falciparum*. At this moment, TBS and PRC would be performed again, and adequate treatment would be started against *P. falciparum.* The mosquitoes fed with said blood would be discarded.

Blood bank analysis: 5 ml of donor blood will be used to perform the following tests: Two rapid HIV tests from different commercial brands, antibodies against HTLV 1 and 2, hepatitis B surface antigen (HBsAg), rapid-test for Hepatitis C, rapid-test for Chagas disease and RPR for syphilis.

Confirmatory tests: If any of the HIV rapid tests turn positive, Western Blot will be performed as a confirmatory test. If the HBsAg is positive, anti-core hepatitis B antibodies will be assessed to confirm the diagnosis. Likewise, if the RPR for syphilis is reported positive at any dilution, an FTA-ABS test will be conducted.

Although it is theoretically possible that mosquitoes can transmit the Hepatitis B virus within 72 hours after being fed with blood (Blow, et al., 2002), no evidence has been found that any of these diseases are transmitted by *Anopheles* mosquitoes. Besides, mosquitoes have an incubation period of 15 to 18 days, during which the transmission of any of these diseases is unlikely.

Additional Tests: In addition to routine blood bank examinations, other pathogens that could be inadvertently transmitted by *Anopheles* mosquitoes during the challenge will be considered.

Discussions held before challenge trial # 1 (Herrera, et al, 2009) with experts in vector-borne

diseases indicated the following.

Filarias: Dr. Augusto Corredor, former director of the Parasitology Unit of the National Institute of Health of Colombia, and Dr. David Botero (parasitologist), professor at the Antioquia University (Medellín, Colombia) confirmed that there is no evidence of current transmission of no filarial species in Colombia and that assessment for these parasites is not necessary. These concepts were confirmed at the time with Dr. Dwayne Grubman, chief of the Section on Diseases caused by Filaria at the Centers for Disease Control (CDC) of the United States.

Other parasites: Although *Leishmania spp* is endemic to this region, they are not transmitted by *Anopheles* mosquitoes.

Viruses: Likewise, before the previous challenge trial, Dr. Jorge Boshell (virologist), former director of the National Institute of Health of Colombia, and Dr. Robert B. Tesh (MD), Professor of the Department of Pathology of the Center for Tropical Diseases at Texas University (Galveston, Texas), were consulted to determine the possible need to evaluate donated blood against viruses other than those studied in the Blood Bank tests, both consultants considered it is not necessary.

Additionally, the 1-3 year follow-up of the volunteers who were enrolled in the previous trials of our group (Herrera, et al, 2009; Herrera, et al, 2011), did not refer to any type of pathology associated with the experimental infection.

7.3 Step 3. Sporozoite obtention and infectious challenge.

Mosquito infection

Female *An. Albimanus* mosquitoes will be prepared in compliance with GLP conditions in any of the CIV insectaries located in different malaria-endemic regions or Caucaseco (Cali). The blood samples collected in step 2 will be tested to confirm the presence of *P. vivax* parasites by TBS microscopic examination, peripheral blood smear, and RT-PCR. Parasitaemia will be determined by TBS after reading 300 microscopic fields by an independently-well-trained microscopist. All selected samples should have a parasitemia above> 0.1%. Whereas RT-PCR allows precise identification of the parasite species and greater sensitivity (8-10 parasites/µl), TBS allows total quantification of parasitemia and gametocytaemia.

Blood samples will be used to infect an equal number of mosquito batches containing 10,000 mosquitoes per batch. Blood samples will be centrifuged at 3000 rpm for 5 min. at room temperature and the autologous plasma will be removed. The blood will be washed with RPMI1640 medium and will be reconstituted at 50% hematocrit with equal volumes of 1 pool of AB non-immune human serum obtained from a blood bank. The complement of serum AB will be inactivated by heating at 56ºC for 30 min. After washing the blood, 3-4 day old female mosquitoes

that have been fasted overnight will be fed using an artificial membrane feeder apparatus at 37°C, as previously described (Hurtado et al., 1997). Mosquito boxes will be labeled with a feeding code and the date of infection. The day after feeding, the females that do not have blood will be removed from the box, and the fed mosquitoes will be kept under strict biosecurity norms under conditions of temperature and humidity described in the respective Standard Operating Procedures (SOP) for the optimal functioning of the mosquito colony.

The mosquito batches fed with blood samples confirmed with *Plasmodium* coinfection (e.g. *P. falciparum*) will be discarded under biosafety conditions. In contrast, the batches fed with samples containing exclusively *P. vivax* will be kept. Samples from these fed mosquitoes will be dissected on days 7 and 8 after feeding to determine the presence of oocysts in their midgut, and on days 14 and 15 to assess the sporozoite load in their salivary glands. For oocyst analysis, 40 mosquitoes will be dissected and their midguts will be stained with 2% mercurochrome and examined microscopically, as described by Eyles (Eyles, 1950). Oocyst infection will be calculated according to the equation Nx79 / N + 79, where N is the number of live mosquitoes on the day of mosquito dissection. The results will be expressed as the percentage of infected mosquitoes and the arithmetic mean of the number of oocysts per intestine of dissected mosquitoes. Batches of positive mosquitoes will be kept inside the insectarium's biosecurity room, where they will be kept for another seven days until they are examined for the presence of sporozoites. The dissected salivary glands (6 lobes) will be mounted on a non-slip slide in a drop of PBS (Phosphate Buffer Saline) and will be crushed by the application of pressure. The salivary glands will be examined microscopically with a resolution of 400x magnification to evaluate the density of sporozoites for each pair of glands. Each preparation will be classified as 1+ (1-10 spz), 2+ (11-100 spz), 3+ (101-1000 spz) or 4+ (> 1001 spz) (Chulay et al., 1986). A total of 38 mosquitoes from each batch will be dissected and microscopically examined on day 14 to determine the presence of sporozoites in the mosquito's salivary gland.

Challenge batch exclusion criteria

- Batches infected with blood from volunteers infected with mixed malaria (*Pv + Pf*), HIV, HTVL-1/-2, Hepatitis B, Hepatitis C, Chagas, syphilis, and any other criteria determined by the researcher, such as the occurrence of therapeutic failure in the donor suggesting Chloroquine resistance of the *P. vivax* strain.
- Batches with a percentage of mosquitoes infected with < 50% sporozoites.

Challenge preparation

Volunteers will be invited to visit the Entomology Unit two (2) days before the challenge. This visit will allow them to become familiar with the place where it will take place, which will reduce the anxiety of the challenge day. At this moment, all the volunteers will be taken 35 ml of blood to assess their baseline immune status.

The female volunteers who participate in the study will be warned to immediately contact one of the study doctors if they become pregnant in the lapsus between the selection visit and the challenge moment. One day before the challenge, the women will be cited in the CIV facilities to perform a blood pregnancy test. If any of them are positive, they will be immediately excluded from the study. If pregnancy occurs, the volunteer will be replaced by one of the alternate volunteers.

On the challenge day, the volunteers will be evaluated by one of the study doctors, who will perform a clinical history and a short physical examination, including vital signs. If it is found that any of the volunteers have an acute illness, which in the opinion of the evaluator requires the exclusion of the volunteer, he/she will be replaced by one of the alternate volunteers.

Mosquitoes from the chosen batches for the challenge will be distributed in small "feeding cages" of 7x7x7 cm. Before the challenge, the needed cages will be prepared using mosquitoes from the same batch, with three mosquitoes in each.

Controlled Human Malaria Infection with *P. vivax* infected mosquitoes

According to the previously established procedure, the 32 volunteers immunized in step 1 will be challenged on day ~ 210 of the study, one month after the third immunization, by exposure to the bites of 2-4 infected mosquitoes. The procedure will be carried out in a security room within the CAB Entomology Unit.

The "feeding cages" will be placed on the volunteer's forearm for 10 minutes, allowing the feeding window that is covered by a mesh to be against the surface of the volunteer's skin.

After feeding, the volunteers will remain in the insectary. Simultaneously, the Entomology Unit technicians will observe the number of mosquitoes fed and determine the number of infected mosquitoes per cage by dissection of the salivary glands and microscopic examination to quantify the sporozoites load in the vector's glands. If the bite rate (determined by the presence or absence of blood-feeding) and the infectivity rate (determined by the sporozoite load in the salivary gland dissection) is below the minimum number of infective bites predicted in any of the volunteers, other mosquitoes will be employed, until a total of 2-4 mosquitoes are fed and infected with sporozoites located in the salivary glands. For example, if only two mosquitoes were fed on a volunteer and the remaining mosquitoes were positive for sporozoites, only one mosquito would be placed in the next exposure. With another volunteer, it could be found that only one mosquito was infected; in the following exhibition, two mosquitoes will be placed.

The volunteers will be observed directly by one of the study medical doctors for one hour after

the challenge, allowing them to detect any adverse reaction induced by the mosquito bite immediately. Approximately eight hours after the challenge, the volunteers will be checked by phone to document their progress. Volunteers will have all the information they need to contact researchers 24 hours a day (including cell phone number) and be encouraged to ask if they have questions or require any guidance. This direct contact will take place for three weeks. The clinical and paraclinical monitoring of the volunteers in this phase will be in charge of Dr. José Millán Oñate, the study's infectious diseases specialist.

Xenodiagnosis and evaluation of gametocyte infectivity:

Plasmodium vivax gametocytes' infective capacity in *Anopheles* mosquitoes will be studied by direct exposure of the vectors to them to test the hypothesis that these forms of the parasite (gametocytes) develop in the early stages of the blood cycle, even before being microscopically detectable in peripheral blood five days after the infection. For this process, direct infection and artificial membrane feeding will be compared. Mosquitoes will be used since xenodiagnoses have a greater capacity to detect the parasite than laboratory diagnostic tests, as has already been reported in other communicable diseases such as Chagas disease, trypanosomiasis, leishmaniasis, and Arbovirus infections (Mourya, Gokhale, et al. 2007; Wombou Toukam, Solano, et al. 2011). Volunteers will be exposed to the bite of 20 uninfected *An. Albimanus* mosquitoes by direct exposure according to POE EN-02-POE-003 on a procedure for a direct mosquito bite. Mosquitoes will be placed in 7 x 7 x 15 cm "feeding cages" to be placed on the forearm or in the place of preference of each individual for 10 minutes. Mosquitoes will be evaluated for the presence of oocysts on day 7 according to POE EN-02-POE-002 and the presence of sporozoites on day 14 according to POE EN-03-POE-001. Exposure to mosquitoes will be done every two days from the fifth day until the microscopic diagnosis is confirmed or until day 15 as follows: on days 5, 7, 9, 11, 13, and 15.

Post-Challenge Evaluation

<u>Monitoring of the pre-patent parasitemia period</u>: from day one to day six post-challenge, the volunteers will be followed by telephone by the study staff. Volunteers will receive instructions about malaria symptoms such as fever, headache, chills, myalgia, malaise, which can occur between days 7 and 23 after the challenge. Although parasitemia is unlikely to appear before the ninth day, the study team will be available to care for any volunteer who exhibits early symptoms of malaria.

After infection, the volunteers will remain under medical observation for an hour, during which they will be strictly monitored. In contrast, the feeding and infection of the mosquitoes are confirmed, and it is determined that no adverse reactions will occur. After this period, the volunteers will leave the Center, and ~ 8 hours later, they will be checked to document their evolution. Subsequently, a personal control will be carried out at 24 hours, and from then on, a

daily telephone follow-up will continue until day seven post-challenge. From day seven post-challenge and until day 28, the volunteers will be daily evaluated by a study medical doctor, followed up every day with TBS, and a blood sample will be taken for the subsequent performance of RT-PCR with comparative purposes at the end of the study. If the volunteer has a fever (axillary temperature > 38ºC) and/or other signs/symptoms of malaria, TBS and peripheral blood smears will be done twice a day. If a volunteer presents symptoms compatible with malaria but his thick film is negative on 3 successive occasions, a diagnostic test for malaria by RT-PCR will be performed immediately (Rougemont et al., 2004) to confirm the diagnosis. However, the gold standard for treatment should be the thick smear. If any of the volunteers who developed the infection needs to be hospitalized, they will be treated at the Imbanaco Medical Center by the infectious disease specialist who is a member of the clinical team.

If 28 days after the challenge, the volunteers are negative for malaria, they will continue with their parasitological monitoring (TBS) twice a week until day 60 after the challenge. During this period, volunteers will be daily monitored by phone. Some volunteers are expected to develop sterile immunity; however, some may be only partially protected and develop more extended pre-patent periods. Those individuals who become infected and present pre-patent periods similar to those of group C, that is, their pre-patent periods do not have a statistically significant difference from that of unvaccinated controls will be considered unprotected. If any of the volunteers develop malaria in this phase, the study's physician will be in charge of administering and monitoring the treatment.

Malaria Treatment

Once the malaria infection is demonstrated, the volunteers will be treated with the antimalarial regimen recommended by the Ministry of Social Protection for the treatment of *P. vivax*, which consist of Chloroquine (a total of 1,500 mg of oral chloroquine in divided doses: 600 mg initially, followed by 450 mg at 24 and 48 hours after the first dose) and Primaquine for fourteen days (30 mg/day) administered with food. All antimalarial drugs will be given with food, as they can cause stomach pain (gastritis), nausea, and vomiting if taken on an empty stomach. From that day on, volunteers without parasitemia at day 60 will be treated with the same antimalarial regimen. Primaquine will be administered directly and under daily medical supervision for fourteen days in the CIV.

Follow-up after initiation of antimalarial treatment

A TBS will be taken every day after chloroquine treatment until three have yielded negative results are presented consecutively. TBS monitoring will be done on days 7, 14, and 21 after the initiation of treatment to ensure cure. If a volunteer develops a fever or any symptom compatible with malaria, a TBS will be performed again on the day of symptoms and if necessary, an

alternative treatment scheme will be administrated. This therapeutic regimen has been effective in completely controlling the infection in 1-2 days in two recent trials. On day 45th, after starting antimalarial treatment, the volunteers will be evaluated at the CIV by one of the study's physicians; and 10 mL of blood will be taken to measure hematological, renal, and hepatic function and to determine any pregnancy event.

Tracking relapses or recrudescences

There are no documented cases of relapses in Colombia with supervised high doses of Primaquine (30 mg/day/14 days). In our previous studies, no relapses were observed in a 2-year follow-up, although on two occasions, there were reinfections in volunteers who visited the endemic area after the study. All volunteers will be contacted by telephone at 3-month intervals after completing Primaquine treatment and having a final negative TBS.

The resistance of *P. vivax* to Chloroquine has been documented only rarely in Colombia (Comer et al., 1968; Soto et al., 2001) and has not been observed with combined treatment (Chloroquine plus Primaquine) (Soto et al., 2001). However, in the unexpected event that a positive sample is found, at any time, during TBS post-treatment follow-up (days 7, 14, 28 after initiation of treatment), the volunteer (s) will receive alternative treatment with Fansidar® (SP); three tablets in a single dose (25 mg of Pyrimethamine plus 500 mg of Sulfadoxine per pill). If the patient has a contraindication to Fansidar ® (e.g., sulfa allergy), he or she will receive Amodiaquine as described above and will be followed-up with additional TBSs to confirm cure.

Any relapse of *P. vivax* will be repeatedly treated with Chloroquine and Primaquine (in doses identical to the first treatment scheme). The follow-up will be carried out on the same days as the first cycle, as explained. Long-term follow-up will be performed to detect possible relapses due to *P. vivax* hypnozoites. Once the supervised treatment with Primaquine has been completed (2 weeks) and negative TBSs are obtained in the post-treatment controls, all volunteers will be contacted by telephone in the periods defined in Table 2.

Additionally, the study's physician will be the clinical consultant for the rest of the team in all the aforementioned phases and the event of a relapse.

Table 2: Post-treatment telephone follow-up

		**Accepted range**
During the 1st month	Weeks 1, 2, 3, and 4	+/- 3 days
During the 2nd month	Weeks 6 and 8	+/- 5 days

During the 3rd month	Weeks 10 and 12	+/- 7 days
During 6th month	Weeks 16, 20, and 24	+/- 10 days
During the 1st year	Weeks 30, 38, 46, and52	+/- 10 days
During 1.5 year	Weeks 52, 60, 68, and 76	+/- 14 days

All volunteers exposed to the infectious challenge will be encouraged to contact the study physician or their medical health provider to advise them in cases related to malaria diagnosis and treatment and in case of fever. If fever, chills, seizures, or other malaria-related symptoms occur at any time within one and a half year (18 months) after the challenge, the physician in charge of the volunteer should be informed that he/she has been exposed to a CHMI, therefore he must have a TBS test, peripheral blood smear, and RT-PCR. A list of the study procedures, including the follow-up of the volunteers, is shown below:

Figure 2: Summary. Schedule of study procedures

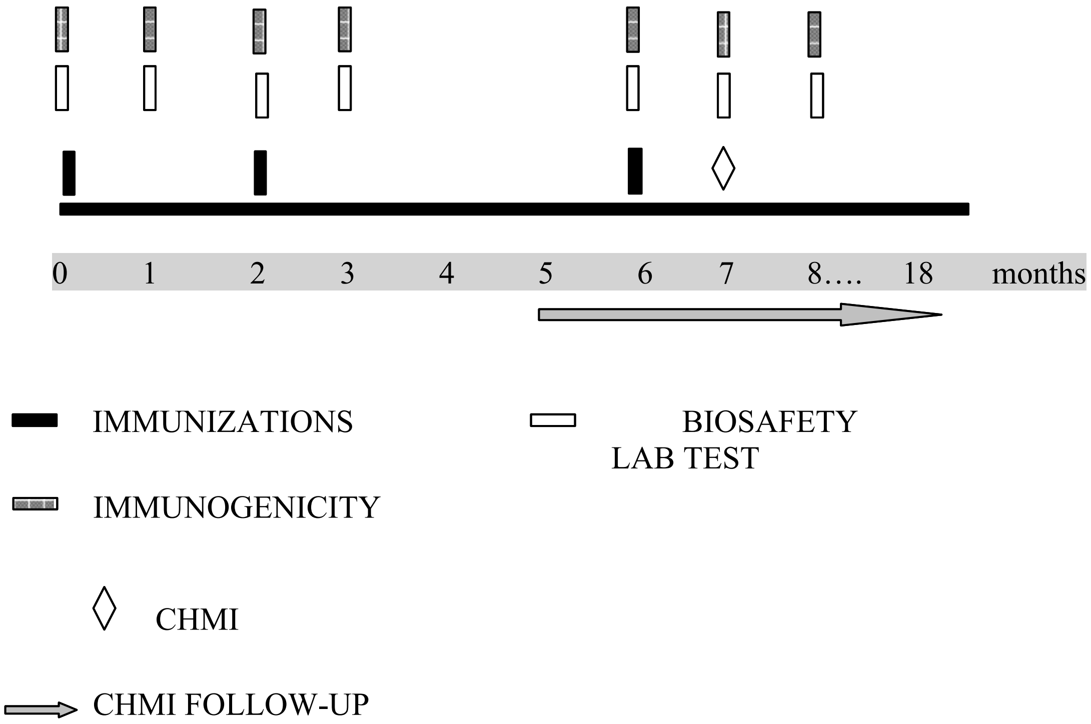

8. LABORATORY TEST

8.1 Malaria diagnosis:
The CIV and ASOCLINIC have been designated by the Valle Departmental Health Secretary as the national diagnostic center to identify malaria infection. Two independent microscopists will read each sample. For thick blood smear examinations, a total of 200 microscopic fields will be examined with oil immersion (x 1000) before reporting that no parasites have been found.

Thick blood smears: will be done in step 2 to select the donor group volunteers and step 3 in the post-challenge and post-treatment follow-up. For the tests, ~2 drops of blood will be collected by fingerstick. The TBS and peripheral smears will be colored by Field's staining method using the POE CD-POE-001-03 (ASOCLINIC) recommended by the MSP of Colombia.

Malaria RT-PCR (steps 2 and 3): A DNA extraction will be performed from whole blood to perform real-time PCR (RT-PCR) diagnosis for *Plasmodium* (*P. vivax, P. malariae,* and *P. falciparum*) (Rougemont et al., 2004), with a 500 µL sample of donor's blood. This technique is characterized by having an analytical level of sensitivity that detects up to 1 parasite/µL. For this analysis, the primers Plasmo 1 and Plasmo 2 and the species-specific TaqMan probes for *P. vivax* and *P. falciparum* will be used. For each species and assay, positive and negative controls will be used, and the standard curve will be constructed from plasmids for both species to establish and quantify the number of copies per sample.

8.2 Infectious diseases screening test:

Immunogenicity tests will include:

- Determination of native protein recognition by IFA
- Determination of specific antibodies against CS by ELISA
- Determination of T cell response by specific induction of cytokine production IL-2, IL-4, IL-6, IL-10, TNF-α, IFN-γ by ELISPOT and flow cytometry.

All serum samples will be stored at -40°C and cells will be stored in liquid nitrogen until use. The results will be archived in the laboratory for later analysis.

Methodology for objective # 1. *To confirm the safety of the vaccine in naïve volunteers and previously exposed to malaria immunized with PvCS.*

1. **Laboratory tests to establish vaccine safety.**

Complete blood count, PT, PTT, ALT, AST, bilirubin, alkaline phosphatase, BUN, creatinine, partial urine, and pregnancy test (for women) will be performed monthly to determine changes in any of the parameters of these tests. Any alteration will be considered to have a relationship

with the vaccine. If there is any alteration that requires additional clinical or paraclinical studies, these will be carried out along with those already established. Monitoring will continue until these parameters are normalized.

Methodology for objective # 2. ***To determine the immunogenicity of PvCS in individuals previously exposed to malaria.***

- **Evaluation of B cell response.** Specific antibody titers will be measured using IFAT and ELISA techniques on day 0 (zero), and subsequently, at months 1,2, 3, 6, 7, and 8, using antigens, sporozoites and synthetic peptides derived from the PvCS. Additionally, the functionality of the antibodies will be evaluated *in vitro* by sporozoite invasion inhibition assays to hepatocytes.
- **Indirect immunofluorescence / IFAT.** For this test, the antigen will be prepared using *P. vivax* sporozoites produced in *An. Albimanus* mosquitoes (Hurtado et al., 1997). IFAT tests will be performed according to the previously described protocol (Herrera et al., 2005). Briefly, the slides will be incubated in a humid chamber in the dark for 1 hour at 37°C, with 25 µl of serum diluted in PBS-BSA 2%, starting with a 1:20 dilution. After three washes in PBS, a FITC-labeled anti-human IgG (1: 100 in PBS-Evans Blue 0.05%) will be added. It will be re-washed and mounted for reading. The antibody titers will be expressed as the last dilution that showed fluorescence.
- **ELISA/enzyme-linked immunosorbent assay.** Antibodies against peptides will be measured by ELISA (Herrera et al., 2005). Briefly, 96-well plates (Nunc-Immuno Plate, Maxisorp, Roskilde-Denmark) will be sensitized with 100 µl of the synthetic peptides N, R, and C at 1 µg / mL overnight at 4 ° C. Subsequently, they will be blocked with 200 µl of PBS1X / 0.05% Tween, 5% milk for 2 hours at room temperature. Then 100 µl of each of the serum samples diluted in PBS 1 X / 0.05% Tween, 2.5% milk, will be added and incubated for 1 hour at room temperature. Subsequently, they will be washed 5 times with 1X PBS / 0.05% Tween. 100 µl of the goat anti-human IgG alkaline phosphatase conjugate diluted 1: 1000 in PBS1x / Tween 0.05%, milk 2.5%, will be incubated for 1 hour at room temperature and four washes will be carried out with PBS 1X / Tween 0.05%. The reaction will develop after 30 min incubation with 100 µL per well of para-nitrophenol phosphate substrate. The optical density will be determined at 405 nm in an ELISA reader (Dynex Technologies, INC MRX Chantilly VA). A sample is defined as positive when the optical density (OD) of the sample is three times greater than the OD of the negative control

- **Antibody functionality**. It will be evaluated by inhibition of *P. vivax* sporozoites invasion assay using Hep-G2 cells in *in-vitro* cultures. Briefly, the HepG2-A16 cell linage will be maintained in monolayer culture, washed, and resuspended in RPMI 1640 medium (Gibco, Grand Island, NY, USA) then they will be placed on Labtek plates at a concentration of 8 x 10 4 cells / 0.3 mL of Williams medium supplemented with L-glutamine, Penicillin / Streptomycin (Gibco BRL, France), Fetal Bovine serum and Dexamethasone. The cultures will be incubated overnight at 37°C in 5% CO2. Before infection, the cells will be irradiated, and 5 x 10^4 *P. vivax* sporozoites diluted in 0.1 mL of medium will be added. Then the serum of the volunteers will be added at a dilution of 1/100 in each well in duplicate; the sporozoites will invade the liver cells for five days. Subsequently, an IFAT will be performed; they will be washed with PBS and fixed with methanol. A specific HSP70 antibody will be added, and the Second fluorescent antibody Alexa 488-labeled with anti-mouse (Molecular Probes®) diluted in 1/200. The degree of maturation of the hepatic stage parasites (schizonts) will be evaluated by fluorescence. The percentage of invasion inhibition will be calculated by the formula [(Average of schizont of the negative control - an average of schizonts of the sample / Average of schizont of the negative control) x 100] (Druihle et al., 1998).

2. Evaluation of T cell response.

Cell response will be evaluated by the *in vitro* production of cytokines (cytokines IL-2, IL-4, IL-6, IL-10, TNF-α, IFN-γ in peripheral mononuclear cells (PBMC). Cells will be separated by Ficoll-Hypaque gradients from whole blood (Herrera et al., 2005) and cytokines will be measured using ELIspot and flow cytometry techniques. ELIspot. The production of IL-4 IFN-γ by total PBMC stimulated with the different antigens and/or synthetic peptides will be determined by ELIspot using commercial kits (MABTECH, Stockholm, Sweden). For IFN-γ and/or IL-4, the microplates are sensitized with 5 µg / mL of monoclonal anti-human IFN-γ antibody (1-D1K MABTECH AB, Sweden) overnight at 4°C. Subsequently, 2x105 PBMC / well is added to 1 µg / mL of the peptide (PvCS = N, R, C), using PHA as positive control and RPMI medium alone as a negative control. The plates will be incubated at 37 ° C for 40 hours in a 5% CO2 atmosphere, and washes will be carried out with PBS / 0.05% Tween-20. The anti-IFN-γ monoclonal antibody will be added and/or biotinylated IL-4 (7-B6-1, MABTECH AB, Sweden) and incubated at room temperature for 2 hours. The alkaline phosphatase with streptavidin will be added, and the reaction will be revealed by adding BCIP/NBT (5-bromo-2-chloro-3-indolyl Phosphatase/Nitroblue Tetrazolium) (Sigma, St Louis, MO). Dot-forming cells (SFCs) will be numbered using a counting system (Scanalytics, Fairfax, VA).

- **Determination of cytokines by flow cytometry.** A bead kit with anti-human cytokine antibodies will be used for the simultaneous evaluation of cytokines in the cell culture supernatant (Cytometric Bead Array - CBA) Thl/Th2 Kitt II (Becton Dickinson, Inc - BD), which allow the determination of IL- 2, IL-4, IL-6, IL-10, TNF-α, and INF-γ. PBMC will be stimulated with each of the PvCS-derived antigens at a concentration of 1 µg / mL, and cell

cultures without the peptide will be used as a negative control. In contrast, cells stimulated with phytohemagglutinin (PHA) will be included as a positive control. The supernatants will be collected on day 4 of culture and used for the determination of cytokine production. Briefly, a standard curve will be prepared according to the instructions of the commercial house. Serum or culture supernatant is added to the tubes with capture beads, and they are incubated for 30 minutes. Beads mixture, the detection reagent-PE, and the wash buffer are added for reading and analysis in the Canto II Facs cytometer.

- **Monocyte phenotyping.** PBMCs, fresh or previously cryopreserved, will be labeled with monoclonal antibodies (mAbs) specific for markers of phenotype, activation, and chemokine receptors following the manufacturer's protocols. The markers that will be used: CD14 PerCP, CD16 APC-Cy7, CD33 PE, CD56 PE Cy7, CD83 FITC, HLA-DR FITC, and CCR2 APC (BD Biosciences, San José, USA). Control isotypes will be used in all experiments. The acquisition of the cells will be made on the BD Biosciences FACSCanto II flow cytometer (Becton Dickinson, San José, USA), and the data will be analyzed using the FlowJo program (TreeStar). MO will be initially identified and selected for size and complexity. Subsequently, regions will be defined excluding NK cells (CD56hi) and dendritic cells (CD83+), but including CD14+ and CD33+ cells. OM profiles will be classified into: i) "classic OM" (CD14hi/CD16-), ii) "non-classical, pro-inflammatory OM" (CD14dim/CD16+) secreting TNFα, and iii) "anti-inflammatory OM or intermediates "(CD14hi/CD16+) with strong HLA-DR expression.

- **Phenotyping of B lymphocytes**. PBMCs, fresh or previously cryopreserved, will be labeled with specific mAbs for phenotype and activation markers following the manufacturer's protocols. The markers that will be used are CD19 PerCP, CD20 APC-Cy7, CD21 PE-Cy7, CD27 APC, CD10 PE, CD95 FITC, anti-human IgG FITC (BD Biosciences, San José, USA). Control isotypes will be used in all experiments. The cells will be acquired on the BD Biosciences FACSCanto II flow cytometer (Becton Dickinson, San José, USA) and the data will be analyzed using the FlowJo program (TreeStar). LB profiles will be classified as: i) "classical" (CD19+, CD27+, CD21+, CD10-), ii) "atypical" (CD19+, CD27-, CD21-, CD20 +, CD10-), and iii) "activated" (CD CD19+, CD27+, CD21-, CD20+, CD10-) Evaluation of the innate immune response with specific markers by flow cytometry or image cytometry

Methodology for objective # 3: *To determine the protective efficacy of the vaccine against control human malaria infection with viable P. vivax sporozoites.*

The infection clinical manifestations will be monitored by a physician, and TBS and PCR diagnosis will be made from the 7th day post-infection; once the volunteer is diagnosed positive for malaria, he/she will be treated following the MSP. The incidence of infection, the pre-patent period, the severity and frequency of symptoms will be compared between the study groups.

- **Thick blood smear** Slides will be stained with Giemsa and read independently by two experienced microscopists. Parasitaemia will be quantified by observing the microscopic fields corresponding to 300 leukocytes and the estimated count of leukocytes per µL of blood.
- **Diagnostic RT-PCR.** It will be made with genomic DNA obtained from the parasite from 500 µL blood samples from the study volunteers. Species-specific detection of *Plasmodium* will be carried out using the previously described and validated RT-PCR technique (Rougemont et al., 2004).

Methodology for objective # 4: *Evaluate the infective capacity of early P. vivax gametocyte stages in Anopheles albimanus mosquitoes.*

To evaluate the infective capacity of *P. vivax* gametocytes in Anopheles albimanus, during the infectious challenge phase (Step 3) all volunteers will be directly exposed to the bite of 20 uninfected mosquitoes from the fifth-day post-infective bite and every two days until the microscopic diagnosis is confirmed or until the 15th day as follows: on days 5, 7, 9, 11, 13 and 15) according to POE EN-02-POE-003 for a direct mosquito bite.

Mosquitoes will be placed in 7 x 7 x 15 cm "feeding cages" to be placed on the forearm or in the place of preference of each individual for 10 minutes. Mosquitoes will be evaluated for the presence of oocysts on day 7 according to POE EN-02-POE-002 and the presence of sporozoites on day 14 according to POE EN-03-POE-001.

8.2.1. Results interpretation

B cell response: Due to the low antibody titers induced by *P. vivax* under natural conditions (compared to *P. falciparum*), the responses in this study will be quantified as low, medium, and high, taking into account that any positive reaction will indicate a previous contact status with the parasite and anti-malaria immune response in the volunteers (Table 3).

Table 3. Antibody titers against *P. vivax*

Technique	**Low**	**Medium**	**Hight**
**IFAT**	<1:40	>1:40- 1:320	> 1:320
**ELISA**	1:100 -1:500	>1:1500 -1:5000	>1:5000

T-cell response. The ELIspot technique will determine cytokine production. In these techniques, the colony-forming cells (sport forming cells SFC) will be quantified, which will be

expressed as i) the average number of SFCs per 106 PMBC and will be considered significant if the average number of cells in each well with the experimental antigen is greater than the well with the control antigen ($P < 0.05$, student t-test), ii) the net number of SFCs per well (average of SFCs in the well with the experimental antigen minus the number of SFCs in the well with the antigen control) is greater than 5 SFCs per well and, iii) The average rate between the SFCs in the well with the experimental antigen and the average of SFCs in the well with the control antigen is more significant than 2.0. As for flow cytometry, the number of events to be counted in the cytometer (FACS Canto II) will be 1,800; it will run the samples from the tubes and automatically acquire the data through the software that will evaluate the data in FSC, SSC, FL1, FL2, and FL3.

MO and LB subpopulations: the percentage of specific MO and LB surface markers will be obtained and the mean fluorescence intensity of each group for classification of the populations. Average values of each of the groups will be compared with each other and during the follow-up.

9. Data handling and record-keeping

The data obtained from the study will be entered into a database designed with the REDcap program (Vanderbilt University, 2012) and stored on a server with access restricted by password. The data will be entered in the Electronic Case Report Form by the study personnel, verified by the clinical monitor according to standard operating procedures, and corrected if necessary by the authorized investigator. The verification of the data consigned in the FRC will be done, taking as reference the source documents and comparing them with the database's printed data. The electronic FRC will not be considered as a source document in any case. The Clinical Monitor will report inconsistencies to be reviewed and corrected by authorized personnel. After quality control, the information will be analyzed with Stata analysis and will be carried out using the statistical tests indicated according to the type and distribution of variables. The level of significance for the statistical tests will be $\alpha = 0.05$.
The differences between the groups when variables studied are dichotomous will be analyzed using the Chi2 test (Fisher's exact test will be used when the data number is less than 5 (comparison of continuous variables between the groups will be done using T-student test. The comparison between several groups will be done through one-way ANOVA analysis (Scheffe evaluation for post-hoc analysis).

9.1 1 Record-keeping

During the study's development, the CRFs, the participants' source documents, the informed consents, the inclusion questionnaires, and all the information pertinent to the volunteers will be kept in a safe place at the CIV. The electronic databases will be stored in non-rewritable

optical media. Participant records will be transported by authorized research personnel in a portable, safe and waterproof box (CIV Carrera 37 2Bis No.5E-08, Cali, Colombia). Once these documents have been used, they will be archived again at the CIV. At the end of the study, all reports, consent forms, questionnaires, and other pertinent records of the protocol will be archived in the VIC for 10 years, after which they will be identified as dead archives.

10. RISKS FOR THE VOLUNTEERS, THE RESEARCH GROUP AND THE ENVIRONMENT; PRECAUTIONS TO MINIMIZE THE RISK.

Using the control human malaria infection described with *P. vivax* sporozoites, our group exposed 35 healthy naïve volunteers to infection in two consecutive trials that proved to be safe with infective doses between 2-10 bites. The infections showed prepatent periods that ranged from 9-18 days with an approximate average of 11 days. The duration of symptoms was similar in all volunteers (1.5-4.5 days), and their responses to treatment were rapid and similar in all volunteers. All volunteers cleared parasitemia within the first 48 hours after treatment (Herrera, et al., 2009; Herrera, et al, 2010). In our previous studies, the prepatent period was evaluated by TBS and PCR from day 7. In some cases, the PCR detected parasitemia before the TBS but in none of the cases, the PCR detected it before 9 days. The TBS was sensitive, detecting levels of parasitemia as low as those previously described (geometric mean of 46 parasites/μL).

Likewise, with *P. falciparum,* hundreds of volunteers have been safely and reproducibly infected in the United States, 97% of these volunteers developed moderate symptoms and short duration (average duration, 3 days) (Hoffman, 1997). These volunteers could be treated without complications due to the early initiation of treatment when the parasitemia was still very low (geometric mean of 46 parasites/μL) and also because the sensitivity of the parasite to the antimalarials used was known.

A splenic rupture is an infrequent event, which has only been observed in patients with chronically established infection (Yagmur, et al., 2000). In the previous challenge study, only one of the 17 volunteers had splenomegaly as an adverse event related to the infection. (Herrera, et al., 2010) In the proposed study, the volunteers will be closely followed and treated immediately if the parasite's presence is detected.

10.1 Risks for blood donor volunteers.

Potential risks associated with donating blood may include redness, itching, infection at the puncture site, or vasovagal symptoms such as dizziness and fainting. The sample collection will be done by venipuncture under aseptic and antiseptic conditions; new and disposable sterile material will be used. A study physician will provide primary medical care to treat vasovagal episodes (lipothymia).

A short delay (10-15 minutes) in receiving the first dose of antimalarials may be associated

with donating blood; however, this risk will not significantly affect the volunteer's recovery. Every effort will be made to expedite the procedures so that antimalarial treatment can start as quickly as possible. A complete blood count will be performed to detect hematological alterations related to malaria.

There is a potential risk that an HIV-positive result in a volunteer is not appropriately handled and creates adverse effects on their personal and/or work environment. This risk will be reduced by strictly complying with the confidentiality rules. Volunteers will personally receive a copy of the results one week after the results obtention. In case of presenting a positive result for any of the infectious diseases, they will be referred to their health provider following Law 100, Article 179 of 1993 or, failing that, to the Departmental Health Secretary following Law 1543 of 1997, Chapter II of the Ministry of Health and Social Protection, to provide advice and medical care. If a volunteer already has health insurance, he or she will be referred to their private physician with the test results. These results will only be given to the volunteer.

10.2 Risks to volunteers associated with CHMI

Risks associated with CHMI include a very low risk of anaphylaxis, possible transmission of other infectious agents through mosquito bites, and risk associated with the use of antimalarial drugs.

Precautions to minimize the risk related to the malaria challenge:

- **Anaphylaxis management:** In the place where the challenge is to be carried out, medications for anaphylaxis management such as Epinephrine 1:1000, Diphenhydramine, Cimetidine, and Methyl-prednisolone will be available, which will be used by the research physician who will remain in the area of infection. An ambulance will be available and used to transport the volunteer who needs it from the Entomology Unit to IMC, a transfer that takes approximately 30 min.
- **Blood screening:** Blood from donors with *P. vivax* infection will be screened for infectious diseases as described above.

Selection of volunteers and follow-up: Volunteers will be selected if they meet each of the inclusion criteria. They will be monitored, and once the infection is documented, the volunteer will begin treatment according to the protocol. Early treatment will minimize the risk of developing severe complications, usually unusual in *P. vivax* infections. To ensure adequate follow-up of the volunteers, each of them will have all the research group data. Under the carefully controlled conditions implemented for this study, the possibility of making a late diagnosis is remote. Some transient abnormalities such as fever, headache, myalgia, nausea, vomiting, mild anemia, leukopenia, thrombocytopenia, and asthenia may occur during the infection, which is very unlikely when an early diagnosis is made. The only severe complication and direct infection of *P. vivax* in healthy adults is splenic rupture (Yagmur, et al., 2000), which is highly unlikely if the diagnosis is made as soon as parasitemia occurs, and

treatment is administered without delay. However, as a precaution, volunteers will be informed of this risk. They will be advised to avoid doing contact sports or any strenuous activity that may result in abdominal trauma, especially during the two weeks following the start of treatment.

- **Pregnancy and *Plasmodium vivax* infection:** Although the effects of *P. vivax* malaria during pregnancy are less severe than those of *P. falciparum* (Nosten, et al., 1999), *P. vivax* infection during pregnancy has been associated with high maternal parasitemia (compared to parasitemia in non-pregnant women), maternal anemia, and low birth weight (Nosten, et al., 1999; Singh, et al., 1999). Women will be guided to use contraception for at least six months after the challenge. Women participating in the study will be advised to inform their physician on time of their participation in the clinical trial in the event of pregnancy. If any of the women relapse with *P. vivax* while pregnant, they will be treated immediately, significantly reducing the risk to the mother and the fetus of having an adverse pregnancy outcome. Chloroquine is safe to be used during pregnancy (McGready, et al., 2002), as is amodiaquine to be administered as an alternative therapy. Primaquine treatment will be administered after pregnancy.
- **Relapses:** Supervised therapy with chloroquine and high doses of primaquine will be administered. No cases of relapses of *P. vivax* have been documented with the administration of a supervised combined regimen of primaquine and chloroquine (Baird, et al., 2002; Hoffman, et al., 2002). Volunteers will be followed closely after treatment.

10.3 Risks to volunteers associated with malaria treatment.

Potential side effects of antimalarial medications include nausea, vomiting, diarrhea, abdominal pain, vertigo, headache, sleep disturbances, blurred vision, itching, tinnitus, and photosensitivity. The FDA has reported the following adverse reactions in connection with the use of these medications:

- **Chloroquine phosphate**: Gastrointestinal reactions (vomiting, nausea, diarrhea, cramps), mild transient headache, hearing effects such as nerve deafness, tinnitus, and decreased hearing acuity in those with a history of it. Visual effects, dermatological reactions (pruritus and alopecia), and cardiovascular reactions (hypotension or changes in the EKG) may also occur. The use of chloroquine is contraindicated in people with psoriasis or another type of dermatological pathology.
- **Primaquine**: The most frequently encountered side effects are gastrointestinal disorders such as nausea and abdominal discomfort, especially if the drug is administered on an empty stomach. Primaquine will be administered with food intake in this study. The primaquine has been reported as the cause of leukopenia and mild methemoglobinemia is present in most individuals. The concomitant use of substances that predispose to this side effect, such as sulfonamides, should be avoided. Primaquine is not recommended in pregnant

women. The administration of a dose of 30mg per day of primaquine for more than a year in healthy adults has proven to be well tolerated as long as it is accompanied by food intake. There are no significant effects related to kidney or liver damage, evidenced by serum creatinine BUN, AST, ALT, LDH, alkaline phosphatase, and the methemoglobinemia that occurs is reversible and asymptomatic (Fryauff, et al., 1995).

- **Falcidar® (Sulfadoxine - Pyrimethamine)**: Toxic manifestations are rare and usually attributable to the sulfadoxine component. Severe skin reactions (such as erythema multiforme, Steven-Johnson syndrome, and toxic epidermal necrosis) have been reported in individuals using a weekly schedule as prophylaxis. The safety of the combination during pregnancy has not been established, but the drug has been used to treat a large number of pregnant women.

- **Amodiaquine:** The adverse reactions of amodiaquine are generally similar to those of chloroquine, the most common being nausea, vomiting, abdominal pain, diarrhea, and itching; a less common effect is bradycardia. There is evidence that itching is less common with amodiaquine than with chloroquine.

Treatment Precautions: Volunteers will receive supervised treatment, allowing close monitoring to observe side effects. The adverse events that appear will be documented as well as the potential associations with the treatment, which will receive a score of causality.
In the previous trial, the adverse events most frequently associated with treatment were gastrointestinal origin (nausea, dizziness, and epigastric pain). The symptoms reported by the volunteers did not significantly affect their daily activities.

10.4 Risk for those conducting the study

There is a low risk for the workers who are in charge of the collection and the processing of the sample of presenting accidents with the needles of the volunteers.

Personnel Precautions: To reduce risk, all workers in contact with blood or blood products should strictly follow standard precautions. Also, the blood of volunteers with HIV, hepatitis B, or Hepatitis C infection will be excluded from the study.

10.5 Risk and precautions associated with the environment.

The risk of accidental malaria transmission to someone in the community is negligible; infected mosquitoes will only be found in a restricted area of the insectary and will not be removed outside of this location at any time. Infections in volunteers will be treated quickly before

gametocytes can develop (this time is generally 10 days after the first appearance of parasites in the blood). Volunteers can only be in the area of Cali, which is not an endemic area so the natural transmission of malaria does not occur. If any of the group members are accidentally bitten by an infected mosquito or develop symptoms of malaria, they will be immediately evaluated with TBS to confirm the presence of an infection. If the result is positive, treatment with standard doses of chloroquine and primaquine will be given under supervised therapy.

11. BENEFITS

11. 1 Benefits for blood donor volunteers.

There will be no direct benefits from participating in this study. However, volunteers will receive indirect benefits such as blood tests screening for infectious diseases. In the event of a positive result for an infectious disease other than malaria, including HIV, the volunteer will be referred to the corresponding health provider according to the social health security scheme to which he/she is affiliated, with a copy of his or her results, for advice and medical assistance

11.2 Benefits for volunteers enrolled in the malaria challenge.

There are no direct benefits for volunteers participating in this study. However, volunteers may receive some indirect benefits, such as a complete blood screening that includes the screening of infectious diseases. If during the selection phase any infectious disease screening test turns out positive, including HIV, the volunteer, with a copy of the results, will be directed to the corresponding health provider according to the social health security scheme to which he/she is affiliated for obtaining counseling and medical assistance following Law 100, Article 179 of 1993 or to the Valle Health Department following Law 1543 of 1997, Chapter II of the Ministry of Health.

12. COMPENSATION

12.1 Compensation for blood donors.
Volunteers will not incur financial expenses derived from the study participation; however, as required by the Ministry of Social Protection, no monetary compensation should be made. The medical doctors evaluating volunteers will provide counseling if a pathology other than malaria is diagnosed. Transportation costs will be recognized, a complete medical evaluation will be made, and they will receive advice to find any associated pathological condition. Volunteers will be provided a snack after the blood donation.

12.2 Compensation for infectious challenge volunteers.

There will be no direct compensation derived from the participation of volunteers in this study. However, the volunteers will receive indirect benefits such as infectious disease screening and

other laboratory tests. Volunteers will not incur financial expenses derived from the study participation; therefore the costs of transportation and snacks on the day of the challenge and the days of follow-up will be covered by the study. Additionally, each time a volunteer is summoned for a procedure related to the study, the sum corresponding to one day of the current legal minimum wage will be delivered as a symbolic way of compensating the dedication of time to the development of the study.

13. CRITERIA FOR DROPPING/WITHDRAWAL OF VOLUNTEERS.

Volunteers may freely withdraw at any time during the study. If the volunteer leaves the study, he will be treated when he/she leaves or withdraws from the study using the protocols described. If a volunteer is excluded from the study for any reason, a final evaluation (physical and laboratory exams) will be performed. The reason for the withdrawal of any of the volunteers will be reported in a CRF and accompanied by supporting information.

On the other hand, regardless of the volunteer withdrawal, the CIV will provide him with timely treatment and medical care in the event of malaria and/or one of its related complications.

14. ADVERSE EVENTS.

An adverse event (AE) is considered as any sign, inconvenience, damage, dysfunction, adverse reaction to a drug, or any other undesirable result that occurs in any of the volunteers participating in the study, even those that have already been defined as expected risks. Each of these events will be reported in a CRF and given a degree of severity and causality related to the study activities (example: blood donation or malaria challenge).

The intensity of the adverse event recorded in the CRF corresponds to the highest grading during an episode. For example, if a person has a fever, the intensity is graduated according to the highest temperature recorded.

Adverse events will be divided into two groups: requested and unrequested. Requested adverse events: will be asked at every contact with the volunteers by the clinical trial staff and recorded in a CRF in the periods determined as described below.

- Local adverse events, occurring in the region of the body where the volunteers were exposed to mosquito bites. They will be verified from the moment of the challenge and up to 7 days after it.
- All local adverse events will be recorded in CRFs. Their intensity will be classified according to the following table, adapted from the document “Guidance for Industry - Toxicity Grading Scale for Healthy Adult and Adolescent Volunteers Enrolled in Preventive Vaccine Clinical Trials

(FDA, 2007) ”.

Requested local adverse events

Local Reaction	Grade 1	Grade 2	Grade 3	Grade 4
Pain	Does not interferes with the activity	Repeated use of NSAIDs > 24 hours or that interferes with the activity	Any use of opioid analgesics or that interferes with daily activity	Emergency care for > 12 h or hospitalization requirement
Sensibility	Mild discomfort to the touch	Discomfort with the movement	Significant discomfort at rest	Emergency care for > 12 h or hospitalization requirement
Erythema	2.5-5 cm	5.1-10 cm	> 10 cm	Necrosis or exfoliative dermatitis
Induration	2.5-5 cm and does not interfere with the activity	5.1-10 cm or that interferes with the activity	> 10 cm or that interferes with daily activity	Necrosis

- Systemic adverse effects will be verified from the moment of the challenge and up to 7 days after the termination of antimalarial treatment. Systemic events may be due to the body's reaction to the challenge or antimalarial drug administration. The events that occurred from the time of the challenge to the diagnosis of malaria will be attributed to *P. vivax* infection. Events occurring from the time of administration of antimalarial treatment up to 7 days after its termination will be attributed to the drug. A margin of 7 days is given after the treatment since there are subtherapeutic levels of the drug circulating during this period.
- All systemic requested adverse events will be recorded in the corresponding CRF according to the following table, adapted from the document “Guidance for Industry - Toxicity Grading Scale for Healthy Adult and Adolescent Volunteers Enrolled in Preventive Vaccine Clinical Trials (FDA, 2007)”.

Required systemic adverse events

Systemic reaction	Grade 1	Grade 2	Grade 3	Grade 4
Disease or adverse clinical event	Does not interferes with the activity	Interferes with activity but does not require medical intervention	Interferes with activity and require medical intervention	Emergency care for > 12 h or hospitalization requirement
Nausea	Does not interferes with the activity	Interferes with activity	Interferes with daily activity	Emergency care for > 12 h or hospitalization requirement

Emesis	1-2 episodes	> 2 episodes	Require LEV at home	Emergency care for > 12 h or hospitalization requirement
Diarrhea	2-3 loose stools	4-5 loose stools	6 or more loose stools or require LEV at home	Emergency care for > 12 h or hospitalization requirement
Headache	Does not interferes with the activity	Repeated use of NSAIDs > 24 hours or that interferes with the activity	Any use of opioid analgesics or that interferes with daily activity	Emergency care for > 12 h or hospitalization requirement
Fatigue	Does not interferes with the activity	Interferes with activity	Significative, interferes with daily activity	Emergency care for > 12 h or hospitalization requirement
Myalgia	Does not interferes with the activity	Interferes with activity	Significative, interferes with daily activity	Emergency care for > 12 h or hospitalization requirement

Vital signs

Vital Signs	Grade 1	Grade 2	Grade 3	Grade 4
Fieber °C	38-38.4	38.5-38.9	39-40	> 40
Tachycardia l/m	101-115	116-130	> 130	Emergency care for > 12 h or hospitalization requirement
Bradycardia l/m	50-54	45-49	< 45	Emergency care for > 12 h or hospitalization requirement
Hypertension (sistolic) mmHg	141-150	151-155	> 155	Emergency care for > 12 h or hospitalization requirement
Hypertension (diastolic) mmHg	91-95	96-100	> 100	Emergency care for > 12 h or hospitalization requirement
Hypotension (sistolic) mm Hg	85-89	80-84	< 80	Emergency care for > 12 h or hospitalization requirement
Respiratory frequency r/m	17-20	21-25	> 25	Intubation

Serum

Serum	Grade 1	Grade 2	Grade 3	Grade 4
Glucose - Hypoglycemia	65-69	55-64	45-54	< 45
Random glucose-Hyperglycemia	110-125	126-200	> 200	Requires insulin or hyperosmolar coma
BUN mg/dL	23-26	27-31	> 31	Requires dialysis
Creatinine mg/dL	1.5-1.7	1.8-2.0	2.1-2.5	Requires dialysis
ALT,AST increase in factor	1.1-2.5 x ULN	2.6-5.0 x ULN	5.1-10 x ULN	> 10 x ULN

Bilirubin - accompanied by an alteration in AST/ALT increase in factor	1.1-1.25 x ULN	1.26-1.5 x ULN	1.51-1.75 x ULN	> 1.75 x ULN
Bilirubin - without alteration in AST/ALT increase in factor	1.1-1.5 x ULN	1.6-2.0 x ULN	2.0-3.0 x ULN	> 3.0 x ULN

Hematology

Hematology	Grade 1	Grade 2	Grade 3	Grade 4
Hb women - gr/dL	11-12	9.5-10.9	8.0-9.4	< 8
Hb men - gr/dL	12.5-13.5	10.5-12.4	8.5-10.4	< 8.5
Leukocytosis - cells/mm3	10,800-15,000	15,001-20,000	20,001-25,000	> 25,000
Leukopenia - cells/mm3	2,500-3,500	1,500-2,499	1,000-1,499	< 1,000
Lymphopenia - cells/mm3	750-1000	500-749	250-499	< 205
Neutropenia - cells/mm3	1,000-1,499	500-999	499-300	< 300
Eosinophils - cells/mm3	650-1500	1501-5000	> 5000	Hypereosinophilia
Thrombocytopenia - cells/mm3	125,000-140,000	100,000-124,000	25,000-99,000	< 25,000
PT - increase in factor	1.0-1.1 x ULN	1.11-1.20 x ULN	1.21-1.25 x ULN	> 1.25 x ULN
PTT - increase in factor	1.0-1.2 x ULN	1.21-1.4 x ULN	1.41-1.5 x ULN	> 1.5 x ULN

Note: The lower cut-off point for neutrophils is set below the reference range due to the association between benign ethnic neutropenia and Afro-descendant populations.

Urine

Urine	Grade 1	Grade 2	Grade 3	Grade 4
Proteinuria	Traces	1+	2+	Hospitalization or dialysis
Glucosuria	Traces	1+	2+	Hospitalization or hyperglycemia
Hematuria (microscopic) - red blood cells/field	1-10	11-50	> 50 or macroscopic	Hospitalization or red blood cells transfusion

Non-requested adverse events: They correspond to all the adverse events presented by the volunteers that were not considered among the requested adverse events. All these events will be recorded in a CRF from the moment of the challenge until 7 days after antimalarial treatment ending. After this moment, the non-requested AEs will only be registered in the CRF corresponding to events related to treatment or that indicate malaria suspicion. In the case a volunteer visits an endemic malaria area, he/she will be subjected to blood sampling on filter paper, in addition to the routine TBS diagnosis. This sample will define a new malaria infection or a relapse due to the clinical trial.

To grade the severity of unrequested AE, the values assigned for symptoms, signs, and laboratory results in the Common Toxicity Criteria will be applied. The classification of clinical AE will be made according to the clinical judgment of the evaluating physician and the principal investigator and per the categories specified in the document "Guidance for Industry - Toxicity Grading Scale for Healthy Adult and Adolescent Volunteers Enrolled in Preventive Vaccine Clinical Trials (FDA, 2007). If there is any alteration that requires additional clinical or paraclinical studies and other controls, these will be carried out. The monitoring will be carried out until these parameters normalize. The degree of severity of the symptoms will be assigned by the doctor after evaluating the volunteer and following the definitions described below:

- Grade 1= Mild
- Grade 2= Moderate
- Grade 3= Severe
- Grade 4= Potentially life-threatening

Mild: It is a transitory, self-limited event, with the presence of minor symptoms that do not interfere with the development of the individual's everyday activities (for example, the volunteer can work or study) and does not require any medical intervention. Example: Mosquito bite site: pain and erythema; malaria: myalgias

Moderate: Events that require minimal medical intervention to improve the volunteer's condition. In these cases, once the intervention is carried out, it is expected that the individual can perform normal daily routine activities; there may be a degree of functional limitation. Example: Mosquito bite site: Itching and/or enough pain to limit movement; malaria: Fever that improves with non-steroidal anti-inflammatory drugs.

Severe: Symptoms that require treatment and prevent the individual from effectively developing daily activities. Volunteers with a severe adverse event are generally unable to work but can be safely managed as outpatients—example: malaria: flu-like reaction or fever that results in prostration.

Potential life threat: Any event that results in emergency care for a period greater than 12 hours

or requires hospitalization. Example: bronchospasm requiring parenteral medication in the emergency room or seizures assessed in the emergency room but not resulting in hospitalization.

14.1 Serious Adverse Events:

The serious adverse events will be reported following the classification of the Document of the Americas as described below:

1. results in death,
2. life-threatening requires patient hospitalization or prolongation of existing hospitalization
3. results in persistent or significant disability, or is a congenital anomaly/congenital disability

14.2 Classification of the AEs- Association to the study activities.

In agreement with the GCP norms, adverse events can occur during any interaction with the volunteer, including at the time of screening and selection and during the study processes or subsequent follow-ups. Each of the events will be classified as definitely related, probably not related, possibly related, or not related to the study activities (e.g., blood sampling, challenge, or antimalarial treatment). This classification will be made according to the medical judgment of the principal investigator and the other evaluating physicians who assess adverse events.

Degrees of Causality:

1. *Unrelated*: The event has no temporal relationship with participation in the research and is definitely related to another etiology.

2. *Probably not related:* The time of onset and the nature of the event are temporarily not related to the intervention carried out in the investigation.

3. *Possibly related:* The timing and nature of the adverse event may be a result of participation in the research, but another explanation may be more likely.

4. *Probably related:* The timing and nature of the adverse event suggest that it is related to study participation (e.g., arm erythema followed by mosquito bite). A different potential etiology is apparent but less likely.

5. *Definitely related:* Those adverse events that have a temporal relationship with the intervention under study cannot be attributed to another etiology.

The appearance of the AEs will be classified as expected or unexpected. In this study, severe or serious AEs are not expected to occur.

14.3 AEs REPORT.

Each one of the AEs presented by the volunteers related or not to the study procedures will be consigned in a CRF according to the GCP standards.

Serious AEs or those that are life-threatening for the volunteers and that are classified as possibly or probably related to participation in the study will be detailed reported electronically, by telephone, or by any other appropriate means to the ethics committee and to the clinical monitor Ricardo Palacios (Telephone: 55-11-939-40670), within the first 24 hours after its appearance, including its severity and potential impact on the other participants.

A written report will also be made, which will be sent to the entities already mentioned. This report should include the following points:

- AE report date.
- Volunteer's code.
- Date of birth, gender, and ethnicity of the volunteer.
- Name of the principal investigator.
- Study step in which the severe adverse event appeared.
- Procedures performed on the volunteer during the study and their corresponding dates.
- Date of the appearance of the serious AE.
- Full description of the serious AE.
- Signs or symptoms of the serious AE and its causality.
- Interventions carried out on the volunteer after the serious AE, including drugs administered with their doses, route of administration, and the date of the first and last dose.
- Date of the resolution of the serious AE or death.
- Consequences for the volunteer's health and on his permanence in the trial.
- Assessment and categorization of serious AE in relation to the study activities.
- Specific recommendations to guarantee the safety of volunteers, which can be translated into changes to the protocol.

The written report will be reviewed by the local clinical safety monitor and will subsequently be sent to the ethics committee's presidents; this will be done within the first three business days after the AE is presented. All the AEs and the interventions will be recorded in the file of each of the volunteers and will be included in the reports made to the ethics committees.

14.4 AEs follow-up period.

All adverse events will be followed until the outcome is classified into one of the following options:

1. Fatal
2. Unsolved
3. Resolved
4. Resolved with sequels
5. In resolution
6. Unknown

Pregnancies that have occurred in the period between the infectious challenge and 7 days after completing the antimalarial treatment will be followed up by the clinical trial group until delivery.

15. ETHIC CONSIDERATIONS

15.1 Approval of the ethics committees and organization plan.

The protocol will be submitted for review in Colombia to the Human Ethics Committees. This protocol contains the IC forms that must be signed by the participants (Annex A, B, and C) which includes the conditions on the nature and scientific integrity of the research and the information about the guarantees provided to the study volunteers. During the study, the principal investigator will be responsible for reporting on all events that may affect the safety of individuals and the continuation of the clinical trial. Recruitment activities cannot begin until the local Ethics Committees issue their approval.

15.2 Ethics committees affiliation to the United States FWA.

The CECIV is registered with the United States FWA (Federal Wide Assurance) for the protection of human subjects of the United States Department of Health and Human Services (DHHS), Office of Human Research Protection (OHRP), under the guidelines of regulation 45CFR46.103. (CECIV: FWA: FWA00016072,). The activities of these institutions with human subjects and all the activities of the Ethics Committee (IRBs) will be conducted following the dispositions of the Declaration of Helsinki. (As they have been adopted in 1996 or 2000).

15.3 Research-related injuries.

Once the volunteers are included in steps 1 and 3 of the study, they will be affiliated to the contributory regime of the General System of Social Security in Health (Health Promoting Company -EPS, for Empresa Promotora de Salud-), to a prepaid medicine service, and a life

insurance policy, these services will be provided at no cost to them and for the entire time of the study.

If the volunteer belongs to the subsidized regime, they will be transferred to the contributory regime and affiliated to the prepaid medicine service. If he/she already belongs to the contributory regime, the center will assume the payments of the volunteer and will affiliate him in the same way to the prepaid Medicine service. However, if the volunteer is a beneficiary of the “identification system for beneficiaries of social subsidies” (SISBEN), he/she may decide whether to continue in it so as not to lose the different benefits and subsidies it provides, renouncing the benefit of affiliation to the contributory regime and to prepaid Medicine, which is provided for the participation in the study. This way, the volunteer will only get the benefit of life insurance

Participants that result injured due to their cooperation with the study will get medical care at no cost to them in a health institution of level III complexity; they will not receive any other compensation derived from the injury. This situation does not eliminate the volunteer's right to seek legal assistance to which he is entitled.

16. CIV AND ASOCLINIC GOOD CLINICAL PRACTICES (GCP) AND GOOD LABORATORY PRACTICES (GLP).

The CIV located in Cali will be the place to carry out the post-challenge follow-up visits to the volunteers. The CIV was created in 2000 under the advice of the WHO (World Health Organization) as part of the TDR program (Tropical Diseases Research and Training Program) WHO/TDR and is currently developing a training program in Good Laboratory Practices (BPL) (M. Arévalo- Herrera Ph.D.). Additionally, the WHO special program for Research and Training in Tropical Diseases (TDR) has provided support and guidance for the establishment of GCPs in the CIV. The WHO/TDR chose Dr. Ricardo Palacios as the monitor of the phase I clinical trial to assess the safety and immunogenicity of the PvCS vaccine candidate for *P. vivax* in two clinical trials we developed between 2005 - 2008. Dr. Ricardo Palacios has remained linked to the CIV and later on participated as an external monitor of the two previous challenge trials (Herrera, et al, 2009; Herrera, et al, 2010). The Meridional R&D company, founded and directed by him in São Paulo (Brazil), has adapted these monitoring procedures and is authorized to act as a Contract Research Organization (CRO) by the National Council for Scientific and Technological Development (CNPq)of Brazil. During the last 5 years, most of the group members in Cali have participated in GCP workshops organized and sponsored by the NIAID in Brazil and the USA and by other agencies in Colombia. The quality controls related to the study materials will be ensured by both the clinical monitors and the CUIC.

The screening will be carried out according to the standard screening parameters implemented at the Blood Bank and to the standards required by the Ministry of Social Protection, in a clinical

laboratory duly authorized to perform these activities.

17. CONFIDENTIALITY

All information collected from volunteers will be kept strictly confidential. Each person who participates in the selection process will be assigned a 5-digit identification code. Although the names of the participants will be available in the inclusion form, this information will be kept under lock and key, as noted above. All volunteer data will be entered into the electronic database in the REDcap program. The list with the names and codes of the volunteers will have a username and password that will only be accessed by staff authorized by the Principal Investigator. In case of finding any anomaly in the results of the tests of the volunteers, they will be contacted as soon as possible to personally deliver the laboratory reports and medical recommendations. The records may be examined by monitors, auditors, and/or regulatory authorities. All individual reviews of records are bound by strict confidentiality rules.

18. RULES FOR STUDY INTERRUPTION.

This study will have a total duration of 6 months from the moment of inclusion of the volunteers. The clinical study monitor and the principal investigator will review all serious AEs according to the GCP guidelines. The occurrence of serious AEs possibly related to the study procedures under consideration by the principal investigator and the clinical monitor will lead to the suspension of the study. All review institutions and ethics committees will be informed of the development of these serious EAs by the Principal Investigator and the monitor. The CRFs will be reviewed by the clinical monitor and sent to the presidents of the IRBs within no more than three business days. The ethics committees will review the AEs and decide whether the study can continue or not. The CIV will also review the reports as well as the recommendations of the IRBs, and will make the final decision regarding the continuation of the study. The principal investigator will be informed by the CIV of the final decision

19. USE OF THE INFORMATION AND PUBLICATIONS ARISING FROM THE STUDY.

The results of this study are confidential and will be published only after the authorization of the Principal Investigator and the sponsoring institution. It is anticipated that the results of this protocol will be presented to the scientific community through oral presentations at meetings and in written publications in scientific journals. Researchers who are not named from the beginning in this protocol will need to submit to the Investigator Assurance Agreement.

20. DEVIATIONS AND MODIFICATIONS TO THE PROTOCOL.

Inadvertent non-compliance with any section of this protocol will be reported as a deviation from the protocol and will be reported to each of the monitors and the ethics committees. Any modification to the protocol will be reported and submitted to the consideration of the Ethics Committees of each of the Institutions.

21. WITHDRAWAL OF VOLUNTEERS FROM THE STUDY.

Volunteers who participate in any of the three steps of the clinical trial may withdraw at any time from the study. A memorandum for registration will be written during the study to document volunteer departures or withdrawals. There will be a CRF to report the withdrawal of volunteers.

21.1 Follow-up of volunteers who do not continue in the study.

If a volunteer is excluded from the study for any reason after the challenge but before the infection is detected, he/she will be treated immediately according to the protocol and every effort will be made to adequately monitor the volunteer.

If the volunteer presents clinical manifestations secondary to malaria, all required efforts will be made to provide the appropriate treatment and perform the pertinent follow-up evaluations up to 1 year after the challenge. The reason for the withdrawal of any volunteer will be registered in the FRC and accompanied by a memorandum supporting such information.

22. TIMETABLE

2010 2011 2012 2013 2014 2015

**ACTIVITIES**	2	4	6	8	10	12	14	16	18	20	22	24	26	28	30	32	34	36	38	40	42	44	46	48	50	52	54	56	58	60	62	64	66	68	70	72
Discussion, experimental design, and writing of study protocol								■	■	■	■	■	■																							
Antigen preparation and shipment	■	■	■	■	■	■	■	■	■	■	■	■	■	■	■	■	■	■	■	■	■	■	■													
Ethics committee review and approval (CECIV)													■	■																						
Logistics (provision of facilities, staff education, and training)					■	■	■	■	■	■	■	■	■	■	■	■	■	■	■	■	■	■	■													
INVIMA review and approval																			■	■																
Optimization of the infectious challenge model.																				■	■	■	■													
IMBANACO Ethics Committee review and approval																							■													
**Pre-selection and selection of volunteers**																											■	■	■							
**Immunizations**																														■	■		■			
**Evaluation of the immune response**																																■	■	■		
**Volunteers follow-up**																																■	■	■		
**Challenge**																																		■		
**Post-challenge follow-up**																																		■	■	■
**Data Analysis**																																			■	■
**Final report**																																				■

23. REFERENCES

Alonso P.L., Sacarlal J., Aponte J.J., Leach A., Macete E., Milman J., Mandomando I., Spiessens B., Guinovart C., Espasa M., Bassat Q., Aide P., Ofori-Anyinam O., Navia M.M., Corachan S., Ceuppens M., Dubois M.C., Demoitie M.A., Dubovsky F., Menendez C., Tornieporth N., Ballou W.R., Thompson R., Cohen J. (2004) Efficacy of the RTS,S/AS02A vaccine against *Plasmodium* falciparum infection and disease in young African children: randomised controlled trial. Lancet 364:1411-20. DOI: S0140673604172231 [pii] 10.1016/S0140-6736(04)17223-1.

Arevalo-Herrera M., Herrera S. (2001) *Plasmodium vivax* malaria vaccine development. Mol Immunol 38:443-55. DOI: S0161589001000803 [pii].

Arevalo-Herrera M., Roggero M.A., Gonzalez J.M., Vergara J., Corradin G., Lopez J.A., Herrera S. (1998) Mapping and comparison of the B-cell epitopes recognized on the *Plasmodium* vivax circumsporozoite protein by immune Colombians and immunized Aotus monkeys. Ann Trop Med Parasitol 92:539-51.

Arevalo-Herrera M., Vera O., Castellanos A., Cespedes N., Soto L., Corradin G., Herrera S. (2011a) Preclinical vaccine study of *Plasmodium vivax* circumsporozoite protein derived-synthetic polypeptides formulated in montanide ISA 720 and montanide ISA 51 adjuvants. Am J Trop Med Hyg 84:21-7. DOI: 84/2_Suppl/21 [pii]10.4269/ajtmh.2011.10-0110.

Arevalo-Herrera M., Soto L., Perlaza B.L., Cespedes N., Vera O., Lenis A.M., Bonelo A., Corradin G., Herrera S. (2011b) Antibody-mediated and cellular immune responses induced in naïve volunteers by vaccination with long synthetic peptides derived from the *Plasmodium vivax* circumsporozoite protein. Am J Trop Med Hyg 84:35-42. DOI: 84/2_Suppl/35 [pii] 10.4269/ajtmh.2011.09-0507.

Arévalo-Herrera M., Chitnis C., Herrera S. (2010) Current status of *Plasmodium vivax* vaccine. Hum Vaccin 6:124-132.

Arnot D.E., Stewart M.J., Barnwell J.W. (1990) Antigenic diversity in Thai *Plasmodium vivax* circumsporozoite proteins. Mol Biochem Parasitol 43:147-9. DOI: 0166-6851(90)90140-H [pii].

Arnot D.E., Barnwell J.W., Tam J.P., Nussenzweig V., Nussenzweig R.S., Enea V. (1985) Circumsporozoite protein of *Plasmodium vivax*: gene cloning and characterization of the immunodominant epitope. Science 230:815-8.

Barnwell J.W., Galinski M.R. (1995) *Plasmodium vivax*: a glimpse into the unique and shared biology of the merozoite. Ann Trop Med Parasitol 89:113-20.

Barnwell J.W., Galinski M.R., DeSimone S.G., Perler F., Ingravallo P. (1999) *Plasmodium vivax*, P. cynomolgi, and P. knowlesi: identification of homologue proteins associated with the surface of merozoites. Exp Parasitol 91:238-49. DOI: S0014-4894(98)94372-7 [pii] 10.1006/expr.1998.4372.

Bojang K.A., Milligan P.J., Pinder M., Vigneron L., Alloueche A., Kester K.E., Ballou W.R., Conway D.J., Reece W.H., Gothard P., Yamuah L., Delchambre M., Voss G., Greenwood B.M., Hill A., McAdam K.P., Tornieporth N., Cohen J.D., Doherty T. (2001) Efficacy of RTS,S/AS02 malaria vaccine against *Plasmodium falciparum* infection in semi-immune

adult men in The Gambia: a randomised trial. Lancet 358:1927-34. DOI: S0140-6736(01)06957-4 [pii] 10.1016/S0140-6736(01)06957-4.

Breman J.G., Egan A., Keusch G.T. (2001) The intolerable burden of malaria: a new look at the numbers. Am J Trop Med Hyg 64:iv-vii.

Burkot T.R., Wirtz R.A., Paru R., Garner P., Alpers M.P. (1992) The population dynamics in mosquitoes and humans of two *Plasmodium vivax* polymorphs distinguished by different circumsporozoite protein repeat regions. Am J Trop Med Hyg 47:778-786.

Cattani J.A., Tulloch J.L., Vrbova H., Jolley D., Gibson F.D., Moir J.S., Heywood P.F., Alpers M.P., Stevenson A., Clancy R. (1986a) The epidemiology of malaria in a population surrounding Madang, Papua New Guinea. Am J Trop Med Hyg 35:3-15.

Cattani J.A., Tulloch J.L., Vrbova H., Jolley D., Gibson F.D., Moir J.S., Heywood P.F., Alpers M.P., Stevenson A., Clancy R. (1986b) The epidemiology of malaria in a population surrounding Madang, Papua New Guinea. Am J Trop Med Hyg 35:3-15.

Cerami C., Frevert U., Sinnis P., Takacs B., Clavijo P., Santos M.J., Nussenzweig V. (1992) The basolateral domain of the hepatocyte plasma membrane bears receptors for the circumsporozoite protein of *Plasmodium falciparum* sporozoites. Cell 70:1021-33. DOI: 0092-8674(92)90251-7 [pii].

Clyde D.F. (1975) Immunization of man against *falciparum* and *vivax* malaria by use of attenuated sporozoites. Am J Trop Med Hyg 24:397-401.

Clyde D.F., McCarthy V.C., Miller R.M., Hornick R.B. (1973) Specificity of protection of man immunized against sporozoite-induced *falciparum* malaria. Am J Med Sci 266:398-403.

Cochrane A.H., Aikawa M., Jeng M., Nussenzweig R.S. (1976) Antibody-induced ultrastructural changes of malarial sporozoites. Journal of Immunology 116:859-67.

Cochrane A.H., Nardin E.H., de Arruda M., Maracic M., Clavijo P., Collins W.E., Nussenzweig R.S. (1990) Widespread reactivity of human sera with a variant repeat of the circumsporozoite protein of *Plasmodium vivax*. Am J Trop Med Hyg 43:446-51.

Collins W.E., Jeffery G.M. (1999) A retrospective examination of sporozoite- and trophozoite-induced infections with *Plasmodium falciparum* in patients previously infected with heterologous species of *Plasmodium*: effect on development of parasitologic and clinical immunity. Am J Trop Med Hyg 61:36-43.

Collins W.E., Sullivan J.S., Morris C.L., Galland G.G., Richardson B.B. (1996) Observations on the biological nature of *Plasmodium vivax* sporozoites. J Parasitol 82:216-9.

Comer R.D., Young M.D., Porter J.A., Jr., Gauld J.R., Merritt W. (1968) Chloroquine resistance in *Plasmodium falciparum* malaria on the Pacific coast of Colombia. Am J Trop Med Hyg 17:795-9.

Charoenvit Y., Collins W.E., Jones T.R., Millet P., Yuan L., Campbell G.H., Beaudoin R.L., Broderson J.R., Hoffman S.L. (1991) Inability of malaria vaccine to induce antibodies to a protective epitope within its sequence. Science 251:668-71.

Chitnis C.E. (2001) Molecular insights into receptors used by malaria parasites for erythrocyte invasion. Curr Opin Hematol 8:85-91.

Chulay J.D., Schneider I., Cosgriff T.M., Hoffman S.L., Ballou W.R., Quakyi I.A., Carter R., Trosper J.H., Hockmeyer W.T. (1986) Malaria transmitted to humans by mosquitoes infected from cultured *Plasmodium falciparum*. Am J Trop Med Hyg 35:66-8.

del Portillo H.A., Longacre S., Khouri E., David P.H. (1991) Primary structure of the merozoite surface antigen 1 of *Plasmodium vivax* reveals sequences conserved between different *Plasmodium* species. Proc Natl Acad Sci U S A 88:4030-4.

Druihle P., Reina L., Fidock D.A. (1998) Immunity to liver stages. , in: I. Sherman (Ed.), Malaria: Parasite biology, pathogenesis and protection Washington DC. pp. 513-543.

Egan J.E., Hoffman S.L., Haynes J.D., Sadoff J.C., Schneider I., Grau G.E., Hollingdale M.R., Ballou W.R., Gordon D.M. (1993) Humoral immune responses in volunteers immunized with irradiated *Plasmodium falciparum* sporozoites. Am J Trop Med Hyg 49:166-73.

Eyles D.E. (1950) A stain for malarial oocysts in temporary preparations. J Parasitol 36:501.

Fairley N.H. (1947) Sidelights on malaria in man obtained by subinoculation experiments. Trans R Soc Trop Med Hyg 40:621-76.

Franke E.D., Lucas C.M., San Roman E., Wirtz R.A. (1992a) Prevalence of antibody to the variant repeat of the circumsporozoite protein of *Plasmodium vivax* in Peru. Am J Trop Med Hyg 46:708-10.

Franke E.D., Lucas C.M., Chauca G., Wirtz R.A., Hinostroza S. (1992b) Antibody response to the circumsporozoite protein of *Plasmodium vivax* in naturally infected humans. Am J Trop Med Hyg 46:320-6.

Frevert U., Sinnis P., Cerami C., Shreffler W., Takacs B., Nussenzweig V. (1993) Malaria circumsporozoite protein binds to heparan sulfate proteoglycans associated with the surface membrane of hepatocytes. J. Exp. Med. 177:1287-1298.

Galinski M.R., Corredor-Medina C., Povoa M., Crosby J., Ingravallo P., Barnwell J.W. (1999) *Plasmodium vivax* merozoite surface protein-3 contains coiled-coil motifs in an alanine-rich central domain. Mol Biochem Parasitol 101:131-47. DOI: S0166-6851(99)00063-8 [pii].

Galinski M.R., Ingravallo P., Corredor-Medina C., Al-Khedery B., Povoa M., Barnwell J.W. (2001) *Plasmodium vivax* merozoite surface proteins-3beta and-3gamma share structural similarities with *P. vivax* merozoite surface protein-3alpha and define a new gene family. Mol Biochem Parasitol 115:41- 53. DOI: S0166-6851(01)00267-5 [pii].

Genton B., Corradin G. (2002) Malaria vaccines: from the laboratory to the field. Curr Drug Targets Immune Endocr Metabol Disord 2:255-67.

Glynn J.R., Collins W.E., Jeffery G.M., Bradley D.J. (1995) Infecting dose and severity of falciparum malaria. Trans R Soc Trop Med Hyg 89:281-3.

Gonzalez J.M., Hurtado S., Arevalo-Herrera M., Herrera S. (2001) Variants of the *Plasmodium vivax* circumsporozoite protein (VK210 and VK247) in Colombian isolates. Mem Inst Oswaldo Cruz 96:709- 12. DOI: S0074-02762001000500023 [pii].

Grassi B.A., Bignami, Bastianelli G. (1899) Ulteriori ricerche sul ciclo dei parassiti malarici umani nel corpo del zanzarone. Atti Reale Accad. Lincei 5:8-21.

Guerra C.A., Howes R.E., Patil A.P., Gething P.W., Van Boeckel T.P., Temperley W.H., Kabaria C.W., Tatem A.J., Manh B.H., Elyazar I.R., Baird J.K., Snow R.W., Hay S.I. (2010) The international limits and population at risk of *Plasmodium vivax* transmission in 2009. PLoS Negl Trop Dis 4:e774. DOI: 10.1371/journal.pntd.0000774.

Guinovart C., Aponte J.J., Sacarlal J., Aide P., Leach A., Bassat Q., Macete E., Dobano C., Lievens M., Loucq C., Ballou W.R., Cohen J., Alonso P.L. (2009) Insights into long-

lasting protection induced by RTS,S/AS02A malaria vaccine: further results from a phase IIb trial in Mozambican children. PLoS One 4:e5165. DOI: 10.1371/journal.pone.0005165.

Gunewardena D.M., Carter R., Mendis K.N. (1994) Patterns of acquired anti-malarial immunity in Sri Lanka. Mem Inst Oswaldo Cruz 89:63-5.

Gysin J., Moisson P., Pereira da Silva L., Druilhe P. (1996) Antibodies from immune African donors with a protective effect in *Plasmodium falciparum* human infection are also able to control asexual blood forms of the parasite in Saimiri monkeys. Res Immunol 147:397-401. DOI: 0923249496820487 [pii].

Herrera M.A., de Plata C., Gonzalez J.M., Corradin G., Herrera S. (1994) Immunogenicity of multiple antigen peptides containing *Plasmodium vivax* CS epitopes in BALB/c mice. Mem Inst Oswaldo Cruz 89:71-6.

Herrera S., De Plata C., Gonzalez M., Perlaza B.L., Bettens F., Corradin G., Arevalo-Herrera M. (1997) Antigenicity and immunogenicity of multiple antigen peptides (MAP) containing *P. vivax* CS epitopes in Aotus monkeys. Parasite Immunol 19:161-70.

Herrera S., Fernandez O.L., Vera O., Cardenas W., Ramirez O., Palacios R., Chen-Mok M., Corradin G., Arevalo-Herrera M. (2011a) Phase I safety and immunogenicity trial of *Plasmodium vivax* CS derived long synthetic peptides adjuvanted with montanide ISA 720 or montanide ISA 51. Am J Trop Med Hyg 84:12-20. DOI: 84/2_Suppl/12 [pii] 10.4269/ajtmh.2011.09-0516.

Herrera S., Solarte Y., Jordan-Villegas A., Echavarria J.F., Rocha L., Palacios R., Ramirez O., Velez J.D., Epstein J.E., Richie T.L., Arevalo-Herrera M. (2011b) Consistent safety and infectivity in sporozoite challenge model of *Plasmodium vivax* in malaria-naïve human volunteers. Am J Trop Med Hyg 84:4- 11. DOI: 84/2_Suppl/4 [pii] 10.4269/ajtmh.2011.09-0498.

Herrera S., Fernández O., Manzano M.R., Murrain B., Vergara J., Blanco P., Palacios R., Vélez J.D., Epstein J.E., Chen-Mok M., Reed Z.H., Arévalo-Herrera M. (2009a) Successful Sporozoite Challenge Model in Human Volunteers with *Plasmodium vivax* Strain Derived from Human Donor. Am J Trop Med Hyg Accepted.

Herrera S., Solarte Y., Parra J., Jordan A., Echavarria J.F., Rocha L., Palacios R., Ramirez O., Velez J.D., Epstein J.E., Richie T., Arevalo-Herrera M. (2009b) Reproducibility of a sporozoite challenge model for *Plasmodium vivax* in human naïve volunteers. Am J Trop Med Hyg aceptted.

Herrera S., Fernandez O., Manzano M.R., Murrain B., Vergara J., Blanco P., Palacios R., Velez J.D., Epstein J.E., Chen-Mok M., Reed Z.H., Arevalo-Herrera M. (2009c) Successful sporozoite challenge model in human volunteers with *Plasmodium vivax* strain derived from human donors. Am J Trop Med Hyg 81:740-6. DOI: 81/5/740 [pii] 10.4269/ajtmh.2009.09-0194.

Herrera S., Bonelo A., Perlaza B.L., Fernandez O.L., Victoria L., Lenis A.M., Soto L., Hurtado H., Acuna L.M., Velez J.D., Palacios R., Chen-Mok M., Corradin G., Arevalo-Herrera M. (2005) Safety and elicitation of humoral and cellular responses in colombian malaria-naïve volunteers by a *Plasmodium vivax* circumsporozoite protein-derived synthetic vaccine. Am J Trop Med Hyg 73:3-9. DOI: 73/5_suppl/3 [pii].

Herrington D.A., Nardin E.H., Losonsky G., Bathurst I.C., Barr P.J., Hollingdale M.R., Edelman R., Levine M.M. (1991) Safety and immunogenicity of a recombinant sporozoite malaria vaccine against *Plasmodium vivax*. Am J Trop Med Hyg 45:695-701.

Hoffman S.L., Doolan D.L. (2000) Malaria vaccines-targeting infected hepatocytes. Nat Med 6:1218- 9. DOI: 10.1038/81315.

Hoffman S.L., Goh L.M., Luke T.C., Schneider I., Le T.P., Doolan D.L., Sacci J., de la Vega P., Dowler M., Paul C., Gordon D.M., Stoute J.A., Church L.W., Sedegah M., Heppner D.G., Ballou W.R., Richie T.L. (2002) Protection of humans against malaria by immunization with radiation-attenuated *Plasmodium falciparum* sporozoites. J Infect Dis 185:1155-64. DOI: JID010922 [pii] 10.1086/339409.

Hurtado S., Salas M.L., Romero J.F., Zapata J.C., Ortiz H., Arevalo-Herrera M., Herrera S. (1997) Regular production of infective sporozoites of *Plasmodium falciparum* and *P. vivax* in laboratory- bred Anopheles albimanus. Ann Trop Med Parasitol 91:49-60.

Kain K.C., Brown A.E., Webster H.K., Wirtz R.A., Keystone J.S., Rodriguez M.H., Kinahan J., Rowland M., Lanar D.E. (1992) Circumsporozoite genotyping of global isolates of *Plasmodium vivax* from dried blood specimens. J Clin Microbiol 30:1863-6.

Kumar S., Epstein J.E., Richie T.L., Nkrumah F.K., Soisson L., Carucci D.J., Hoffman S.L. (2002) A multilateral effort to develop DNA vaccines against falciparum malaria. Trends Parasitol 18:129-35. DOI: S1471492201022073 [pii].

Macete E., Aponte J.J., Guinovart C., Sacarlal J., Ofori-Anyinam O., Mandomando I., Espasa M., Bevilacqua C., Leach A., Dubois M.C., Heppner D.G., Tello L., Milman J., Cohen J., Dubovsky F., Tornieporth N., Thompson R., Alonso P.L. (2007) Safety and immunogenicity of the RTS,S/AS02A candidate malaria vaccine in children aged 1-4 in Mozambique. Trop Med Int Health 12:37-46. DOI: TMI1754 [pii] 10.1111/j.1365-3156.2006.01754.x.

Machado R.L., Povoa M.M. (2000) Distribution of *Plasmodium vivax* variants (VK210, VK247 and P. vivax-like) in three endemic areas of the Amazon region of Brazil and their correlation with chloroquine treatment. Trans R Soc Trop Med Hyg 94:377-81.

Maheswary N.P., Perpanich B., Rosenberg R. (1992) Presence of antibody to a heterologous circumsporozoite protein of *Plasmodium vivax* (VK247) in southeastern Bangladesh. Trans R Soc Trop Med Hyg 86:28.

Mann V.H., Huang T., Cheng Q., Saul A. (1994) Sequence variation in the circumsporozoite protein gene of *Plasmodium vivax* appears to be regionally biased. Mol Biochem Parasitol 68:45-52. DOI: 0166-6851(94)00148-0 [pii].

Mendis K., Sina B.J., Marchesini P., Carter R. (2001) The neglected burden of *Plasmodium vivax* malaria. Am J Trop Med Hyg 64:97-106.

Miller L.H., McAuliffe F.M., Mason S.J. (1977) Erythrocyte receptors for malaria merozoites. Am J Trop Med Hyg 26:204-8.

Mourya, D. T., Gokhale, M. D., & Kumar, R. (2007). Xenodiagnosis: use of mosquitoes for the diagnosis of arboviral infections. *Journal of vector borne diseases*, *44*(4), 233.

Nardin E., Clavijo P., Mons B., van Belkum A., Ponnudurai T., Nussenzweig R.S. (1991) T cell epitopes of the circumsporozoite protein of *Plasmodium vivax*. Recognition by

lymphocytes of a sporozoite- immunized chimpanzee. J Immunol 146:1674-8.

Nussenzweig R., Vanderberg J., Most H. (1969) Protective immunity produced by the injection of x- irradiated sporozoites of *Plasmodium berghei*. IV. Dose response, specificity and humoral immunity. Mil Med 134:1176-82.

Qari S.H., Goldman I.F., Povoa M.M., di Santi S., Alpers M.P., Lal A.A. (1992) Polymorphism in the circumsporozoite protein of the human malaria parasite *Plasmodium vivax*. Mol. Biochem. Parasitol. 55:105-114.

Ramasamy R., Nagendran K., Ramasamy M.S. (1994) Antibodies to epitopes on merozoite and sporozoite surface antigens as serologic markers of malaria transmission: studies at a site in the dry zone of Sri Lanka. Am J Trop Med Hyg 50:537-47.

Richie T.L., Saul A. (2002) Progress and challenges for malaria vaccines. Nature 415:694-701. DOI: 10.1038/415694a415694a [pii].

Rieckmann K.H., Beaudoin R.L., Cassells J.S., Sell K.W. (1979) Use of attenuated sporozoites in the immunization of human volunteers against falciparum malaria. Bull World Health Organ 57:261-5.

Rodriguez M.H., Betanzos-Reyes A.F., Hernandez-Avila J.E., Mendez-Galvan J.F., Danis-Lozano R., Altamirano-Jimenez A. (2009) The participation of secondary clinical episodes in the epidemiology of *vivax* malaria during pre- and post-implementation of focal control in the state of Oaxaca, Mexico. Am J Trop Med Hyg 80:889-95. DOI: 80/6/889 [pii].

Rogers W.O., Malik A., Mellouk S., Nakamura K., Rogers M.D., Szarfman A., Gordon D.M., Nussler A.K., Aikawa M., Hoffman S.L. (1992) Characterization of *Plasmodium falciparum* sporozoite surface protein 2. Proc Natl Acad Sci U S A 89:9176-80.

Rosenberg R., Wirtz R.A., Lanar D.E., Sattabongkot J., Hall T., Waters A.P., Prasittisuk C. (1989) Circumsporozoite protein heterogeneity in the human malaria parasite *Plasmodium vivax*. Science 245:973-6.

Rougemont M., Van Saanen M., Sahli R., Hinrikson H.P., Bille J., Jaton K. (2004) Detection of four *Plasmodium* species in blood from humans by 18S rRNA gene subunit-based and species-specific real-time PCR assays. J Clin Microbiol 42:5636-43. DOI: 42/12/5636 [pii] 10.1128/JCM.42.12.5636-5643.2004.

Sachs J., Malaney P. (2002) The economic and social burden of malaria. Nature 415:680-5. DOI: 10.1038/415680a 415680a [pii].

Sattabongkot J., Tsuboi T., Zollner G.E., Sirichaisinthop J., Cui L. (2004) *Plasmodium vivax* transmission: chances for control? Trends Parasitol 20:192-8. DOI: 10.1016/j.pt.2004.02.001 S1471492204000327 [pii].

Sinnis P., Nussenzweig V. (1996) Preventing sporozoite invasion of hepatocytes, in: S. L. Hoffman (Ed.), Malaria vaccine development. A multi-immune response approach, ASM Press, Washington, D.C. pp. 15-34.

Solarte Y., Manzano M.R., Rocha L., Hurtado H., James M.A., Arévalo-Herrera M., Herrera S. (2011) *Plasmodium vivax* sporozoite production in Anopheles albimanus mosquitoes for vaccine clinical trials. The American journal of tropical medicine and hygiene 84:28-34.

Soto J., Toledo J., Gutierrez P., Luzz M., Llinas N., Cedeno N., Dunne M., Berman J. (2001) *Plasmodium vivax* clinically resistant to chloroquine in Colombia. Am J Trop Med Hyg

65:90-3.

Stoute J.A., Slaoui M., Heppner D.G., Momin P., Kester K.E., Desmons P., Wellde B.T., Garcon N., Krzych U., Marchand M. (1997) A preliminary evaluation of a recombinant circumsporozoite protein vaccine against *Plasmodium falciparum* malaria. RTS,S Malaria Vaccine Evaluation Group. N Engl J Med 336:86-91. DOI: 10.1056/NEJM199701093360202.

Stoute J.A., Heppner D.G., Jr., Mason C.J., Siangla J., Opollo M.O., Kester K.E., Vigneron L., Voss G., Walter M.J., Tornieporth N., Cohen J.D., Ballou W.R. (2006) Phase 1 safety and immunogenicity trial of malaria vaccine RTS,S/AS02A in adults in a hyperendemic region of western Kenya. Am J Trop Med Hyg 75:166-70. DOI: 75/1/166 [pii].

Stoute J.A., Kester K.E., Krzych U., Wellde B.T., Hall T., White K., Glenn G., Ockenhouse C.F., Garcon N., Schwenk R., Lanar D.E., Sun P., Momin P., Wirtz R.A., Golenda C., Slaoui M., Wortmann G., Holland C., Dowler M., Cohen J., Ballou W.R. (1998) Long-term efficacy and immune responses following immunization with the RTS,S malaria vaccine. J Infect Dis 178:1139-44.

Templeton T.J., Kaslow D.C. (1997) Cloning and cross-species comparison of the thrombospondin- related anonymous protein (TRAP) gene from *Plasmodium knowlesi, Plasmodium vivax* and *Plasmodium gallinaceum*. Mol Biochem Parasitol 84:13-24. DOI: S0166-6851(96)02775-2 [pii].

Thomas A.W., Trape J.F., Rogier C., Goncalves A., Rosario V.E., Narum D.L. (1994) High prevalence of natural antibodies against *Plasmodium falciparum* 83-kilodalton apical membrane antigen (PF83/AMA-1) as detected by capture-enzyme-linked immunosorbent assay using full-length baculovirus recombinant PF83/AMA-1. Am J Trop Med Hyg 51:730-40.

Tsuji M., Zavala F. (2001) Peptide-based subunit vaccines against pre-erythrocytic stages of malaria parasites. Mol Immunol 38:433-42. DOI: S0161589001000797 [pii].

Walther M., Dunachie S., Keating S., Vuola J.M., Berthoud T., Schmidt A., Maier C., Andrews L., Andersen R.F., Gilbert S., Poulton I., Webster D., Dubovsky F., Tierney E., Sarpotdar P., Correa S., Huntcooke A., Butcher G., Williams J., Sinden R.E., Thornton G.B., Hill A.V. (2005) Safety, immunogenicity and efficacy of a pre-erythrocytic malaria candidate vaccine, ICC-1132 formulated in Seppic ISA 720. Vaccine 23:857-64. DOI: S0264-410X(04)00642-5 [pii] 10.1016/j.vaccine.2004.08.020.

WHO. (2010) World malaria report 2010, World Health Organization, Washington.

Williams T.N., Maitland K., Bennett S., Ganczakowski M., Peto T.E., Newbold C.I., Bowden D.K., Weatherall D.J., Clegg J.B. (1996) High incidence of malaria in alpha-thalassaemic children. Nature 383:522-5. DOI: 10.1038/383522a0.

Wirtz R.A., Burkot T.R., Graves P.M., Andre R.G. (1987) Field evaluation of enzyme-linked immunosorbent assays for *Plasmodium falciparum* and *Plasmodium vivax* sporozoites in mosquitoes (Diptera: Culicidae) from Papua New Guinea. J Med Entomol 24:433-7.

Wirtz R.A., Rosenberg R., Sattabongkot J., Webster H.K. (1990) Prevalence of antibody to heterologous circumsporozoite protein of *Plasmodium vivax* in Thailand. Lancet 336:593-5. DOI: 0140-6736(90)93393-4 [pii].

Wombou Toukam CM, Solano P, Bengaly Z, Jamonneau V, Bucheton B. (2011) Experimental

evaluation of xenodiagnosis to detect trypanosomes at low parasitaemia levels in infected hosts. Parasite 18(4):295-302

Yadava A., Sattabongkot J., Washington M.A., Ware L.A., Majam V., Zheng H., Kumar S., Ockenhouse C.F. (2007) A novel chimeric *Plasmodium vivax* circumsporozoite protein induces biologically functional antibodies that recognize both VK210 and VK247 sporozoites. Infect Immun 75:1177-85. DOI: IAI.01667-06 [pii] 10.1128/IAI.01667-06.

Supplement data 1
PROCEDURE FOR THE *P. vivax* CS PROTEIN VACCINE FORMULATION

Peptides N, R and C have been packaged in 5 mL vials in quantities of 120 µg for each peptide, in two presentations:

- Mixture 1. N + C peptides (120 µg of each, total: 240 µg of protein).
- Mixture 2. N + R + C peptides (120 µg of each, total: 360 µg of protein).

Each group will receive a 50 µg dose of each of the N and C peptides, in the first injection (mix 1); and 50 µg of peptides N, R and C in the second and third injections (mix 2).

Note: Each presentation corresponds to the dose for two volunteers

First injection preparation.

For the first injection, 8 vials of 240 µg of protein will be used. Each 240 µg vial will be dissolved in 500 µL of distilled water and mixed with 500 µL of Montanide ISA-51 (Seppic, France) for a final volume of 1000 µL. Mix 20x with a 10mL glass syringe, the total (final) volume. The mixtures will be injected within the next 5 hours.

Second and third injection preparation.

For the second and third injection, 16 vials of 360 µg of protein will be used. Each 360 µg vial will be dissolved in 500 µL of distilled water and mixed with 500 µL of Montanide ISA-51 (Seppic, France) for a final volume of 1000 µL. Mix 20x with a 10mL glass syringe, the total (final) volume. The mixtures will be injected within the next 5 hours.

Montanide preparation for the control group.

500 µL of distilled water and 500 µL of Montanide ISA-51will be taken for a final volume of 1000 µL.

Each preparation will be packaged in 1 mL syringes with a 21G short needle, and 500 µL per volunteer will be injected intramuscularly into the left deltoid.

A loss of approximately 20% of each peptide is considered during this procedure. For this reason the presentation of the products is 240 and 360 µg to ensure a dose of 100 for the first injection and 150 µg for the second and third per volunteer.

Randomized clinical trial to assess the protective efficacy of a *Plasmodium vivax* CS synthetic vaccine

Supplemental Material

Protocol title

Evaluation of the protective efficacy of a synthetic vaccine derived from the

***Plasmodium vivax* CS protein**

Protocol code : 2304-493-26202

Sponsor
Departamento Administrativo de Ciencia, Tecnología e Innovación, Colciencias.

Principal Investigator: Sócrates Herrera, MD

Versión: 6.0
13 February 2015

STATEMENT OF COMPLIANCE

The study will be carried out in compliance with Good Clinical and Laboratory Practices (GCP and GLP) as required by the ISO 9000, 2001 guidelines. This study will be approved by the Institutional Review Board (IRB) (Comité de Ética Institucional del Centro Internacional de Vacunas (CECIV). This protocol contains informed consent (Supplement data 1), which includes information about the volunteers' guarantees of participating in the study.

Volunteers' recruitment and study activities will begin after approval of the protocol by the local IRBs. All aspects of the protocol involving human subject participation will be carried out under the NIAID clinical terms of awards and the ICH/GCP guidelines.

SIGNATURE PAGE

The signatures below constitute acknowledgment of the protocol and the attachments and provide the necessary assurances that this clinical study will be conducted according to all stipulations of the protocol, including all confidentiality statements and according to local legal and regulatory requirements and to the principles outlined in applicable ICH guidelines.

Principal Investigator – *Name of Site*:

Signed: ______________________________ Date: 17-02-2014

Socrates Herrera MD

TABLE OF CONTENT

STATEMENT OF COMPLIANCE 2
SIGNATURE PAGE 3
TABLE OF CONTENT 4
GLOSSARY OF ABBREVIATIONS 6
1. ROLES 8
2. 10
3. INTRODUC24
3.1 Error! Bookmark not defined.
3.2 Error! Bookmark not defined.4
3.3 LimitaError! Bookmark not defined.5
3.4 Error! Bookmark not defined.
3.4.1 Development of *P. vivax* malaria natural immunity 16
3.4.2 Identification and characterization of the CS protein 16
3.4.3 Development of the *P. vivax* CS protein as a malaria vaccine candidate 17
3.4.4 Preliminary preclinical studies in Colombia and other countries. 17
3.4.5 *P. vivax* CS protein phase I clinical trials 18
3.4.6 Infectious challenge models for *P. falciparum* and *P. vivax* 19
3.4.7 Standardization of a challenge model with *P. vivax* sporozoites 19
3.5 Scientific Justification 20
4. Error! Bookmark not defined.0
5. OBJEError! Bookmark not defined.1
5.1 General objective 21
5.2 Specific objectives Error! Bookmark not defined.1
6. Error! Bookmark not defined.2
6.1 Steps 1 and 3. Volunteer's immunization and infectious challenge Error! Bookmark not defined.2
6.2 Step 2: Donation of infected blood Error! Bookmark not defined.4
7. 255
7.1 Step 1: Volunteers selection and immunization 25
7.2 Step 2: Donation of infected blood 29
7.3 Step 3. Sporozoite obtention and infectious challenge 32
8. LABORAT390
8.1 Malaria Diagnosis: 40
8.2 Infectious diseases screening test. 40
8.2.1 Results interpretation 394
9. Error! Bookmark not defined.46
9.1 Records keeping 46
10. RISKS FOR THE VOLUNTEERS, RESEARCH GROUP AND THE ENVIRONMENT; PRECAUTIONS TO MINIMIZE RISKS 47
10.1 Risks for blood donor volunteers 47
10.2 Risks to volunteers associated with CHMI 48
10.3 Risks to volunteers associated with antimalarial treatment. 49
10.4 Risks for those conducting the study 50
10.5 Risks and precautions associated with the environment 51
11. BENEFI491
11. 1 Benefits for blood donor volunteers. 51

11.2 Benefits for volunteers enrolled in the CHMI 51
12. COMPENSATION 51
12.1 Compensation for blood donors. 51
12.2 Compensation for volunteers enrolled in the malaria challenge. 52
13. CRITERIA FOR DROPPING/WITHDRAWAL OF VOLUNTEERS. 52
14. ADVERSE EVENTS. 52
14.1 Serious adverse event: 57
14.2 Classification of AEs- Association with study activities. 57
14.3 AEs report. 58
14.4 AEs follow-up period 59
15. ETHICAL CONSIDERATIONS. 59
15.1 Approval of the ethics committees and organization plan. 59
15.2 Ethics committees affiliation to the United States FWA. 60
15.3 Research-related injuries. 60
16. CIV AND ASOCLINIC GOOD CLINICAL PRACTICES (GCP) AND GOOD LABORATORY PRACTICES (GLP). 61
17. CONFIDENTIALITY 61
18. RULES FOR STUDY INTERRUPTION. 62
19. USE OF THE INFORMATION AND PUBLICATIONS ARISING FROM THE STUDY 62
20. DEVIATIONS AND MODIFICATIONS TO THE PROTOCOL. 62
21. WITHDRAWAL OF VOLUNTEERS FROM THE STUDY. 62
21.1 Follow-up of volunteers who do not continue in the study. 63
22. TIME TABLE 64
23. REFERENCES 65

GLOSSARY OF ABBREVIATIONS

ABBREVIATIONS	DEFINITIONS
A.	*Anopheles*
AE	Adverse Event
MFA	Artificial Membrane Feeding Assay
Anti-HBc	Hepatitis B core antibodies
BUN	Blood urea nitrogen
β-HCG	Human chorionic gonadotropin-beta subunit
CAB	Center for Applied Biotechnology
CIV/MVDC	Centro Internacional de Vacunas (Malaria Vaccine Development Center)
CHMI	Controlled Human Malaria Infection
CRF	Case Report Form
GCP	Good Clinical Practices
GLP	Good Laboratory Practices
DNA	Deoxyribonucleic acid
ECG	Electrocardiogram
ELISA	Enzyme-Linked ImmunoSorbent Assay
EPS/HPC	Empresa Promotora de Salud (Health Provider Company)
FDA	Food and Drug Administration
FTA-ABS	Fluorescent-Treponemal Antibody Absorbed.
FWA	Federal Wide Assurance
G6PD	Glucose 6 Phosphate Dehydrogenase
HBsAg	Hepatitis B Surface Antigen
HBV	Hepatitis B Virus
HCV	Hepatitis C Virus
HIV	Human Immunodeficiency Virus
HTLV	Human T- lymphotropic virus
IC	Informed Consent
INS	Instituto Nacional de Salud (Colombia) - National Institutes of Health

IFAT	Immunofluorescence Antibody Test
IPS	Institución Prestadora de Servicios de Salud - Health Services Provider Institution
IRB/EC	Institutional Review Board - Ethics Committee
LDH	Lactate Dehydrogenase
NIH	National Institute of Health (US)
NIAID	National Institute of Allergy and Infectious Diseases (US
P.	*Plasmodium*
*Pv*CS	*Plasmodium vivax* circumsporozoite protein
SAE	Serious Adverse Event
SOP	Standard Operating Procedure
spp.	Species
SP	Sulfadoxine-Pyrimethamine
TBS	Thick Blood Smear
VES	Erythro-sedimentation rate
WHO	World Health Organization

3. ROLES

Principal Investigator:	Sócrates Herrera, MD.<br>Managing Director,<br>Centro Internacional de Vacunas (CIV)<br>Km 6 Vía Cali – Puerto Tejada. Corregimiento El Hormiguero<br>Telephone: (57) 317-517 0552, 2-5216228<br>sherrera@inmuno.org
Co-Investigators:	Myriam Arévalo-Herrera, PhD.<br>Scientific Director,<br>Centro Internacional de Vacunas (CIV)<br>Km 6 Vía Cali – Puerto Tejada. Corregimiento El Hormiguero<br>Telephone: (57) 317-517 0557, 2-5216228<br>marevalo@inmuno.org
	Nora Céspedes, PhD<br>Centro Internacional de Vacunas (CIV)<br>Km 6 Vía Cali – Puerto Tejada. Corregimiento El Hormiguero<br>Telephone: (57) 2-5216228<br>ncespedes@inmuno.org
	Giampietro Corradin, PhD.<br>Biochemistry Department, Lausanne University<br>155 Chemin des Boveresses<br>Epalinges, 1066<br>Switzerland<br>Giampietro.Corradin@unil.ch
	José Millán Oñate, MD. Infectologist<br>Imbanaco Medical Center<br>Carrera 38 A No. 5 A 100<br>Telephone: 57- 2 682 1000<br>Cali, Valle del Cauca<br>Colombia<br>millanonate@gmail.com
Entomology Unit:	Andrés Benito Amado, BSc.<br>Centro Internacional de Vacunas (CIV)<br>Km 6 Vía Cali – Puerto Tejada. Corregimiento El Hormiguero

	Telephone: (57) 2-5216228<br>amado@inmuno.org
Clinical Monitor:	Ricardo Palacios, MD, PhD Meridional R&D<br>Rua Fernão Dias, 128 / 34A São<br>Paulo, SP, Brazil<br>CEP 05427-000<br>Telephone: +55(11)939 40670<br>rpalacios@meridionalrd.com
Ethics Committee:	Ethics Committee, CIV (CECIV)<br>IRB# IRB00007039 - IRB00007040 FWA:<br>FWA00016072<br>Telephone: (57)-(2)-518-5677 Fax: (57)-(2)--554284<br><br>Ethics Committee, Imbanaco Medical Center (CMI) Carrera<br>38 A No. 5 A 100. Cali, Colombia.<br>Telephone: (57)-(2) - 6821000<br>Fax (57)-(2)-5186000
Data Management:	Álvaro Andrés Álvarez<br>Centro Internacional de Vacunas (CIV)<br>Km 6 Vía Cali – Puerto Tejada. Corregimiento El<br>Hormiguero<br>Telephone: 2-5216228 aalvarez@inmuno.org
Estimated Number of Admitted Volunteers	Step (2) Donation of infected blood:<br>Minimum 5, maximum 15<br>Steps (1 and 3) Immunizations and CHMI:<br>32 (plus 4-6 alternatives)
Clinical Laboratory:	ASOCLINIC Inmunología Ltda.<br>Cra 37 2 Bis 5E-08 Templete Cali, Colombia<br>Telephone: (57)-(2) - 5574929

4. PROTOCOL SUMMARY

**Title**	Evaluation of the protective efficacy of a synthetic vaccine derived from the *Plasmodium vivax* CS protein
**Name of the Product**	*Pv*CSP
**Study Objectives**	**General Objective**: To determine the protective efficacy induced by the *Pv*CS vaccine formulated in Montanide ISA-51 adjuvant, in malaria-naïve and previously exposed volunteers.<br><br>**Specific Objectives:**<br><br>5) To confirm the safety of the vaccine in naïve volunteers immunized with the *Pv*CS.<br><br>6) To determine the immunogenicity of *Pv*CS in individuals previously exposed to malaria.<br><br>7) To determine the protective efficacy of the vaccine against the infectious challenge with viable *P. vivax* sporozoites in the previous groups.<br><br>8) To evaluate the infective capacity of gametocytes in the early stages of the blood cycle of *P. vivax* in *Anopheles albimanus* mosquitoes.<br><br>.
**Study design**	Phase II a/b randomized, double-blind, controlled clinical trial, comparing two groups of naïve volunteers and volunteers previously exposed to malaria.
**Schedule**	Three intramuscular injections on days 0, 60, and 180, followed by an infectious challenge (CHMI) on day 210
**No. volunteers**	32 Volunteers

**Population**	Healthy adults of both genders, 16 naïve to malaria, 16 pre-exposed to infection

**Study Methods**	**Step 1: Volunteers selection and immunization:** 32 subjects who meet the inclusion criteria will be selected, then they will be immunized by IM route at months 0, 2, and 6, with peptides derived from the *P. vivax* Circumsporozoite Protein (*Pv*CS) (150 µg) formulated in Montanide ISA- 51. Repeated blood sampling will be done to assess safety using kidney, liver, and hematologic function tests. Adverse events (AE) will be reported and quantified immediately after each injection and at any time between the immunization and a 7-day follow-up period for each injection.<br>The vaccine immunogenicity will be evaluated by the production of antibodies to the parasite and to the peptides used in immunization, and by the induction of specific cytokines (IFN-γ, TNF-α, IL-2, IL-4, IL-6, and IL-10). Also, the populations of monocytes (MO), T lymphocytes (LT), and B lymphocytes (LB) in the study subjects will be characterized. Additionally, the antibodies' *in vitro* functionality will be evaluated through sporozoite-invasion inhibition assays to hepatocytes.<br><br>**Step 2: Donation of infected blood:** A group of parasite donor patients (n = 5-15) will be selected among *P. vivax* infected people who attend diagnostic centers located in endemic regions or Cali, with detected parasitemia ≥0.1%. They will be asked to donate a 35 mL blood sample that will be subjected to laboratory tests to rule out co-infections and will be used for the feeding and infection of *Anopheles* mosquitoes.<br><br>**Step 3. Sporozoite obtention and infectious challenge:** To evaluate the immunogen's protective-efficacy, volunteers will be challenged by the bite of 3+1 *P. vivax* sporozoites infected mosquitoes. From the 5th day after the bite, medical and parasitological monitoring will be carried out to determine the infection's appearance. All procedures in this study will be performed under GLP and GCP principles. Additionally, to evaluate the infective capacity of *P. vivax* gametocytes-infectivity during the early stages of the blood cycle, the volunteers will be exposed to the bite of 20 healthy mosquitoes every two-days from the 5th-day post-infective bite, until the moment of diagnosis or until the 15th day.

**Procedures to minimize CHMI related risks**	● Detailed analysis of the clinical history and laboratory tests to evaluate the inclusion- and exclusion-criteria of volunteers.<br>● Standard Blood Bank infectious tests on donated blood destined for mosquito infection.<br>● Closed post-CHMI hematological, parasitological, and blood chemistry follow-up will be performed at the time of diagnosis, and 28 days after the antimalarial treatment is finished; physical examination and personalized communication.<br>● Antimalarial treatment established immediately after the presence of thick blood smear (TBS) parasitemia is documented.<br>● Restriction of the entry of personnel into infected-mosquito rooms to minimize the risk of malaria transmission to the community.<br>● Immediate treatment of any individual exposed to accidental malaria infection.<br>● For the protection of health and laboratory personnel, testing of volunteers' blood samples for antibodies against HIV, hepatitis B, and hepatitis C.<br>● Standardized biosafety procedures will be followed for handling blood and body fluid samples.
**Data handling and analysis**	The data obtained from the study will be entered into an online database with the REDCap program (http://project-redcap.org/). The final processing of the data will be done using the statistical software STATA®, SPSS®, or R®.
**Study Duration**	36 months

**Facilities were the trial will be conducted**	**ASOCLINIC Inmunología**<br>Carrera 37 2Bis No. 5E-08, Cali, Colombia<br>Telephone: (57)-(2)-5574929-5574921<br>Fax: (57)-(2)-5560141<br>Clinical Laboratory<br>● Malaria diagnosis by TBS.<br>● Screening laboratory tests: blood chemistry, hematology tests, and urinalysis.<br>● IFAT *P. vivax* serology.<br><br>**CIV/MVDC**<br>Km 6 Vía Cali – Puerto Tejada. Corregimiento el Hormiguero<br>Telephone: (57) (2) 5216228/521 4060<br>Clinical Trials Unit<br>● Site for recruitment of volunteers.<br><br>**Center for Applied Biotechnology (CAB)**<br>Corregimiento el Hormiguero, Km. 6 Vía Cali- Puerto Tejada.<br>Tel: (57) (2) 521 6228/ 521 4060<br>Cali, Valle del Cauca<br>Entomology Unit:<br>● Facility for infected mosquitoes challenge<br><br>**Imbanaco Medical Center (IMC)**<br>Carrera 38 A No. 5 A 100. Cali, Colombia.<br>Telephone: (57)-(2)- 6821000<br>Fax (57)-(2)-5186000<br>● Health provider for medical care in the emergency room, hospitalization, and surgery, if required.<br>● Place where the volunteers' immunizations will take place

.

3. INTRODUCTION

Malaria produces approximately 230 million clinical cases/year worldwide, of which about 1 million results in deaths (WHO, 2010). Because of the failure of classical control measures, such as the use of insecticides and antimalarial drugs, in the last two decades, intensive work has been done to identify molecules that can be used as additional control strategies as vaccines. Considerable evidence supports their feasibility: 1) Individuals from endemic areas become clinically immune through repeated malaria exposure. 2) Passive transfer of specific antibodies or immune cells has been shown to confer protection on non-immune individuals. 3) The protective efficacy of several vaccine candidates against *P. falciparum* has recently been documented.
Although there are numerous experimental vaccines against *P. falciparum*, only a limited number of *P. vivax* antigens have been described. The most studied is the Circumsporozoite Protein (*Pv*CS) and the *Pv*s25 protein expressed in ookinetes. Our group has immunologically characterized the CS protein and has carried out both preclinical studies in primates and phase Ia clinical trials. Long synthetic peptides (LSP) derived from this protein have been used. A model for infection of human volunteers has been established using infected mosquitoes carrying mature and viable sporozoites. This infectious challenge model allows the evaluation of the protective efficacy of *P. vivax* vaccines in humans.

3.1 MALARIA EPIDEMIOLOGY

Malaria is a disease that affects approximately 230 million people. It is responsible for 1 million deaths per year, representing a substantial economic impact for populations living in developing regions, especially in sub-Saharan African countries (Breman et al., 2001; Sachs and Malaney, 2002), but also in some areas of Asia and Latin America (LA). Epidemiological indicators report that *P. vivax* infections remain widely distributed worldwide, even more than the ones due to *P. falciparum,* so that they create a significant cause of morbidity and mortality among the 2.85 billion people living at the risk of infection (Guerra et al., 2010). The majority of *P. vivax* cases are reported from Southeast Asia and the West Pacific regions and account for about ~ 70% in LA and a lesser extent (5-20%) in some African countries (Guerra et al., 2010; Mendis et al., 2001).

P. vivax malaria incidence has been increasing over the last 30 years and resistance to standard antimalarial therapy (Guerra et al., 2010). Also, in most endemic areas, both *P. falciparum* and *P. vivax* share the same vector. Therefore, *Anopheles* insecticide resistance affects the transmission and control of the two species of *Plasmodium* (Rodriguez et al., 2009). On the other hand, although *P. vivax* disease is less lethal than *P. falciparum's*, the development of silent or latent hepatic parasitic forms (hypnozoites) makes it more complicated since they constitute a reservoir that could lead to periodic reactivations (Sattabongkot et al., 2004).

3.2 VACCINES AS MALARIA CONTROL ALTERNATIVES.

Due to the flaws in classical malaria control strategies described before, vaccines are considered

a complementary strategy. Over the past two decades, ~ 30 *P. falciparum* antigens have been identified, and their immunogenicity and protective efficacy have been assessed in animals and humans (Richie and Saul, 2002). Some of these antigens are differentially expressed in sporozoites (CSP, SSP2 / TRAP) (Rogers et al., 1992), during the hepatic stage (LSA1, LSA3, EXP1), in the erythrocytic phase (MSP-1, MSP-2, AMA-1), and the sexual forms (*Pf*s25, *Pf*s45/48) among others. Most of these antigens have been identified by genomic libraries screening, by epitope-mapping using sera against complete parasites or parasite fractions as well as monoclonal antibodies (Mabs). Platforms for vaccine development encompass, among others, synthetic peptides and recombinant proteins formulated in different adjuvants, live recombinant viruses, and DNA vaccines. The immunogenicity and protective efficacy of various malaria antigens have been experimentally tested in animals and humans (Kumar et al., 2002), displaying a wide range of immunogenicity and/or protection (Genton and Corradin, 2002). Among the vaccine candidates under development, the RTS,S/ASO2A, a recombinant vaccine hybridized with hepatitis B surface antigen, has shown significant protection in clinical trials and currently represents the most advanced experimental vaccine. Phase I/IIa studies have indicated its safety immunogenicity and protective efficacy in healthy volunteers from the United States (Stoute et al., 1997). Furthermore, recent Phase IIb trials carried out in Mozambique reaffirmed this vaccine is highly immunogenic and produces protection in semi-immune individuals with a decrease in the development of severe disease in African children (Bojang et al., 2001; Macete et al., 2007; Stoute et al., 2006; Stoute et al., 1998). More recently, the same vaccine was evaluated in Phase III multicenter trials that included Burkina Faso, Ghana, Gabon, Kenya, Malawi, Mozambique, Tanzania, and other African countries (http://www.kemri-wellcome.org/projects/99). These studies and other ongoing studies have conferred great expectations about the feasibility of developing antimalarial vaccines based on subunits of the parasite (Alonso et al., 2004).

3.3 LIMITATIONS FOR *P. VIVAX* MALARIA VACCINE DEVELOPMENT.

In contrast to *P. falciparum's*, only a limited number of *P. vivax* antigens has been described, including MSP1 (del Portillo et al., 1991), AMA1 (Thomas et al., 1994), MSP3, MSP4, MSP5, RBP, and DBP on the asexual blood stages (Barnwell and Galinski, 1995; Barnwell et al., 1999; Chitnis, 2001; Galinski et al., 1999; Galinski et al., 2001; Miller et al., 1977); *Pv*s25 and *Pv*s28 on ookinetes/oocysts; and the *Pv*CS and *Pv*SSP2/TRAP antigens on the pre-erythrocytic phase (Templeton and Kaslow, 1997). Of this last phase of the cycle, only *Pv*CS has been extensively analyzed in preclinical and clinical studies (Arévalo-Herrera et al., 2010).

The limited availability of *P. vivax* antigens, in general, is explained by several factors, such as 1) the impossibility of obtaining continuous (*in vitro*) cultures of the parasite's blood forms, which would allow experimental mosquito infection; 2) difficulties for achieving a constant and adequate sporozoites production for CHMI studies; and 3) the absence of a *P. vivax* radiation attenuated sporozoites (RAS) vaccine model, which in the *P. falciparum*'s case has represented a valuable system for improving the current understanding of the immune response. Despite these hardships, our group has made progress in Colombia developing a *P. vivax* infectious challenge model and has carried out two successful studies that have demonstrated its safety and reproducibility for the evaluation of different malaria vaccine candidate's efficacy (Herrera et al., 2011b; Herrera et al., 2009c). Simultaneously, it has concentrated efforts on *Pv*CS development as a vaccine candidate, a protein orthologous to the *Pf*CS, which is a component of Pf-RTS,S. The *Pv*CS, and is the subject of the present study.

3.4 BACKGROUND AND RATIONALE

3.4.1 Development of *P. vivax malaria* natural immunity.

Like *P. falciparum*, exposure to repeated *P. vivax* infections in a short period leads to clinical immunity development. Individuals from highly endemic regions develop a moderate degree of immunity between 10 and 15 years of age, which is associated with a decrease in the appearance of the infection's clinical manifestations (Cattani et al., 1986b). In other regions where the prevalence of *P. vivax* is low, highly effective immunity is not achieved. On the other hand, although some epidemiological studies indicate that *P. vivax* infection could confer some immunity against *P. falciparum*, there is not enough evidence suggesting a cross-protection between *Plasmodium* species (Gunewardena et al., 1994; Williams et al., nineteen ninety-six). Furthermore, studies in which controlled human malaria infection has been experimentally induced have indicated that immunity is acquired in a species-specific manner (Collins and Jeffery, 1999). Because *P. vivax* biological properties differ from *P. falciparum*, it is unlikely that a heterologous vaccine will control the two parasite species. Therefore, the identification of *P. vivax* components should be used for species-specific vaccine development.

3.4.2 Identification and characterization of the CS protein

The CS proteins of *P. falciparum* and *P. vivax* were identified using sera from individuals immunized with irradiated sporozoites (Druihle et al., 1998; Hoffman and Doolan, 2000). The *P. vivax* CS was identified, and its gene was cloned in 1985 (Arnot et al., 1985). The chemical characterization of the protein indicated that its structure is similar to that of other *Plasmodium* species (Sinnis and Nussenzweig, 1996). The *P. vivax* CS protein is composed of 373 amino acids. It has a central domain (90-261 a. A.) made up of short repetitive units flanked by non-repetitive protein fragments at their amino (N) and carboxyl (C) terminals. The flanking regions contain small, highly conserved sequences called Region I (85-89aa) and Region II-plus (338-355aa). They have been identified as parasite-binding domains for the invasion of hepatocytes by sporozoites (Cerami et al. ., 1992; Frevert et al., 1993). The central domain of the protein is composed of 19 blocks of 9 amino acids each, of which two allelic forms can be found in nature, the VK210 or common type (GDRADGQPA) (Arnot et al., 1985) and the VK- 247 or variant type (ANGAGNQPG) (Tsuji and Zavala, 2001). In addition to this dimorphism, a limited polymorphism has been observed in the regions encoding the amino flanks amino (N) and carboxyl (C) of the protein. (Arnot et al., 1990; González et al., 2001; Kain et al., 1992; Machado and Povoa, 2000; Maheswary et al., 1992; Mann et al., 1994; Qari et al., 1992; Rosenberg et al., 1989; Wirtz et al., 1987). This polymorphism does not seem to significantly influence the immunogenic regions (epitopes) of the protein.

During the last two decades, different research groups, including ours, have carried out the extensive immunological characterization of this protein, using sera from individuals immunized with irradiated sporozoites and semi-immune individuals from endemic areas. These individuals antibodies recognize the CS protein and induce a precipitation reaction on the surface of living sporozoites (CSP reaction) (Cochrane et al., 1976). This leads to the neutralization of sporozoites' invasion into the hepatocytes (Nussenzweig et al., 1969). Different B epitopes have been

identified throughout the CS protein entire sequence using these sera (Arevalo-Herrera et al., 1998; Franke et al., 1992a). Sera from immune individuals from different endemic areas has recognized the VK-210 and VK-247 sequences, indicating their wide distribution (Arevalo-Herrera et al., 1998; Burkot et al., 1992; Cochrane et al., 1990; Franke et al., 1992b; Ramasamy et al., 1994; Wirtz et al., 1990). VK210 contains the AGDR sequence that is highly recognized by sera of individuals from malaria-endemic communities. Also monoclonal antibodies are capable of protecting Saimirí monkeys against challenge with infective *P. vivax* sporozoites (Charoenvit et al., 1991).

Multiple T helper cell epitopes have also been recognized in the context of Major Histocompatibility Complex (MHC) class II molecule haplotypes (Herrera et al., 1994; Nardin et al., 1991). Using nona- or deca-peptides containing binding motifs for MHC class I antigens, our group identified in the *P. vivax* CS protein five peptide sequences capable of stimulating human CD8+ lymphocytes from HLA-A*0201 individuals. These peptides induced the production of IFN-γ, a cytokine involved in protection against malaria, by mononuclear cells from individuals previously naturally infected with *P. vivax* malaria (Burkot et al., 1992; Franke et al., 1992b).

3.4.3 Development of the *P. vivax* CS protein as a malaria vaccine candidate

In 1987 the *P. vivax* CS protein was initially proposed as a vaccine candidate by R. Nussenzweig's group at New York University. It was tested as a recombinant protein (rPVCS-1) in mice that produced a strong neutralizing antibody response (Cattani et al., 1986b). Two clinical trials using recombinant proteins were later conducted but failed to induce significant immune responses, thus halting the motivation to continue the development of a CS protein-based vaccine candidate (Gunewardena et al., 1994; Williams et al., 1996); and during the next decade, no more clinical trials with this protein were reported.

3.4.4 Preliminary preclinical studies in Colombia and other countries

During the last years, the CIV has concentrated significant efforts on the *P. vivax* CS protein (described above) to obtain a rational design of the vaccine (Arevalo-Herrera and Herrera, 2001). Using the epitopes identified in the protein, a series of Multi Antigenic Peptides (MAPs) were constructed containing various B and Th epitopes. Two of the 7 MAPs designed produced strong specific antibody responses against CS and IFN-γ production. However, in preclinical trials conducted in primates, these MAPs could not be purified in the amount required for subsequent clinical trials (Herrera et al., 1997). Therefore, in the framework of cooperative studies with the group of Dr. G. Corradin from the University of Lausanne (Switzerland), we decided to use the Long Synthetic Peptides (LSP) strategy with sufficient extension (> 70-mer) to contain multiple B, Th, and CTL epitopes. LSP derived from the CS protein of *P. falciparum,* and *P. vivax* were synthesized separately and tested in preclinical trials in *Aotus* monkeys (Arevalo-Herrera et al., 1998). These studies indicated high immunogenicity and *P. vivax* sporozoites' ability to reinforce this immune response (boosting) (Herrera et al., 1997). In both tests, the animals produced high titers of specific antibodies capable of recognizing by immunofluorescence (IFAT) the native protein and stimulating the release of IFN-γ determined by the ELIspot technique.

Simultaneously with these studies in Colombia, in the United States, the Walter Reed Institute (WRAIR) group in Silver Spring (MD) developed a chimeric recombinant protein that contains sequences of different variants of *Pv*CS. The recombinant vaccine formulated in Montanide ISA

was highly immunogenic in mice, and sera recognized the CS protein from *P. vivax*-infected individuals (Yadava et al., 2007).

Our group developed a new preclinical study to test the vaccine immunogenicity in BALB/c mice and *Aotus* monkeys (Arevalo-Herrera et al., 2011a). For these studies, combinations of the three synthetic peptides corresponding to the amino (N), central (R), and carboxyl (C) regions of the CS protein were used, formulated in the adjuvants Montanide ISA 720 or Montanide ISA51. Both formulations were highly immunogenic in both species. Mice developed better antibody responses against C and R polypeptides, while N polypeptide was more immunogenic in primates. These studies stimulated progress towards the clinical development of this protein.

3.4.5 *P. vivax* CS protein phase I clinical trials

Based on these results, the CIV decided to initiate Phase I clinical studies to determine the safety, tolerability, and immunogenicity of 3 different LSP derived from the *Pv*CS protein in human volunteers. More recently, clinical studies aimed at standardizing a method to infect healthy volunteers with viable sporozoites in preparation for the development of Phase II trials to test vaccine protective efficacy. These trials were monitored by the WHO and financially supported by Colciencias, the Ministry of Social Protection, the National Institute of Health of the United States (NIH / NIAID); and are briefly described below.

First Phase Ia clinical trial

The safety, tolerability, and immunogenicity of *Pv*CS-derived LSPs were evaluated in a randomized, double-blind study. Sixty-nine healthy volunteers without exposure to malaria who met the inclusion criteria were immunized with three synthetic peptides corresponding to different regions (Amino = N; Central = R, Carboxyl = C) of the *Pv*CS protein formulated in the adjuvant Montanide ISA720 (Seppic, Inc). The three peptides administered in staggered doses of 10 µg, 30 µg, and 100 µg were safe, well-tolerated, and highly immunogenic (Herrera et al., 2005). The volunteers had minor signs and symptoms at the injection site, and none developed any serious or severe adverse event (AE). Although there were differences in the titers of antibodies against the different peptides, all the individuals were seroconverted (ELISA), and the antibodies recognized the native CS protein in sporozoites (IFAT). They demonstrated their ability to block sporozoites' invasion into viable liver cells (ISI).

Our group then subsequently performed a study on the cellular and humoral immune responses in 21 of the 69 patients in this clinical trial (Arevalo-Herrera et al., 2011b). The antibodies were predominantly IgG1 and IgG3 isotypes. They recognized some parasitic protein domains (IFAT) and partially blocked sporozoite invasion into hepatocyte lines in vitro. Most of the volunteers displayed a high antibody response, transmission-blocking activity, and the induction of IFN-γ production in vitro by the peripheral blood mononuclear cells, thus providing evidence for further studies. Therefore, the success of this first phase I clinical trial was successful prompted a new trial to optimize the vaccine formulation (Herrera et al., 2011a).

Second Phase Ia clinical trial with combined peptides

A study was designed in which the safety, tolerability, and immunogenicity of the mixture of LSP formulated in two of the most potent adjuvants available at the moment for use in humans were determined: Montanide ISA-720 and Montanide ISA-51 (Seppic, Inc) (Herrera et al.,

2011a). This clinical trial was proposed to identify which of these two adjuvants generated a better formulation to be used in subsequent Phase II trials.

For this trial, 40 healthy volunteers not previously exposed to malaria were randomized to receive three injections of placebo or the mixture of synthetic peptides N, R, and C, at doses of 50 µg or 100 µg/dose/peptide formulated in the Montanide adjuvants already described. The first immunization consisted of a mixture of the N + C peptides. The second and third immunizations were mixtures of the N + C + R peptides, and the trial was designed as a controlled, randomized, double-blind, step-dose clinical study. Once again, the vaccines were well tolerated, and there was no association with serious or severe AE.

The antibody response determined by ELISA again showed seroconversion in all individuals, but peptide N, as in the previous test, induced earlier antibodies and higher titers. Although the response against peptides C and R appeared later, 97% of the volunteers responded against these peptides after the second immunization. The confirmation of the safety, tolerability, and immunogenicity of these formulations stimulated the initiation of studies oriented to establishing an infectious challenge model with sporozoites to be used in trials of the protective efficacy of the vaccine.

3.4.6 Infectious challenge models for *P. falciparum* and *P. vivax*

The possibility of infecting human volunteers experimentally with the malaria parasite has been a practice that has been carried out for 100 years (Grassi et al., 1899) (Fairley, 1947). *P. vivax* infection was routinely done as a treatment for neuro-syphilis ("malaria-therapy") during the 1950s-60s (Glynn et al., 1995). Later, a challenge model with *P. falciparum* and *P. vivax* sporozoites was developed and used to test the protective efficacy of the radiation attenuated sporozoite vaccine (Clyde, 1975; Clyde et al., 1973; Egan et al., 1993; Herrington et al., 1991; Rieckmann et al., 1979). The *P. falciparum* challenge model has been used extensively to determine the protective efficacy of various vaccines in major research centers around the world: the United States Naval Medical Research Center (NMRC) (Rockville, MD) under the direction of Dr. S. Hoffman (Hoffman et al., 2002), the Center for Clinical Vaccinology and Tropical Medicine, Oxford University (Walther et al., 2005) and the Department of Medical Microbiology of the Nijmegen University Medical Center, The Netherlands. However, the challenge with *P. vivax* sporozoites has not been reported in the last three decades. For this reason, the CIV has invested significant effort in developing this system within biosafety control conditions, protection of volunteers, and statistical significance.

3.4.7. Standardization of a challenge model with *P. vivax* sporozoites

Due to the lack of continuous *P. vivax in-vitro* cultures, the infection procedure has been standardized using field isolates from infected patients to feed *An. Albimanus* mosquitoes adapted to laboratory conditions in the insectaries of the CIV in Cali. Using these mosquitoes, their susceptibility to experimental infection by different *Plasmodium* species was determined, achieving the establishment of optimal conditions to achieve experimental infections (Hurtado et al., 1997). In a first challenge clinical trial, 17 out of 18 volunteers were successfully infected using *P. vivax* sporozoites administered by biting batches of 2 to 10 mosquitoes. All infected volunteers developed pre-patent periods between 9-11 days, determined by the thick blood smear (TBS) and PCR. All volunteers were treated immediately after the diagnosis was confirmed. Volunteers were closely followed for early detection of AEs, which were most frequently related

to mosquito bites, were mild, and resolved within 24 and 72 hours (Herrera et al., 2009a; Herrera et al., 2009b). A second challenge clinical trial determined the reproducibility of *P. vivax* infection. In this trial, 24 healthy volunteers were randomly assigned to groups 1, 2, and 3, who were challenged with 3 ± one mosquito bites infected with three different parasites' isolates. The infection occurred this time in all volunteers with pre-patent periods similar to those of the first study, confirming the challenge system's reliability (Solarte et al., 2011). Again, the volunteers were treated as soon as the peripheral blood infection was diagnosed.

3.5 SCIENTIFIC JUSTIFICATION.

Except for the African continent, most of the world's endemic areas, including Colombia, present simultaneous transmission of both *P. falciparum* and *P. vivax*. For this reason, despite the important and valuable advances in the development of vaccines against *P. falciparum*, identification and production of antigenic components of *P. vivax* are required both for specific monovalent vaccines and for the production of multi-species vaccines. Considerable evidence indicates the feasibility of developing a vaccine against malaria. First, permanent exposure to malaria infections in endemic areas induces significant levels of immunity that prevent the development of severe and complicated malaria, and although this does not entirely prevent them against disease, it significantly decreases mortality from malaria (Cattani et al., 1986a; Gunewardena et al., 1994); Second, malaria-infected patients can be cured by passive transfer of malaria-specific immunoglobulins (Gysin et al., 1996); Third, sterile immunity can be achieved in immunized animals and humans (> 90%) with radiation-attenuated sporozoites of various species of parasites, including *P. falciparum* and *P. vivax*; Fourth, a significant number of trials conducted during the last decade have demonstrated the *P. falciparum* RTS,S vaccines protective capacity both in individuals with and without previous malaria exposure in endemic communities (Guinovart et al., 2009; Macete et al. al., 2007), thus currently representing the most advanced experimental vaccine. Fifth, although in the case of *P. vivax*, vaccine research is much more incipient, the CIV has successfully developed several clinical trials with the *Pv*CS (Herrera et al., 2011a; Herrera et al., 2005). The center has developed an infectious challenge system (Herrera et al., 2011b; Herrera et al., 2009c) that supports the proposed study's feasibility and potential success. *P. vivax* has unique biological characteristics and is known to cause infections and disease with clinical manifestations other than *P. falciparum*, among which relapses represent one of the essential aspects in the maintenance of *P. vivax* transmission (Collins et al. al., 1996). This species of the parasite develops forms that remain hibernating (hypnozoites) in the liver and is periodically reactivated, contributing to the burden of the disease and the increase in the transmission of the parasite. For this reason, inhibition of parasite development at the liver level during the asymptomatic phase of parasite development is ideal.

In this sense, the pre-clinical and Phase I clinical trials carried out in the CIV using peptides derived from the *Pv*CS that have proved to be safe, well-tolerated, and highly immunogenic fully justify their continuity towards the phase II trials proposed in this proposal.

Additionally, this assay represents a unique opportunity to evaluate *P. vivax* gametocytes' infective capacity in *Anopheles* mosquitoes by direct exposure to them to test the hypothesis that these forms of the parasite (gametocytes) develop in the early stages of the blood cycle. Even before being microscopically detectable in peripheral blood, from day five post-infection.

4. HYPOTHESIS

The application of peptides N, R, and C of *P. vivax* CS protein formulated in Montanide ISA-51 adjuvant offers protection against malaria infection in naïve individuals and previously exposed to malaria.

- Immunization with *Pv*CS protein is safe in volunteers previously exposed to malaria.
- The immunization of volunteers previously exposed to malaria with *Pv*CS protein produces a reinforcing effect on the previous immune response present in these volunteers.
- Vaccination with *Pv*CS in individuals previously exposed to malaria induces a response that may offer sterile immunity.
- *P. vivax* gametocytes develop early in the parasite's blood cycle, even before it is microscopically detectable in peripheral blood. These gametocytes are capable of infecting *Anopheles albimanus* mosquitoes.

5. OBJECTIVES

5.1 General objective

To determine the protective efficacy induced by the *Pv*CS vaccine formulated in the adjuvant Montanide ISA-51 in naïve volunteers and volunteers previously exposed to malaria.

5.2 Specific objectives

1) Confirm the safety of the vaccine in naïve volunteers immunized with the *Pv*CS.
2) To determine the immunogenicity of *Pv*CS in individuals previously exposed to malaria.
3) To determine the protective efficacy of the vaccine against the infectious challenge with viable *P. vivax* sporozoites in the previous groups.
4) To evaluate the infective capacity of gametocytes in the early stages of the blood cycle of *P. vivax* in *Anopheles albimanus* mosquitoes.

6. STUDY POPULATION

6.1 Steps 1 and 3. Volunteers immunization and infectious challenge

A total of 32 volunteers (naive and semi-immune subjects from non-endemic and malaria-endemic regions, respectively) who meet the following inclusion criteria and do not have any

exclusion criteria will be enrolled in the study.

Naïve Group

Criteria for INCLUSION of volunteers

- Healthy men and non-pregnant women between 18-45 years old.
- Freely and voluntarily sign an informed consent, accompanied by two witnesses who will also sign.
- Have no history of malaria infection.
- For women, use of an adequate contraception method from the beginning of the trial until one of the study physicians lift the contraception prescription at the end of the study.
- Accept not to travel to areas considered endemic for malaria during the infectious challenge period (1 month) (Buenaventura, Tierralta, Quibdó, Tumaco, Urabá, and Bajo Cauca).
- Be reachable by phone throughout the study period.
- Being Duffy positive (Fy +).
- Hemoglobin (Hb) levels> 11 g / dl.
- Participation availability during the period in which the study will take place.
- Not be participating in another clinical study.

Criteria for EXCLUSION of volunteers

- Age under 18 or over 45.
- Women with pregnancy confirmed by a laboratory test, lactating, or with plans to be pregnant from the moment of recruitment.
- Negative Duffy phenotype.
- G6PD deficiency.
- Any hemoglobinopathy.
- Personal history of allergies to medications or insect bites.
- History of malaria infection.
- Have received vaccination against malaria.
- Clinical or laboratory abnormalities determined by the investigator (s).
- IFAT> 1:20 for P. vivax in screening tests.
- Living in a malaria-endemic region for six months before the study.
- Clinical or laboratory evidence of systemic disease, including kidney, liver, cardiovascular, pulmonary, psychiatric, or other diseases, may negatively impact and alter study results.
- Evidence of active hepatitis B and C or HIV infection.
- History of transfusion of any blood product in the 6 (six) months before the study.
- Plan to have surgery from the recruitment period to the end of the post-challenge follow-ups.
- Presence or history of autoimmune disease (lupus, rheumatoid arthritis, thyroiditis, or other).
- Splenectomized volunteers.
- Volunteers in treatment with drugs with activity on the immune system (steroids, immunosuppressive agents, or immunomodulators). A history of alcoholism or drug abuse is defined as a habit that interferes with the individual's normal social functioning.
- Any condition that may interfere with the ability to provide free and voluntary Informed Consent.

<u>Semi-immune group</u>

Criteria for INCLUSION of volunteers

- Healthy men and non-pregnant women between 18-45 years old.
- Freely and voluntarily sign an informed consent, accompanied by two witnesses who will also sign.
- Have a history of previous malaria infection (s) and positive *P. vivax* serologic tests.
- For women, use of an adequate contraception method from the beginning of the trial until one of the study physicians lift the contraception prescription at the end of the study.
- Accept not to travel to areas considered endemic for malaria during the infectious challenge period (1 month) (Buenaventura, Tierralta, Quibdó, Tumaco, Urabá, and Bajo Cauca).
- Be reachable by phone throughout the study period.
- Participation availability during the period in which the study will take place.

Criteria for EXCLUSION of volunteers

- IFAT negative (<1:20) for *P. vivax* in screening tests.
- The other criteria applied to naïve volunteers, except the antecedent of having lived in an endemic area during the last six months.

6.2 Step 2. Donation of infected blood

A total of 5-15 *P. vivax* infected patients who seek attention in malaria diagnostic centers located in Cali, Buenaventura, or other endemic areas, with detected parasitemia ≥ 0.1% and who meet the inclusion criteria of donors of the parasite, and do not meet any exclusion criteria will be included in the study.

Inclusion criteria for blood donor volunteers

- Healthy men and non-pregnant women between 15-60 years old.
- Have a positive diagnosis of *P. vivax* malaria determined by TBS examination.
- The patient must not have non-*P .vivax* circulating malaria parasites such as *P. falciparum* or *P. malariae*.
- Have a parasitemia ≥ of 0.1% per TBS.
- Hb ≥9 gr/dL at the time of malaria diagnosis.
- The patient must have the ability to provide informed consent freely and voluntarily. If you are illiterate, you can affirm your decision to participate by putting your fingerprint on the consent form. Minors who are between 15 and 17 years old and wish to participate must sign the informed consent, and one of their parents must sign the informed consent, accompanied by two witnesses who will also sign.

EXCLUSION criteria for blood donor volunteers

- Having chronic or acute illnesses, other than *P. vivax* malaria.

Have a history of diseases or clinical conditions, which according to medical criteria may significantly increase the risk associated with participation in this study.

- Hb levels <9 g/dL at the time of recruitment.
- Have received anti-malarial treatment before diagnosis.

7. STUDY DESIGN

We propose to conduct a Phase IIa/b randomized, controlled, and double-blinded clinical trial, with 32 healthy adult men and non-pregnant women, grouped as follows: 16 volunteers with previous *P. vivax* malaria infection and 16 volunteers without malaria history (naïve). The 16-pre-immune volunteers will be selected from subjects who inhabit Colombian endemic areas, with malaria history and/or presence of anti-*P. vivax* blood-stage parasite antibodies > 1:20 by IFAT.

This study will be conducted in the CIV in Cali and will have a 36 months duration. The volunteers will be immunized at months 0, 2, and 6 with the LSP mixture (150 µg) or with placebo, formulated in the adjuvant Montanide ISA-51 (Figures 1 and 2).

The specific immune response against *P. vivax* will be evaluated in all volunteers before the first immunization and after each immunization at months 0, 1, 2, 3, 6, 7, and 8. Moreover, follow-up will be performed every 2 months until trial finalization to observe the antibody-titers evolution and their functional activity, as well as the induction of some specific cytokines like IFN- γ, TNF-α, IL-2; IL- 4, IL-6, IL-10, and to characterize the Monocytes (MO), T cells (TC) and B cells (BC). Furthermore, volunteers will be subjected to renal, hepatic, and hematologic function and pregnancy tests to determine any vaccine biological safety changes. Also, antibody functionality will be assessed *in vitro* through the Inhibition of Sporozoite Invasion (ISI) assay to Hep-G2 cells.

Thirty days after the last immunization, volunteers will be infected with *P. vivax* viable Sporozoites through infected mosquito biting (2-4 bites). The vaccine-induced protection will be determined, taking into account the prepatent period. The study will be divided into three steps as described next.

7.1 Step 1: Volunteers selection and immunization.

Recruitment

All study participants must meet the inclusion criteria and be excluded if they acquire any exclusion criteria. During the recruitment period, only the Health and Social Security General System affiliated subjects will be considered, as long as they can prove through an official document that they are affiliated in any of the system regimens. The eligible volunteers must sign an Informed Consent (IC) format after being duly informed of the study purpose. Also, a comprehension test will be applied to all volunteers to assure that they have understood the study nature, the risks and benefits related to participation and that they are signing an IC format on their free will; the evaluator will explain any element not fully understood. All IC procedures and volunteer questionnaires will be documented in the volunteer folder. Besides the IC for the vaccine trial, an IC for collecting a blood sample to perform HIV testing will be obtained. In the case the subject turns to be HIV positive, he will be informed and redirected to obtain counseling and treatment. A copy of the IC will be given to all volunteers.

Identification

A 5 characters identification code will be assigned to the study volunteers: the first character will be number 1, which corresponds to Step 1; the second and third characters will be the initials of the first name and surname followed by the number of inscription. For example, a volunteer named Mario Perdono, who is the third person recruited, will be assigned the identification code 1MP03.

Selection

The selection procedures (clinical history, physical examinations, and blood samples draw) will be carried out only after the volunteer has signed the IC. In the case, a volunteer has been selected, and the immunization program has not started within the next 12 weeks, the selection tests will be repeated. In the selection visit, the medical antecedents and the concomitant treatment will be documented by one of the clinical investigators. A complete physical examination will be carried out, including sense organs, cardiovascular (CV), pulmonary, neurological, gastrointestinal (GI), musculoskeletal and dermatologic systems. We will take urine samples and 35 ml-blood samples to perform lab-screening-tests. Should a woman be considered for the study, she will be asked to use a contraceptive method during the clinical trial. At the end of the trial, she will be subjected to a pregnancy test.

Dr. José Millán Oñate, an infectious diseases medical doctor of the CIV, will be in charge of volunteers' clinical and paraclinical assessment during recruitment and selection processes. The following volunteer screening tests will be performed at ASOCLINIC within 12 weeks before the first immunization:

Hematologic Tests:
Hemogram, G6PD determination, Duffy phenotyping, hemoglobin electrophoresis, Rh and ABO group assessment, erythrocyte sedimentation rate (ESR), and C-reactive protein (CRP)

Renal function tests:
Urinalysis, creatinine, and blood urea nitrogen (BUN)

Infectious diseases other than *P. vivax:*
HIV (two rapid tests), Hepatitis B surface antigen (HBsAg), Hepatitis C Virus (HCV), Human T Lymphotropic Virus (HTLV) 1 and 2, RPR test for syphilis, and rapid test for Chagas disease.

Confirmatory tests:
If the result of any of the HIV-rapid tests is positive (or both), a confirmatory test will be performed: Western Blot.
If RPR is positive for syphilis (at any dilution), an FTA-ABS test will be performed.
If the HBsAg is positive, the Hepatitis B Core Antibody (Anti-HBc) test will be performed.

Hepatic function tests:
ALT, AST, total bilirubin, conjugated bilirubin, PT, and PTT.

Pregnancy test:
Urine and serum β-HCG determination

Others:
Blood glucose, electrocardiogram

Immunological tests:
IFAT: Antimalaric antibodies
ANAs: Antinuclear antibodies)

Group constitution
We will recruit a total of 32 persons between 18 and 45 years of age who willingly accept to participate in the trial by signing an IC format. Two volunteers' main groups will be identified: **naïve** (group A, n = 16) and **pre-immune** (group B, n = 16); and randomly distributed in 4 subgroups as follows: two experimental subgroups (A1 and B1) of 10 subjects each, and two control subgroups (A2 and B2) of 6 subjects each. The volunteers will be stratified, as shown in Table 1.

Table 1. Group constitution. Step 1

	**Experimental**	**Control**
**Naïve (A)**	A1 (n=10)	A2 (n=6)
**Pre-immune (B)**	B1 (n=10)	B2 (n=6)
**Total**	20	12

Immunization
Subgroups A1 and B1 (n = 20) will be immunized with the vaccine, and subgroups A2 and B2 (n = 12) will be immunized with placebo (See Immunization Schedule in Figure 1). The assigned vaccine preparation will be pre-coded to minimize potential selection biases and balance potential cofounders, and code translation should remain blind to the investigator who performs the vaccination and to the clinical staff of the trial (Herrera et al., 2005). Before each immunization, each volunteer will be assessed by one of the research physicians. In case of an AE report, this will be registered in a Case Report Format (CRF). The safety and tolerability of the vaccine will be determined. Subgroups A1 and B1 will receive in the first immunization a mixture of N and C peptides (50 µg/peptide; total dose 100 µg/dose) formulated in Montanide ISA 51. For immunizations 2 and 3, the vaccine will be composed of peptides N, R, and C (50 µg peptide/dose; total 150 µg protein/dose). Volunteers of subgroups A2 and B2 will be injected with saline emulsified in the same adjuvant. This phase will last for six months.

The vaccine will be prepared as described in Supplement data 1. Using a 1 mL 25G-needle syringe, a total of 500 µL solution will be taken. The vaccine will be injected intramuscularly (IM) into the deltoid muscle of the opposite arm to that previously used for blood sampling. The infectious diseases medical doctor, Dr. José Millán Oñate, will be in charge of applying the vaccine to the volunteers during this phase.

Post-immunization follow-up

- Immediate follow-up:

Volunteers will be under direct medical observation within the next hour after immunization to detect any adverse reaction to the vaccine administration. After the 1-hour observation period, a physical examination will be performed. Eight hours post-immunization, each volunteer will receive a phone call to check his/her physical condition. Any manifestation of an AE will be reported as described in the Adverse Event section.

- Post-immunization follow-up

Personal follow-up will be carried the day after each immunization and again one or two weeks before the next one. These follow-ups will include a new clinical evaluation and if needed, an AE report. Volunteers will be provided with all the required information (including telephone numbers), so they can contact the research team members at any time. Moreover, they will be encouraged to ask questions if something is not clear.

In this study phase, the clinical and laboratory follow-up of the immunized volunteers will be in charge of the infectious diseases medical doctor.

Figure 1. Immunization Schedule

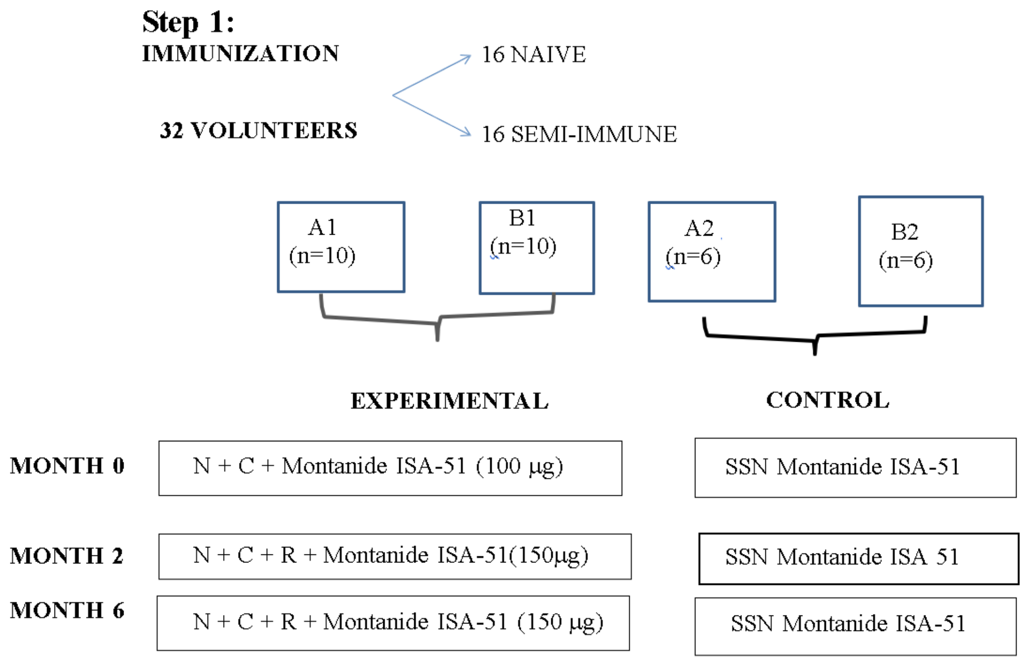

7.2 Stept 2: Donation of infected blood

In this step, *P. vivax* infected patients who attend CIV laboratories located in different endemic regions of the country or Cali (a non-endemic region) will be identified and asked to donate parasitized blood to conduct the feeding and infection of *An. albimanus* mosquitoes. Each one of the volunteers should sign an IC free and willingly. Next, 35 mL of blood from each donor will be obtained by venipuncture, which will be distributed in 5 mL that will be sent to the ASOCLINIC laboratory for the screening of infectious diseases, and a 30 mL fraction that will be used for feeding batches of adult *An. albimanus* mosquitoes.

Donors' recruitment:
The patients will be attended by a certified bacteriologist or microscopist from the CIV, who will obtain and read the TBS test. If the result is positive for *P. vivax* without other co-infecting species, and if parasitemia is ≥ 0.1%, a study's physician will explain the trial methodology and objectives and invite the patient to participate in the clinical trial. If the patient agrees to participate in the study, he or she will be asked to freely and voluntarily sign two ICs; the first will be about selection and participation in the study and the second will be for conducting HIV tests. If the patient is underage (15 to 17 years old), an explanation will be given to both the minor and the father, mother, or legal guardian so that they accept the patient's participation in the study. The minor will sign an informed assent, and the IC must be signed by a parent or legal guardian. In the end, a copy of the documents will be given to the participants.

Identification:
Volunteers will be assigned a 6-character identification code: the first character will be the number 2, which will be used only for volunteers in the second step; the second and third characters will be the initial letters of the volunteer's name and surname, respectively, followed by the registration number. For example, a volunteer named Mario Perdomo, who was the tenth person selected, will be assigned the identification code 2MP010.

Blood donation:
Once the volunteer has signed the IC and an identification code for screening has been assigned, one of the research team physicians will conduct the correspondent clinical history and physical examination. This step will result in two possible scenarios:

- The subject does not meet the inclusion criteria: In this case, the subject will be offered antimalarial treatment as indicated in the Malaria Treatment National Guidelines. The reasons why he/she is not suitable for the study and therefore cannot donate blood will be explained. Even if the subject is not included in the study, he/she will be asked to return two weeks later (Day 15 after starting the treatment) to perform a TBS that ensures malaria resolution. If the TBS is positive on day 15, the treatment schedule will be repeated. In the event of resistance to chloroquine, it will be managed as described in the section on treatment and follow-up (see below).
- The subject does meet the inclusion criteria: In this case, 35 mL of blood will be obtained by venipuncture, from which 5 mL will be used for screening of infectious diseases, and the remaining 30 mL will be used to perform the AMF in the CIV Entomology Unit.

Immediately after blood donation, volunteers will receive the medications, according to the current Malaria Treatment National Guidelines issued by the Ministry of Social Protection of Colombia.

Volunteers will be asked to come back to CIV or Buenaventura about one week later to claim their screening test results, including the HIV test. With a copy of them, the volunteer will be redirected to the correspondent health provider according to the health security regimen in which he/she is affiliated, so he/she can obtain counseling and medical assistance. If the volunteer has a private doctor, he/she will be remitted to him with a copy of all the results, and, if the subject is not affiliated to any social security regimen, he/she will be sent to one of the public health network hospitals attached to the Health Departmental Secretary.

Volunteers' treatment and follow-up:

- Chloroquine: 600mg (4 tablets of 150mg) on the first day, 450mg (three tablets) for the second, and 450mg (three tablets) on the third day.
- Primaquine: 30mg (2 tablets of 15mg) per day for 14 days.
- Follow-up: patients will be asked to return two weeks after starting the treatment (Day 15 after the first dose) to perform a TBS that ensures the cure of malaria. If the TBS is positive on day 15, the treatment will be repeated.

In case of resistance to chloroquine (*), the infection will be managed with the combination of sulfadoxine/pyrimethamine (Falcidar® 25mg pyrimethamine / 500 mg sulfadoxine) 3 single-dose tablets as an alternative treatment.

If the patient has a contraindication to Falcidar® (e.g. allergy to sulfa), he will receive amodiaquine in a dose of 3 tablets (each tablet has 200mg, dose of 600 mg/day), for three days (Bosman, et al., 2001), he will be asked to return a week later to obtain a TBS and confirm the cure.

(*) If resistance to chloroquine is confirmed, the batch of mosquitoes infected with such a sample will be discarded. If it has already been used for the challenge, the treatment of the volunteers in step 2 will be modified as described below.

Laboratory tests in donated blood

***Plasmodium* PCR:** 500 µL of the donor sample will be used to perform the PCR analysis for *P. vivax, P. falciparum,* and *P. malaria*, to rule out mixed malaria.

PCR has higher specificity and sensitivity values than any other currently available diagnostic method (WHO, 2000); and is considered the "Gold Standard" for malaria diagnosis in the research scenario. The test achieves sensitivities and specificities of up to 100% compared to other available diagnostic methods (Pöschl B., et al, 2009).

In the clinical setting, however, PCR is not the diagnostic method of choice since it is a test that requires expensive equipment and reagents, which is not readily available in many centers. Since it is a time-consuming test for its processing and reporting, there would be a delay in patient treatment.

This difficulty will not appear in this study since the diagnostic tool will be TBS, and as soon it turns out positive, the appropriate treatment will be immediately started.

The PCR to rule out infection by other *Plasmodium* species will be carried out on the donor samples after infecting mosquitoes and before using the infected batch for the challenge of healthy volunteers, to ensure that the blood has an exclusive presence of *P. vivax* parasites. In case PCR is positive for a non-*P. vivax Plasmodium* species, the batch of mosquitoes fed with this blood will be discarded according to biosecurity standards and will not be used for the challenge under any circumstance.

Although the possibility of PCR false negatives for *P. falciparum* is very low, if there is a positive donor for mixed malaria not initially diagnosed by PCR, this may be evidenced during the post-treatment follow-up since his symptoms will not improve with the *P. vivax* antimalarial treatment for this regimen is not effective against *P. falciparum*. At this moment, TBS and PRC would be performed again, and adequate treatment would be started against *P. falciparum.* The mosquitoes fed with said blood would be discarded.

Blood bank analysis: 5 ml of donor blood will be used to perform the following tests: Two rapid HIV tests from different commercial brands, antibodies against HTLV 1 and 2, hepatitis B surface antigen (HBsAg), rapid-test for Hepatitis C, rapid-test for Chagas disease and RPR for syphilis.

Confirmatory tests: If any of the HIV rapid tests turn positive, Western Blot will be performed as a confirmatory test. If the HBsAg is positive, anti-core hepatitis B antibodies will be assessed to confirm the diagnosis. Likewise, if the RPR for syphilis is reported positive at any dilution, an FTA-ABS test will be conducted.

Although it is theoretically possible that mosquitoes can transmit the Hepatitis B virus within 72 hours after being fed with blood (Blow, et al., 2002), no evidence has been found that any of these diseases are transmitted by *Anopheles* mosquitoes. Besides, mosquitoes have an incubation period of 15 to 18 days, during which the transmission of any of these diseases is unlikely.

Additional Tests: In addition to routine blood bank examinations, other pathogens that could be inadvertently transmitted by *Anopheles* mosquitoes during the challenge will be considered.

Discussions held before challenge trial # 1 (Herrera, et al, 2009) with experts in vector-borne

diseases indicated the following.

Filarias: Dr. Augusto Corredor, former director of the Parasitology Unit of the National Institute of Health of Colombia, and Dr. David Botero (parasitologist), professor at the Antioquia University (Medellín, Colombia) confirmed that there is no evidence of current transmission of no filarial species in Colombia and that assessment for these parasites is not necessary. These concepts were confirmed at the time with Dr. Dwayne Grubman, chief of the Section on Diseases caused by Filaria at the Centers for Disease Control (CDC) of the United States.

Other parasites: Although *Leishmania spp* is endemic to this region, they are not transmitted by *Anopheles* mosquitoes.

Viruses: Likewise, before the previous challenge trial, Dr. Jorge Boshell (virologist), former director of the National Institute of Health of Colombia, and Dr. Robert B. Tesh (MD), Professor of the Department of Pathology of the Center for Tropical Diseases at Texas University (Galveston, Texas), were consulted to determine the possible need to evaluate donated blood against viruses other than those studied in the Blood Bank tests, both consultants considered it is not necessary.

Additionally, the 1-3 year follow-up of the volunteers who were enrolled in the previous trials of our group (Herrera, et al, 2009; Herrera, et al, 2011), did not refer to any type of pathology associated with the experimental infection.

7.3 Step 3. Sporozoite obtention and infectious challenge.

Mosquito infection

Female *An. Albimanus* mosquitoes will be prepared in compliance with GLP conditions in any of the CIV insectaries located in different malaria-endemic regions or Caucaseco (Cali). The blood samples collected in step 2 will be tested to confirm the presence of *P. vivax* parasites by TBS microscopic examination, peripheral blood smear, and RT-PCR. Parasitaemia will be determined by TBS after reading 300 microscopic fields by an independently-well-trained microscopist. All selected samples should have a parasitemia above> 0.1%. Whereas RT-PCR allows precise identification of the parasite species and greater sensitivity (8-10 parasites/µl), TBS allows total quantification of parasitemia and gametocytaemia.

Blood samples will be used to infect an equal number of mosquito batches containing 10,000 mosquitoes per batch. Blood samples will be centrifuged at 3000 rpm for 5 min. at room temperature and the autologous plasma will be removed. The blood will be washed with RPMI1640 medium and will be reconstituted at 50% hematocrit with equal volumes of 1 pool of AB non-immune human serum obtained from a blood bank. The complement of serum AB will be inactivated by heating at 56ºC for 30 min. After washing the blood, 3-4 day old female mosquitoes

that have been fasted overnight will be fed using an artificial membrane feeder apparatus at 37°C, as previously described (Hurtado et al., 1997). Mosquito boxes will be labeled with a feeding code and the date of infection. The day after feeding, the females that do not have blood will be removed from the box, and the fed mosquitoes will be kept under strict biosecurity norms under conditions of temperature and humidity described in the respective Standard Operating Procedures (SOP) for the optimal functioning of the mosquito colony.

The mosquito batches fed with blood samples confirmed with *Plasmodium* coinfection (e.g. *P. falciparum*) will be discarded under biosafety conditions. In contrast, the batches fed with samples containing exclusively *P. vivax* will be kept. Samples from these fed mosquitoes will be dissected on days 7 and 8 after feeding to determine the presence of oocysts in their midgut, and on days 14 and 15 to assess the sporozoite load in their salivary glands. For oocyst analysis, 40 mosquitoes will be dissected and their midguts will be stained with 2% mercurochrome and examined microscopically, as described by Eyles (Eyles, 1950). Oocyst infection will be calculated according to the equation Nx79 / N + 79, where N is the number of live mosquitoes on the day of mosquito dissection. The results will be expressed as the percentage of infected mosquitoes and the arithmetic mean of the number of oocysts per intestine of dissected mosquitoes. Batches of positive mosquitoes will be kept inside the insectarium's biosecurity room, where they will be kept for another seven days until they are examined for the presence of sporozoites. The dissected salivary glands (6 lobes) will be mounted on a non-slip slide in a drop of PBS (Phosphate Buffer Saline) and will be crushed by the application of pressure. The salivary glands will be examined microscopically with a resolution of 400x magnification to evaluate the density of sporozoites for each pair of glands. Each preparation will be classified as 1+ (1-10 spz), 2+ (11-100 spz), 3+ (101-1000 spz) or 4+ (> 1001 spz) (Chulay et al., 1986). A total of 38 mosquitoes from each batch will be dissected and microscopically examined on day 14 to determine the presence of sporozoites in the mosquito's salivary gland.

Challenge batch exclusion criteria

- Batches infected with blood from volunteers infected with mixed malaria (*Pv* + *Pf*), HIV, HTVL-1/-2, Hepatitis B, Hepatitis C, Chagas, syphilis, and any other criteria determined by the researcher, such as the occurrence of therapeutic failure in the donor suggesting Chloroquine resistance of the *P. vivax* strain.
- Batches with a percentage of mosquitoes infected with < 50% sporozoites.

Challenge preparation

Volunteers will be invited to visit the Entomology Unit two (2) days before the challenge. This visit will allow them to become familiar with the place where it will take place, which will reduce the anxiety of the challenge day. At this moment, all the volunteers will be taken 35 ml of blood to assess their baseline immune status.

The female volunteers who participate in the study will be warned to immediately contact one of the study doctors if they become pregnant in the period between the selection visit and the challenge moment. One day before the challenge, the women will be cited in the CIV facilities to perform a blood pregnancy test. If any of them are positive, they will be immediately excluded from the study. If pregnancy occurs, the volunteer will be replaced by one of the alternate volunteers.

On the challenge day, the volunteers will be evaluated by one of the study doctors, who will perform a clinical history and a short physical examination, including vital signs. If it is found that any of the volunteers have an acute illness, which in the opinion of the evaluator requires the exclusion of the volunteer, he/she will be replaced by one of the alternate volunteers.

Mosquitoes from the chosen batches for the challenge will be distributed in small “feeding cages” of 7x7x7 cm. Before the challenge, the needed cages will be prepared using mosquitoes from the same batch, with three mosquitoes in each.

Controlled Human Malaria Infection with *P. vivax* infected mosquitoes

According to the previously established procedure, the 32 volunteers immunized in step 1 will be challenged on day ~ 210 of the study, one month after the third immunization, by exposure to the bites of 2-4 infected mosquitoes. The procedure will be carried out in a security room within the CAB Entomology Unit.

The "feeding cages" will be placed on the volunteer's forearm for 10 minutes, allowing the feeding window that is covered by a mesh to be against the surface of the volunteer's skin.

After feeding, the volunteers will remain in the insectary. Simultaneously, the Entomology Unit technicians will observe the number of mosquitoes fed and determine the number of infected mosquitoes per cage by dissection of the salivary glands and microscopic examination to quantify the sporozoites load in the vector’s glands. If the bite rate (determined by the presence or absence of blood-feeding) and the infectivity rate (determined by the sporozoite load in the salivary gland dissection) is below the minimum number of infective bites predicted in any of the volunteers, other mosquitoes will be employed, until a total of 2-4 mosquitoes are fed and infected with sporozoites located in the salivary glands. For example, if only two mosquitoes were fed on a volunteer and the remaining mosquitoes were positive for sporozoites, only one mosquito would be placed in the next exposure. With another volunteer, it could be found that only one mosquito was infected; in the following exhibition, two mosquitoes will be placed.

The volunteers will be observed directly by one of the study medical doctors for one hour after

the challenge, allowing them to detect any adverse reaction induced by the mosquito bite immediately. Approximately eight hours after the challenge, the volunteers will be checked by phone to document their progress. Volunteers will have all the information they need to contact researchers 24 hours a day (including cell phone number) and be encouraged to ask if they have questions or require any guidance. This direct contact will take place for three weeks. The clinical and paraclinical monitoring of the volunteers in this phase will be in charge of Dr. José Millán Oñate, the study's infectious diseases specialist.

Xenodiagnosis and evaluation of gametocyte infectivity:

Plasmodium vivax gametocytes' infective capacity in *Anopheles* mosquitoes will be studied by direct exposure of the vectors to them to test the hypothesis that these forms of the parasite (gametocytes) develop in the early stages of the blood cycle, even before being microscopically detectable in peripheral blood five days after the infection. For this process, direct infection and artificial membrane feeding will be compared. Mosquitoes will be used since xenodiagnoses have a greater capacity to detect the parasite than laboratory diagnostic tests, as has already been reported in other communicable diseases such as Chagas disease, trypanosomiasis, leishmaniasis, and Arbovirus infections (Mourya, Gokhale, et al. 2007; Wombou Toukam, Solano, et al. 2011). Volunteers will be exposed to the bite of 20 uninfected *An. Albimanus* mosquitoes by direct exposure according to POE EN-02-POE-003 on a procedure for a direct mosquito bite. Mosquitoes will be placed in 7 x 7 x 15 cm “feeding cages” to be placed on the forearm or in the place of preference of each individual for 10 minutes. Mosquitoes will be evaluated for the presence of oocysts on day 7 according to POE EN-02-POE-002 and the presence of sporozoites on day 14 according to POE EN-03-POE-001. Exposure to mosquitoes will be done every two days from the fifth day until the microscopic diagnosis is confirmed or until day 15 as follows: on days 5, 7, 9, 11, 13, and 15.

Post-Challenge Evaluation

Monitoring of the pre-patent parasitemia period: from day one to day six post-challenge, the volunteers will be followed by telephone by the study staff. Volunteers will receive instructions about malaria symptoms such as fever, headache, chills, and myalgia, malaise, which can occur between days 7 and 23 after the challenge. Although parasitemia is unlikely to appear before the ninth day, the study team will be available to care for any volunteer who exhibits early symptoms of malaria.

After infection, the volunteers will remain under medical observation for an hour, during which they will be strictly monitored. In contrast, the feeding and infection of the mosquitoes are confirmed, and it is determined that no adverse reactions will occur. After this period, the volunteers will leave the Center, and ~ 8 hours later, they will be checked to document their evolution. Subsequently, a personal control will be carried out at 24 hours, and from then on, a

daily telephone follow-up will continue until day seven post-challenge. From day seven post-challenge and until day 28, the volunteers will be daily evaluated by a study medical doctor, followed up every day with TBS, and a blood sample will be taken for the subsequent performance of RT-PCR with comparative purposes at the end of the study. If the volunteer has a fever (axillary temperature > 38ºC) and/or other signs/symptoms of malaria, TBS and peripheral blood smears will be done twice a day. If a volunteer presents symptoms compatible with malaria but his thick film is negative on 3 successive occasions, a diagnostic test for malaria by RT-PCR will be performed immediately (Rougemont et al., 2004) to confirm the diagnosis. However, the gold standard for treatment should be the thick smear. If any of the volunteers who developed the infection needs to be hospitalized, they will be treated at the Imbanaco Medical Center by the infectious disease specialist who is a member of the clinical team.

If 28 days after the challenge, the volunteers are negative for malaria, they will continue with their parasitological monitoring (TBS) twice a week until day 60 after the challenge. During this period, volunteers will be daily monitored by phone. Some volunteers are expected to develop sterile immunity; however, some may be only partially protected and develop more extended pre-patent periods. Those individuals who become infected and present pre-patent periods similar to those of group C, that is, their pre-patent periods do not have a statistically significant difference from that of unvaccinated controls will be considered unprotected. If any of the volunteers develop malaria in this phase, the study's physician will be in charge of administering and monitoring the treatment.

Malaria Treatment

Once the malaria infection is demonstrated, the volunteers will be treated with the antimalarial regimen recommended by the Ministry of Social Protection for the treatment of *P. vivax*, which consist of Chloroquine (a total of 1,500 mg of oral chloroquine in divided doses: 600 mg initially, followed by 450 mg at 24 and 48 hours after the first dose) and Primaquine for fourteen days (30 mg/day) administered with food. All antimalarial drugs will be given with food, as they can cause stomach pain (gastritis), nausea, and vomiting if taken on an empty stomach. From that day on, volunteers without parasitemia at day 60 will be treated with the same antimalarial regimen. Primaquine will be administered directly and under daily medical supervision for fourteen days in the CIV.

Follow-up after initiation of antimalarial treatment

A TBS will be taken every day after chloroquine treatment until three have yielded negative results are presented consecutively. TBS monitoring will be done on days 7, 14, and 21 after the initiation of treatment to ensure cure. If a volunteer develops a fever or any symptom compatible with malaria, a TBS will be performed again on the day of symptoms and if necessary, an

alternative treatment scheme will be administrated. This therapeutic regimen has been effective in completely controlling the infection in 1-2 days in two recent trials. On day 45th, after starting antimalarial treatment, the volunteers will be evaluated at the CIV by one of the study's physicians; and 10 mL of blood will be taken to measure hematological, renal, and hepatic function and to determine any pregnancy event.

Tracking relapses or recrudescences

There are no documented cases of relapses in Colombia with supervised high doses of Primaquine (30 mg/day/14 days). In our previous studies, no relapses were observed in a 2-year follow-up, although on two occasions, there were reinfections in volunteers who visited the endemic area after the study. All volunteers will be contacted by telephone at 3-month intervals after completing Primaquine treatment and having a final negative TBS.

The resistance of *P. vivax* to Chloroquine has been documented only rarely in Colombia (Comer et al., 1968; Soto et al., 2001) and has not been observed with combined treatment (Chloroquine plus Primaquine) (Soto et al., 2001). However, in the unexpected event that a positive sample is found, at any time, during TBS post-treatment follow-up (days 7, 14, 28 after initiation of treatment), the volunteer (s) will receive alternative treatment with Fansidar® (SP); three tablets in a single dose (25 mg of Pyrimethamine plus 500 mg of Sulfadoxine per pill). If the patient has a contraindication to Fansidar ® (e.g., sulfa allergy), he or she will receive Amodiaquine as described above and will be followed-up with additional TBSs to confirm cure.

Any relapse of *P. vivax* will be repeatedly treated with Chloroquine and Primaquine (in doses identical to the first treatment scheme). The follow-up will be carried out on the same days as the first cycle, as explained. Long-term follow-up will be performed to detect possible relapses due to *P. vivax* hypnozoites. Once the supervised treatment with Primaquine has been completed (2 weeks) and negative TBSs are obtained in the post-treatment controls, all volunteers will be contacted by telephone in the periods defined in Table 2.

Additionally, the study's physician will be the clinical consultant for the rest of the team in all the aforementioned phases and the event of a relapse.

Table 2: Post-treatment telephone follow-up

		**Accepted range**
During the 1st month	Weeks 1, 2, 3, and 4	+/- 3 days
During the 2nd month	Weeks 6 and 8	+/- 5 days

During the 3rd month	Weeks 10 and 12	+/- 7 days
During 6th month	Weeks 16, 20, and 24	+/- 10 days
During the 1st year	Weeks 30, 38, 46, and52	+/- 10 days
During 1.5 year	Weeks 52, 60, 68, and 76	+/- 14 days

All volunteers exposed to the infectious challenge will be encouraged to contact the study physician or their medical health provider to advise them in cases related to malaria diagnosis and treatment and in case of fever. If fever, chills, seizures, or other malaria-related symptoms occur at any time within one and a half year (18 months) after the challenge, the physician in charge of the volunteer should be informed that he/she has been exposed to a CHMI, therefore he must have a TBS test, peripheral blood smear, and RT-PCR. A list of the study procedures, including the follow-up of the volunteers, is shown below:

Figure 2: Summary. Schedule of study procedures

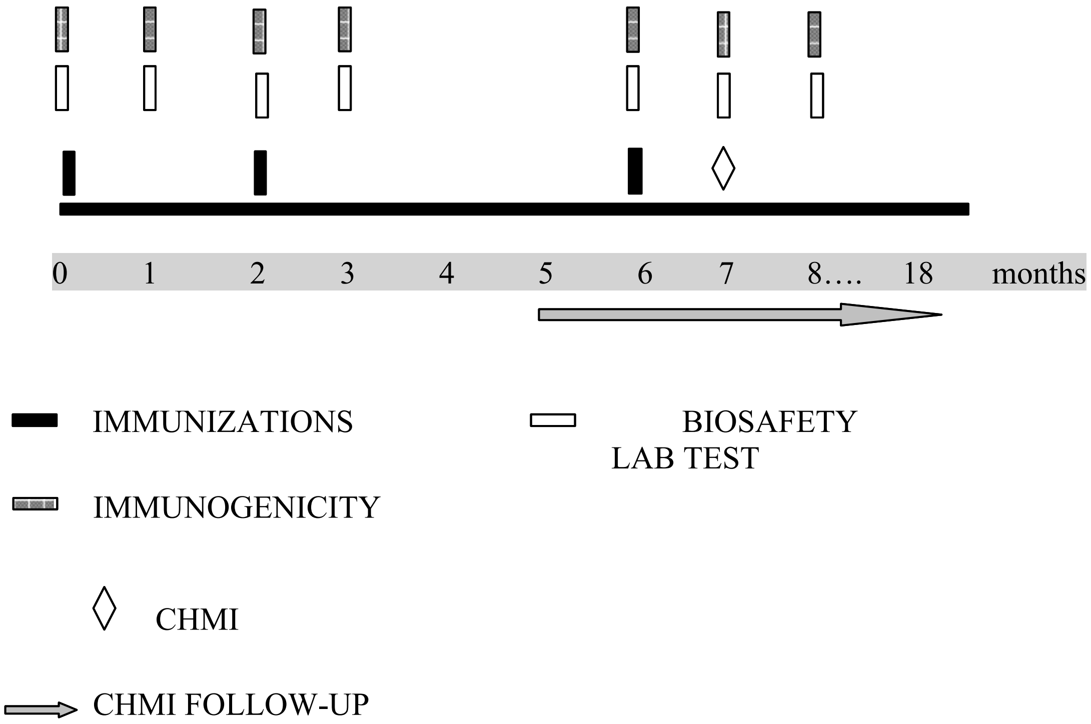

8. LABORATORY TEST

8.1 Malaria diagnosis:
The CIV and ASOCLINIC have been designated by the Valle Departmental Health Secretary as the national diagnostic center to identify malaria infection. Two independent microscopists will read each sample. For thick blood smear examinations, a total of 200 microscopic fields will be examined with oil immersion (x 1000) before reporting that no parasites have been found.

Thick blood smears: will be done in step 2 to select the donor group volunteers and step 3 in the post-challenge and post-treatment follow-up. For the tests, ~2 drops of blood will be collected by fingerpicks. The TBS and peripheral smears will be colored by Field's staining method using the POE CD-POE-001-03 (ASOCLINIC) recommended by the MSP of Colombia.

Malaria RT-PCR (steps 2 and 3): A DNA extraction will be performed from whole blood to perform real-time PCR (RT-PCR) diagnosis for *Plasmodium* (*P. vivax, P. malariae,* and *P. falciparum*) (Rougemont et al., 2004), with a 500 µL sample of donor's blood. This technique is characterized by having an analytical level of sensitivity that detects up to 1 parasite/µL. For this analysis, the primers Plasmo 1 and Plasmo 2 and the species-specific TaqMan probes for *P. vivax* and *P. falciparum* will be used. For each species and assay, positive and negative controls will be used, and the standard curve will be constructed from plasmids for both species to establish and quantify the number of copies per sample.

8.2 Infectious diseases screening test:

Immunogenicity tests will include:

- Determination of native protein recognition by IFA
- Determination of specific antibodies against CS by ELISA
- Determination of T cell response by specific induction of cytokine production IL-2, IL-4, IL-6, IL-10, TNF-α, IFN-γ by ELISPOT and flow cytometry.

All serum samples will be stored at -40°C and cells will be stored in liquid nitrogen until use. The results will be archived in the laboratory for later analysis.

Methodology for objective # 1. *To confirm the safety of the vaccine in naïve volunteers and previously exposed to malaria immunized with PvCS.*

2. **Laboratory tests to establish vaccine safety.**

Complete blood count, PT, PTT, ALT, AST, bilirubin, alkaline phosphatase, BUN, creatinine, partial urine, and pregnancy test (for women) will be performed monthly to determine changes in any of the parameters of these tests. Any alteration will be considered to have a relationship

with the vaccine. If there is any alteration that requires additional clinical or paraclinical studies, these will be carried out along with those already established. Monitoring will continue until these parameters are normalized.

Methodology for objective # 2. *To determine the immunogenicity of PvCS in individuals previously exposed to malaria.*

- **Evaluation of B cell response.** Specific antibody titers will be measured using IFAT and ELISA techniques on day 0 (zero), and subsequently, at months 1,2, 3, 6, 7, and 8, using antigens, sporozoites and synthetic peptides derived from the PvCS. Additionally, the functionality of the antibodies will be evaluated *in vitro* by sporozoite invasion inhibition assays to hepatocytes.
- **Indirect immunofluorescence / IFAT.** For this test, the antigen will be prepared using *P. vivax* sporozoites produced in *An. Albimanus* mosquitoes (Hurtado et al., 1997). IFAT tests will be performed according to the previously described protocol (Herrera et al., 2005). Briefly, the slides will be incubated in a humid chamber in the dark for 1 hour at 37°C, with 25 µl of serum diluted in PBS-BSA 2%, starting with a 1:20 dilution. After three washes in PBS, a FITC-labeled anti-human IgG (1: 100 in PBS-Evans Blue 0.05%) will be added. It will be re-washed and mounted for reading. The antibody titers will be expressed as the last dilution that showed fluorescence.
- **ELISA/enzyme-linked immunosorbent assay.** Antibodies against peptides will be measured by ELISA (Herrera et al., 2005). Briefly, 96-well plates (Nunc-Immuno Plate, Maxisorp, Roskilde-Denmark) will be sensitized with 100 µl of the synthetic peptides N, R, and C at 1 µg / mL overnight at 4 ° C. Subsequently, they will be blocked with 200 µl of PBS1X / 0.05% Tween, 5% milk for 2 hours at room temperature. Then 100 µl of each of the serum samples diluted in PBS 1 X / 0.05% Tween, 2.5% milk, will be added and incubated for 1 hour at room temperature. Subsequently, they will be washed 5 times with 1X PBS / 0.05% Tween. 100 µl of the goat anti-human IgG alkaline phosphatase conjugate diluted 1: 1000 in PBS1x / Tween 0.05%, milk 2.5%, will be incubated for 1 hour at room temperature and four washes will be carried out with PBS 1X / Tween 0.05%. The reaction will develop after 30 min incubation with 100 µL per well of para-nitro-phenol phosphate substrate. The optical density will be determined at 405 nm in an ELISA reader (Dynex Technologies, INC MRX Chantilly VA). A sample is defined as positive when the optical density (OD) of the sample is three times greater than the OD of the negative control

- **Antibody functionality**. It will be evaluated by inhibition of *P. vivax* sporozoites invasion assay using Hep-G2 cells in *in-vitro* cultures. Briefly, the HepG2-A16 cell linage will be maintained in monolayer culture, washed, and suspended in RPMI 1640 medium (Gibco, Grand Island, NY, USA) then they will be placed on Labtek plates at a concentration of 8 x 10 4 cells / 0.3 mL of Williams medium supplemented with L-glutamine, Penicillin / Streptomycin (Gibco BRL, France), Fetal Bovine serum and Dexamethasone. The cultures will be incubated overnight at 37°C in 5% CO2. Before infection, the cells will be irradiated, and 5 x 10^4 *P. vivax* sporozoites diluted in 0.1 mL of medium will be added. Then the serum of the volunteers will be added at a dilution of 1/100 in each well in duplicate; the sporozoites will invade the liver cells for five days. Subsequently, an IFAT will be performed; they will be washed with PBS and fixed with methanol. A specific HSP70 antibody will be added, and the Second fluorescent antibody Alexa 488-labeled with anti-mouse (Molecular Probes®) diluted in 1/200. The degree of maturation of the hepatic stage parasites (schizonts) will be evaluated by fluorescence. The percentage of invasion inhibition will be calculated by the formula [(Average of schizonts of the negative control - an average of schizonts of the sample / Average of schizonts of the negative control) x 100] (Druihle et al., 1998).

2. Evaluation of T cell response.

Cell response will be evaluated by the *in vitro* production of cytokines (cytokines IL-2, IL-4, IL-6, IL-10, TNF-α, IFN-γ in peripheral mononuclear cells (PBMC). Cells will be separated by Ficoll-Hypaque gradients from whole blood (Herrera et al., 2005) and cytokines will be measured using ELIspot and flow cytometry techniques. ELIspot. The production of IL-4 IFN-γ by total PBMC stimulated with the different antigens and/or synthetic peptides will be determined by ELIspot using commercial kits (MABTECH, Stockholm, Sweden). For IFN-γ and/or IL-4, the microplates are sensitized with 5 µg / mL of monoclonal anti-human IFN-γ antibody (1-D1K MABTECH AB, Sweden) overnight at 4°C. Subsequently, 2x105 PBMC / well is added to 1 µg / mL of the peptide (PvCS = N, R, C), using PHA as positive control and RPMI medium alone as a negative control. The plates will be incubated at 37 ° C for 40 hours in a 5% CO2 atmosphere, and washes will be carried out with PBS / 0.05% Tween-20. The anti-IFN-γ monoclonal antibody will be added and/or biotinylated IL-4 (7-B6-1, MABTECH AB, Sweden) and incubated at room temperature for 2 hours. The alkaline phosphatase with streptavidin will be added, and the reaction will be revealed by adding BCIP/NBT (5-bromo-2-chloro-3-indolyl Phosphatase/Nitroblue Tetrazolium) (Sigma, St Louis, MO). Dot-forming cells (SFCs) will be numbered using a counting system (Scanalytics, Fairfax, VA).

- **Determination of cytokines by flow cytometry.** A bead kit with anti-human cytokine antibodies will be used for the simultaneous evaluation of cytokines in the cell culture supernatant (Cytometry Bead Array - CBA) Th1/Th2 Kitt II (Becton Dickinson, Inc. - BD), which allow the determination of IL- 2, IL-4, IL-6, IL-10, TNF-α, and INF-γ. PBMC will be stimulated with each of the PvCS-derived antigens at a concentration of 1 µg / mL, and cell

cultures without the peptide will be used as a negative control. In contrast, cells stimulated with phytohemagglutinin (PHA) will be included as a positive control. The supernatants will be collected on day 4 of culture and used for the determination of cytokine production. Briefly, a standard curve will be prepared according to the instructions of the commercial house. Serum or culture supernatant is added to the tubes with capture beads, and they are incubated for 30 minutes. Beads mixture, the detection reagent-PE, and the wash buffer are added for reading and analysis in the Canto II Facs cytometer.

- **Monocyte phenotyping.** PBMCs, fresh or previously cryopreserved, will be labeled with monoclonal antibodies (mAbs) specific for markers of phenotype, activation, and chemokine receptors following the manufacturer's protocols. The markers that will be used: CD14 PerCP, CD16 APC-Cy7, CD33 PE, CD56 PE Cy7, CD83 FITC, HLA-DR FITC, and CCR2 APC (BD Biosciences, San José, USA). Control isotypes will be used in all experiments. The acquisition of the cells will be made on the BD Biosciences FACS-Canto II flow cytometer (Becton Dickinson, San José, USA), and the data will be analyzed using the FlowJo program (TreeStar). MO will be initially identified and selected for size and complexity. Subsequently, regions will be defined excluding NK cells (CD56hi) and dendritic cells (CD83+), but including CD14+ and CD33+ cells. OM profiles will be classified into: i) "classic OM" (CD14hi/CD16-), ii) "non-classical, pro-inflammatory OM" (CD14dim/CD16+) secreting TNFα, and iii) "anti-inflammatory OM or intermediates "(CD14hi/CD16+) with strong HLA-DR expression.

- **Phenotyping of B lymphocytes**. PBMCs, fresh or previously cryopreserved, will be labeled with specific mAbs for phenotype and activation markers following the manufacturer's protocols. The markers that will be used are CD19 PerCP, CD20 APC-Cy7, CD21 PE-Cy7, CD27 APC, CD10 PE, CD95 FITC, anti-human IgG FITC (BD Biosciences, San José, USA). Control isotypes will be used in all experiments. The cells will be acquired on the BD Biosciences FACS-Canto II flow cytometer (Becton Dickinson, San José, USA) and the data will be analyzed using the FlowJo program (Tree-Star). LB profiles will be classified as: i) "classical" (CD19+, CD27+, CD21+, CD10-), ii) "atypical" (CD19+, CD27-, CD21-, CD20 +, CD10-), and iii) "activated" (CD CD19+, CD27+, CD21-, CD20+, CD10-) Evaluation of the innate immune response with specific markers by flow cytometry or image cytometry

Methodology for objective # 3: *To determine the protective efficacy of the vaccine against control human malaria infection with viable P. vivax sporozoites.*

The infection clinical manifestations will be monitored by a physician, and TBS and PCR diagnosis will be made from the 7th day post-infection; once the volunteer is diagnosed positive for malaria, he/she will be treated following the MSP. The incidence of infection, the pre-patent period, the severity and frequency of symptoms will be compared between the study groups.

- **Thick blood smear** Slides will be stained with Giemsa and read independently by two experienced microscopists. Parasitemia will be quantified by observing the microscopic fields corresponding to 300 leukocytes and the estimated count of leukocytes per µL of blood.
- **Diagnostic RT-PCR.** It will be made with genomic DNA obtained from the parasite from 500 µL blood samples from the study volunteers. Species-specific detection of *Plasmodium* will be carried out using the previously described and validated RT-PCR technique (Rougemont et al., 2004).

Methodology for objective # 4: *Evaluate the infective capacity of early P. vivax gametocyte stages in Anopheles albimanus mosquitoes.*

To evaluate the infective capacity of *P. vivax* gametocytes in Anopheles albimanus, during the infectious challenge phase (Step 3) all volunteers will be directly exposed to the bite of 20 uninfected mosquitoes from the fifth-day post-infective bite and every two days until the microscopic diagnosis is confirmed or until the 15th day as follows: on days 5, 7, 9, 11, 13 and 15) according to POE EN-02-POE-003 for a direct mosquito bite.

Mosquitoes will be placed in 7 x 7 x 15 cm "feeding cages" to be placed on the forearm or in the place of preference of each individual for 10 minutes. Mosquitoes will be evaluated for the presence of oocysts on day 7 according to POE EN-02-POE-002 and the presence of sporozoites on day 14 according to POE EN-03-POE-001.

8.2.1. Results interpretation

B cell response: Due to the low antibody titers induced by *P. vivax* under natural conditions (compared to *P. falciparum*), the responses in this study will be quantified as low, medium, and high, taking into account that any positive reaction will indicate a previous contact status with the parasite and anti-malaria immune response in the volunteers (Table 3).

Table 3. Antibody titers against *P. vivax*

Technique	Low	Medium	Hight
**IFAT**	<1:40	>1:40- 1:320	> 1:320
**ELISA**	1:100 -1:500	>1:1500 -1:5000	>1:5000

T-cell response. The ELIspot technique will determine cytokine production. In these techniques, the colony-forming cells (sport forming cells SFC) will be quantified, which will be

expressed as i) the average number of SFCs per 106 PMBC and will be considered significant if the average number of cells in each well with the experimental antigen is greater than the well with the control antigen (P <0.05, student t-test), ii) the net number of SFCs per well (average of SFCs in the well with the experimental antigen minus the number of SFCs in the well with the antigen control) is greater than 5 SFCs per well and, iii) The average rate between the SFCs in the well with the experimental antigen and the average of SFCs in the well with the control antigen is more significant than 2.0. As for flow cytometry, the number of events to be counted in the cytometer (FACS Canto II) will be 1,800; it will run the samples from the tubes and automatically acquire the data through the software that will evaluate the data in FSC, SSC, FL1, FL2, and FL3.

MO and LB subpopulations: the percentage of specific MO and LB surface markers will be obtained and the mean fluorescence intensity of each group for classification of the populations. Average values of each of the groups will be compared with each other and during the follow-up.

9. Data handling and record-keeping

The data obtained from the study will be entered into a database designed with the RED-cap program (Vanderbilt University, 2012) and stored on a server with access restricted by password. The data will be entered in the Electronic Case Report Form by the study personnel, verified by the clinical monitor according to standard operating procedures, and corrected if necessary by the authorized investigator. The verification of the data consigned in the FRC will be done, taking as reference the source documents and comparing them with the database's printed data. The electronic FRC will not be considered as a source document in any case. The Clinical Monitor will report inconsistencies to be reviewed and corrected by authorized personnel. After quality control, the information will be analyzed with Stata analysis and will be carried out using the statistical tests indicated according to the type and distribution of variables. The level of significance for the statistical tests will be $\alpha = 0.05$.
The differences between the groups when variables studied are dichotomous will be analyzed using the Chi2 test (Fisher's exact test will be used when the data number is less than 5 (comparison of continuous variables between the groups will be done using T-student test. The comparison between several groups will be done through one-way ANOVA analysis (Scheffe evaluation for post-hoc analysis).

9.1 1 Record-keeping

During the study's development, the CRFs, the participants' source documents, the informed consents, the inclusion questionnaires, and all the information pertinent to the volunteers will be kept in a safe place at the CIV. The electronic databases will be stored in non-rewritable

optical media. Participant records will be transported by authorized research personnel in a portable, safe and waterproof box (CIV Carrera 37 2Bis No.5E-08, Cali, Colombia). Once these documents have been used, they will be archived again at the CIV. At the end of the study, all reports, consent forms, questionnaires, and other pertinent records of the protocol will be archived in the VIC for 10 years, after which they will be identified as dead archives.

10. RISKS FOR THE VOLUNTEERS, THE RESEARCH GROUP AND THE ENVIRONMENT; PRECAUTIONS TO MINIMIZE THE RISK.

Using the control human malaria infection described with *P. vivax* sporozoites, our group exposed 35 healthy naïve volunteers to infection in two consecutive trials that proved to be safe with infective doses between 2-10 bites. The infections showed prepatent periods that ranged from 9-18 days with an approximate average of 11 days. The duration of symptoms was similar in all volunteers (1.5-4.5 days), and their responses to treatment were rapid and similar in all volunteers. All volunteers cleared parasitemia within the first 48 hours after treatment (Herrera, et al., 2009; Herrera, et al, 2010). In our previous studies, the prepatent period was evaluated by TBS and PCR from day 7. In some cases, the PCR detected parasitemia before the TBS but in none of the cases, the PCR detected it before 9 days. The TBS was sensitive, detecting levels of parasitemia as low as those previously described (geometric mean of 46 parasites/µL).

Likewise, with *P. falciparum,* hundreds of volunteers have been safely and reproducibly infected in the United States, 97% of these volunteers developed moderate symptoms and short duration (average duration, 3 days) (Hoffman, 1997). These volunteers could be treated without complications due to the early initiation of treatment when the parasitemia was still very low (geometric mean of 46 parasites/µL) and also because the sensitivity of the parasite to the antimalarials used was known.

A splenic rupture is an infrequent event, which has only been observed in patients with chronically established infection (Yagmur, et al., 2000). In the previous challenge study, only one of the 17 volunteers had splenomegaly as an adverse event related to the infection. (Herrera, et al., 2010) In the proposed study, the volunteers will be closely followed and treated immediately if the parasite's presence is detected.

<u>10.1 Risks for blood donor volunteers.</u>

Potential risks associated with donating blood may include redness, itching, infection at the puncture site, or vasovagal symptoms such as dizziness and fainting. The sample collection will be done by venipuncture under aseptic and antiseptic conditions; new and disposable sterile material will be used. A study physician will provide primary medical care to treat vasovagal episodes (lipothymia).

A short delay (10-15 minutes) in receiving the first dose of antimalarials may be associated

with donating blood; however, this risk will not significantly affect the volunteer's recovery. Every effort will be made to expedite the procedures so that antimalarial treatment can start as quickly as possible. A complete blood count will be performed to detect hematological alterations related to malaria.

There is a potential risk that an HIV-positive result in a volunteer is not appropriately handled and creates adverse effects on their personal and/or work environment. This risk will be reduced by strictly complying with the confidentiality rules. Volunteers will personally receive a copy of the results one week after. In case of presenting a positive result for any of the infectious diseases, they will be referred to their health provider following Law 100, Article 179 of 1993 or, failing that, to the Departmental Health Secretary following Law 1543 of 1997, Chapter II of the Ministry of Health and Social Protection, to provide advice and medical care. If a volunteer already has health insurance, he or she will be referred to their private physician with the test results. These results will only be given to the volunteer.

10.2 Risks to volunteers associated with CHMI

Risks associated with CHMI include a very low risk of anaphylaxis, possible transmission of other infectious agents through mosquito bites, and risk associated with the use of antimalarial drugs.

Precautions to minimize the risk related to the malaria challenge:

- **Anaphylaxis management:** In the place where the challenge is to be carried out, medications for anaphylaxis management such as Epinephrine 1:1000, Diphenhydramine, Cimetidine, and Methyl-prednisolone will be available, which will be used by the research physician who will remain in the area of infection. An ambulance will be available and used to transport the volunteer who needs it from the Entomology Unit to IMC, a transfer that takes approximately 30 min.
- **Blood screening:** Blood from donors with *P. vivax* infection will be screened for infectious diseases as described above.

Selection of volunteers and follow-up: Volunteers will be selected if they meet each of the inclusion criteria. They will be monitored, and once the infection is documented, the volunteer will begin treatment according to the protocol. Early treatment will minimize the risk of developing severe complications, usually unusual in *P. vivax* infections. To ensure adequate follow-up of the volunteers, each of them will have all the research group data. Under the carefully controlled conditions implemented for this study, the possibility of making a late diagnosis is remote. Some transient abnormalities such as fever, headache, myalgia, nausea, vomiting, mild anemia, leukopenia, thrombocytopenia, and asthenia may occur during the infection, which is very unlikely when an early diagnosis is made. The only severe complication and direct infection of *P. vivax* in healthy adults is splenic rupture (Yagmur, et al., 2000), which is highly unlikely if the diagnosis is made as soon as parasitemia occurs, and

treatment is administered without delay. However, as a precaution, volunteers will be informed of this risk. They will be advised to avoid doing contact sports or any strenuous activity that may result in abdominal trauma, especially during the two weeks following the start of treatment.

- **Pregnancy and *Plasmodium vivax* infection:** Although the effects of *P. vivax* malaria during pregnancy are less severe than those of *P. falciparum* (Nosten, et al., 1999), *P. vivax* infection during pregnancy has been associated with high maternal parasitemia (compared to parasitemia in non-pregnant women), maternal anemia, and low birth weight (Nosten, et al., 1999; Singh, et al., 1999). Women will be guided to use contraception for at least six months after the challenge. Women participating in the study will be advised to inform their physician on time of their participation in the clinical trial in the event of pregnancy. If any of the women relapse with *P. vivax* while pregnant, they will be treated immediately, significantly reducing the risk to the mother and the fetus of having an adverse pregnancy outcome. Chloroquine is safe to be used during pregnancy (McGready, et al., 2002), as is amodiaquine to be administered as an alternative therapy. Primaquine treatment will be administered after pregnancy.
- **Relapses:** Supervised therapy with chloroquine and high doses of primaquine will be administered. No cases of relapses of *P. vivax* have been documented with the administration of a supervised combined regimen of primaquine and chloroquine (Baird, et al., 2002; Hoffman, et al., 2002). Volunteers will be followed closely after treatment.

10.3 Risks to volunteers associated with malaria treatment.

Potential side effects of antimalarial medications include nausea, vomiting, diarrhea, abdominal pain, vertigo, headache, sleep disturbances, blurred vision, itching, tinnitus, and photosensitivity. The FDA has reported the following adverse reactions in connection with the use of these medications:

- **Chloroquine phosphate**: Gastrointestinal reactions (vomiting, nausea, diarrhea, cramps), mild transient headache, hearing effects such as nerve deafness, tinnitus, and decreased hearing acuity in those with a history of it. Visual effects, dermatological reactions (pruritus and alopecia), and cardiovascular reactions (hypotension or changes in the EKG) may also occur. The use of chloroquine is contraindicated in people with psoriasis or another type of dermatological pathology.
- **Primaquine**: The most frequently encountered side effects are gastrointestinal disorders such as nausea and abdominal discomfort, especially if the drug is administered on an empty stomach. Primaquine will be administered with food intake in this study. The Primaquine has been reported as the cause of leukopenia and mild methemoglobinemia is present in most individuals. The concomitant use of substances that predispose to this side effect, such as sulfonamides, should be avoided. Primaquine is not recommended in pregnant

women. The administration of a dose of 30mg per day of Primaquine for more than a year in healthy adults has proven to be well tolerated as long as it is accompanied by food intake. There are no significant effects related to kidney or liver damage, evidenced by serum creatinine BUN, AST, ALT, LDH, alkaline phosphatase, and the methemoglobinemia that occurs is reversible and asymptomatic (Fryauff, et al., 1995).

- **Falcidar® (Sulfadoxine - Pyrimethamine)**: Toxic manifestations are rare and usually attributable to the sulfadoxine component. Severe skin reactions (such as erythema multiform, Steven-Johnson syndrome, and toxic epidermal necrosis) have been reported in individuals using a weekly schedule as prophylaxis. The safety of the combination during pregnancy has not been established, but the drug has been used to treat a large number of pregnant women.

- **Amodiaquine:** The adverse reactions of amodiaquine are generally similar to those of chloroquine, the most common being nausea, vomiting, abdominal pain, diarrhea, and itching; a less common effect is bradycardia. There is evidence that itching is less common with amodiaquine than with chloroquine.

Treatment Precautions: Volunteers will receive supervised treatment, allowing close monitoring to observe side effects. The adverse events that appear will be documented as well as the potential associations with the treatment, which will receive a score of causality.
In the previous trial, the adverse events most frequently associated with treatment were gastrointestinal origin (nausea, dizziness, and epigastric pain). The symptoms reported by the volunteers did not significantly affect their daily activities.

10.4 Risk for those conducting the study

There is a low risk for the workers who are in charge of the collection and the processing of the sample of presenting accidents with the needles of the volunteers.

Personnel Precautions: To reduce risk, all workers in contact with blood or blood products should strictly follow standard precautions. Also, the blood of volunteers with HIV, hepatitis B, or Hepatitis C infection will be excluded from the study.

10.5 Risk and precautions associated with the environment.

The risk of accidental malaria transmission to someone in the community is negligible; infected mosquitoes will only be found in a restricted area of the insectary and will not be removed outside of this location at any time. Infections in volunteers will be treated quickly before

gametocytes can develop (this time is generally 10 days after the first appearance of parasites in the blood). Volunteers can only be in the area of Cali, which is not an endemic area so the natural transmission of malaria does not occur. If any of the group members are accidentally bitten by an infected mosquito or develop symptoms of malaria, they will be immediately evaluated with TBS to confirm the presence of an infection. If the result is positive, treatment with standard doses of chloroquine and Primaquine will be given under supervised therapy.

11. BENEFITS

11. 1 Benefits for blood donor volunteers.

There will be no direct benefits from participating in this study. However, volunteers will receive indirect benefits such as blood tests screening for infectious diseases. In the event of a positive result for an infectious disease other than malaria, including HIV, the volunteer will be referred to the corresponding health provider according to the social health security scheme to which he/she is affiliated, with a copy of his or her results, for advice and medical assistance

11.2 Benefits for volunteers enrolled in the malaria challenge.

There are no direct benefits for volunteers participating in this study. However, volunteers may receive some indirect benefits, such as a complete blood screening that includes the screening of infectious diseases. If during the selection phase any infectious disease screening test turns out positive, including HIV, the volunteer, with a copy of the results, will be directed to the corresponding health provider according to the social health security scheme to which he/she is affiliated for obtaining counseling and medical assistance following Law 100, Article 179 of 1993 or to the Valle Health Department following Law 1543 of 1997, Chapter II of the Ministry of Health.

12. COMPENSATION

12.1 Compensation for blood donors.

Volunteers will not incur financial expenses derived from the study participation; however, as required by the Ministry of Social Protection, no monetary compensation should be made. The medical doctors evaluating volunteers will provide counseling if a pathology other than malaria is diagnosed. Transportation costs will be recognized, a complete medical evaluation will be made, and they will receive advice to find any associated pathological condition. Volunteers will be provided a snack after the blood donation.

12.2 Compensation for infectious challenge volunteers.

There will be no direct compensation derived from the participation of volunteers in this study. However, the volunteers will receive indirect benefits such as infectious disease screening and

other laboratory tests. Volunteers will not incur financial expenses derived from the study participation; therefore the costs of transportation and snacks on the day of the challenge and the days of follow-up will be covered by the study. Additionally, each time a volunteer is summoned for a procedure related to the study, the sum corresponding to one day of the current legal minimum wage will be delivered as a symbolic way of compensating the dedication of time to the development of the study.

13. CRITERIA FOR DROPPING/WITHDRAWAL OF VOLUNTEERS.

Volunteers may freely withdraw at any time during the study. If the volunteer leaves the study, he will be treated when he/she leaves or withdraws from the study using the protocols described. If a volunteer is excluded from the study for any reason, a final evaluation (physical and laboratory exams) will be performed. The reason for the withdrawal of any of the volunteers will be reported in a CRF and accompanied by supporting information.

On the other hand, regardless of the volunteer withdrawal, the CIV will provide him with timely treatment and medical care in the event of malaria and/or one of its related complications.

14. ADVERSE EVENTS.

An adverse event (AE) is considered as any sign, inconvenience, damage, dysfunction, adverse reaction to a drug, or any other undesirable result that occurs in any of the volunteers participating in the study, even those that have already been defined as expected risks. Each of these events will be reported in a CRF and given a degree of severity and causality related to the study activities (example: blood donation or malaria challenge).

The intensity of the adverse event recorded in the CRF corresponds to the highest grading during an episode. For example, if a person has a fever, the intensity is graduated according to the highest temperature recorded.

Adverse events will be divided into two groups: requested and unrequested. Requested adverse events: will be asked at every contact with the volunteers by the clinical trial staff and recorded in a CRF in the periods determined as described below.

- Local adverse events, occurring in the region of the body where the volunteers were exposed to mosquito bites. They will be verified from the moment of the challenge and up to 7 days after it.
- All local adverse events will be recorded in CRFs. Their intensity will be classified according to the following table, adapted from the document "Guidance for Industry - Toxicity Grading Scale for Healthy Adult and Adolescent Volunteers Enrolled in Preventive Vaccine Clinical Trials

(FDA, 2007) ”.

Requested local adverse events

Local Reaction	Grade 1	Grade 2	Grade 3	Grade 4
Pain	Does not interferes with the activity	Repeated use of NSAIDs > 24 hours or that interferes with the activity	Any use of opioid analgesics or that interferes with daily activity	Emergency care for > 12 h or hospitalization requirement
Sensibility	Mild discomfort to the touch	Discomfort with the movement	Significant discomfort at rest	Emergency care for > 12 h or hospitalization requirement
Erythema	2.5-5 cm	5.1-10 cm	> 10 cm	Necrosis or exfoliative dermatitis
Induration	2.5-5 cm and does not interfere with the activity	5.1-10 cm or that interferes with the activity	> 10 cm or that interferes with daily activity	Necrosis

- Systemic adverse effects will be verified from the moment of the challenge and up to 7 days after the termination of antimalarial treatment. Systemic events may be due to the body's reaction to the challenge or antimalarial drug administration. The events that occurred from the time of the challenge to the diagnosis of malaria will be attributed to *P. vivax* infection. Events occurring from the time of administration of antimalarial treatment up to 7 days after its termination will be attributed to the drug. A margin of 7 days is given after the treatment since there are sub-therapeutic levels of the drug circulating during this period.
- All systemic requested adverse events will be recorded in the corresponding CRF according to the following table, adapted from the document “Guidance for Industry - Toxicity Grading Scale for Healthy Adult and Adolescent Volunteers Enrolled in Preventive Vaccine Clinical Trials (FDA, 2007)”.

Required systemic adverse events

Systemic reaction	Grade 1	Grade 2	Grade 3	Grade 4
Disease or adverse clinical event	Does not interferes with the activity	Interferes with activity but does not require medical intervention	Interferes with activity and require medical intervention	Emergency care for > 12 h or hospitalization requirement
Nausea	Does not interferes with the activity	Interferes with activity	Interferes with daily activity	Emergency care for > 12 h or hospitalization requirement

Emesis	1-2 episodes	> 2 episodes	Require LEV at home	Emergency care for > 12 h or hospitalization requirement
Diarrhea	2-3 loose stools	4-5 loose stools	6 or more loose stools or require LEV at home	Emergency care for > 12 h or hospitalization requirement
Headache	Does not interferes with the activity	Repeated use of NSAIDs > 24 hours or that interferes with the activity	Any use of opioid analgesics or that interferes with daily activity	Emergency care for > 12 h or hospitalization requirement
Fatigue	Does not interferes with the activity	Interferes with activity	Significant, interference with daily activity	Emergency care for > 12 h or hospitalization requirement
Myalgia	Does not interferes with the activity	Interferes with activity	Significant, interference with daily activity	Emergency care for > 12 h or hospitalization requirement

Vital signs

Vital Signs	Grade 1	Grade 2	Grade 3	Grade 4
Fieber °C	38-38.4	38.5-38.9	39-40	> 40
Tachycardia l/m	101-115	116-130	> 130	Emergency care for > 12 h or hospitalization requirement
Bradycardia l/m	50-54	45-49	< 45	Emergency care for > 12 h or hospitalization requirement
Hypertension (sistolic) mmHg	141-150	151-155	> 155	Emergency care for > 12 h or hospitalization requirement
Hypertension (diastolic) mmHg	91-95	96-100	> 100	Emergency care for > 12 h or hospitalization requirement
Hypotension (sistolic) mm Hg	85-89	80-84	< 80	Emergency care for > 12 h or hospitalization requirement
Respiratory frequency r/m	17-20	21-25	> 25	Intubation

Serum

Serum	Grade 1	Grade 2	Grade 3	Grade 4
Glucose - Hypoglycemia	65-69	55-64	45-54	< 45
Random glucose-Hyperglycemia	110-125	126-200	> 200	Requires insulin or hyperosmolar coma
BUN mg/dL	23-26	27-31	> 31	Requires dialysis
Creatinine mg/dL	1.5-1.7	1.8-2.0	2.1-2.5	Requires dialysis

ALT,AST increase in factor	1.1-2.5 x ULN	2.6-5.0 x ULN	5.1-10 x ULN	> 10 x ULN
Bilirubin - accompanied by an alteration in AST/ALT increase in factor	1.1-1.25 x ULN	1.26-1.5 x ULN	1.51-1.75 x ULN	> 1.75 x ULN
Bilirubin - without alteration in AST/ALT increase in factor	1.1-1.5 x ULN	1.6-2.0 x ULN	2.0-3.0 x ULN	> 3.0 x ULN

Hematology

Hematology	Grade 1	Grade 2	Grade 3	Grade 4
Hb women - gr/dL	11-12	9.5-10.9	8.0-9.4	< 8
Hb men - gr/dL	12.5-13.5	10.5-12.4	8.5-10.4	< 8.5
Leukocytosis - cells/mm3	10,800-15,000	15,001-20,000	20,001-25,000	> 25,000
Leukopenia - cells/mm3	2,500-3,500	1,500-2,499	1,000-1,499	< 1,000
Lymphopenia - cells/mm3	750-1000	500-749	250-499	< 205
Neutropenia - cells/mm3	1,000-1,499	500-999	499-300	< 300
Eosinophils - cells/mm3	650-1500	1501-5000	> 5000	Hyper-eosinophilia
Thrombocytopenia - cells/mm3	125,000-140,000	100,000-124,000	25,000-99,000	< 25,000
PT - increase in factor	1.0-1.1 x ULN	1.11-1.20 x ULN	1.21-1.25 x ULN	> 1.25 x ULN
PTT - increase in factor	1.0-1.2 x ULN	1.21-1.4 x ULN	1.41-1.5 x ULN	> 1.5 x ULN

Note: The lower cut-off point for neutrophils is set below the reference range due to the association between benign ethnic neutropenia and Afro-descendant populations.

Urine

Urine	Grade 1	Grade 2	Grade 3	Grade 4
Proteinuria	Traces	1+	2+	Hospitalization or dialysis

Glucosuria	Traces	1+	2+	Hospitalization or hyperglycemia
Hematuria (microscopic) - red blood cells/field	1-10	11-50	> 50 or macroscopic	Hospitalization or red blood cells transfusion

Non-requested adverse events: They correspond to all the adverse events presented by the volunteers that were not considered among the requested adverse events. All these events will be recorded in a CRF from the moment of the challenge until 7 days after antimalarial treatment ending. After this moment, the non-requested AEs will only be registered in the CRF corresponding to events related to treatment or that indicate malaria suspicion. In the case a volunteer visits an endemic malaria area, he/she will be subjected to blood sampling on filter paper, in addition to the routine TBS diagnosis. This sample will define a new malaria infection or a relapse due to the clinical trial.

To grade the severity of unrequested AE, the values assigned for symptoms, signs, and laboratory results in the Common Toxicity Criteria will be applied. The classification of clinical AE will be made according to the clinical judgment of the evaluating physician and the principal investigator and per the categories specified in the document "Guidance for Industry - Toxicity Grading Scale for Healthy Adult and Adolescent Volunteers Enrolled in Preventive Vaccine Clinical Trials (FDA, 2007). If there is any alteration that requires additional clinical or paraclinical studies and other controls, these will be carried out. The monitoring will be carried out until these parameters normalize. The degree of severity of the symptoms will be assigned by the doctor after evaluating the volunteer and following the definitions described below:

- Grade 1= Mild
- Grade 2= Moderate
- Grade 3= Severe
- Grade 4= Potentially life-threatening

Mild: It is a transitory, self-limited event, with the presence of minor symptoms that do not interfere with the development of the individual's everyday activities (for example, the volunteer can work or study) and does not require any medical intervention. Example: Mosquito bite site: pain and erythema; malaria: myalgia

Moderate: Events that require minimal medical intervention to improve the volunteer's condition. In these cases, once the intervention is carried out, it is expected that the individual can perform normal daily routine activities; there may be a degree of functional limitation. Example: Mosquito bite site: Itching and/or enough pain to limit movement; malaria: Fever that improves with non-steroidal anti-inflammatory drugs.

Severe: Symptoms that require treatment and prevent the individual from effectively developing daily activities. Volunteers with a severe adverse event are generally unable to work but can be safely managed as outpatients—example: malaria: flu-like reaction or fever that results in prostration.

Potential life threat: Any event that results in emergency care for a period greater than 12 hours or requires hospitalization. Example: bronchospasm requiring parenteral medication in the emergency room or seizures assessed in the emergency room but not resulting in hospitalization.

14.1 Serious Adverse Events:

The serious adverse events will be reported following the classification of the Document of the Americas as described below:

4. results in death,
5. life-threatening requires patient hospitalization or prolongation of existing hospitalization
6. results in persistent or significant disability, or is a congenital anomaly/congenital disability

14.2 Classification of the AEs- Association to the study activities.

In agreement with the GCP norms, adverse events can occur during any interaction with the volunteer, including at the time of screening and selection and during the study processes or subsequent follow-ups. Each of the events will be classified as definitely related, probably not related, possibly related, or not related to the study activities (e.g., blood sampling, challenge, or antimalarial treatment). This classification will be made according to the medical judgment of the principal investigator and the other evaluating physicians who assess adverse events.

Degrees of Causality:

6. *Unrelated*: The event has no temporal relationship with participation in the research and is definitely related to another etiology.

7. *Probably not related:* The time of onset and the nature of the event are temporarily not related to the intervention carried out in the investigation.

8. *Possibly related:* The timing and nature of the adverse event may be a result of participation in the research, but another explanation may be more likely.

9. *Probably related:* The timing and nature of the adverse event suggest that it is related to study participation (e.g., arm erythema followed by mosquito bite). A different potential etiology is apparent but less likely.

10. *Definitely related:* Those adverse events that have a temporal relationship with the intervention under study cannot be attributed to another etiology.

The appearance of the AEs will be classified as expected or unexpected. In this study, severe or serious AEs are not expected to occur.

14.3 AEs REPORT.

Each one of the AEs presented by the volunteers related or not to the study procedures will be consigned in a CRF according to the GCP standards.

Serious AEs or those that are life-threatening for the volunteers and that are classified as possibly or probably related to participation in the study will be detailed reported electronically, by telephone, or by any other appropriate means to the ethics committee and to the clinical monitor Ricardo Palacios (Telephone: 55-11-939-40670), within the first 24 hours after its appearance, including its severity and potential impact on the other participants.

A written report will also be made, which will be sent to the entities already mentioned. This report should include the following points:

- AE report date.
- Volunteer’s code.
- Date of birth, gender, and ethnicity of the volunteer.
- Name of the principal investigator.
- Study step in which the severe adverse event appeared.
- Procedures performed on the volunteer during the study and their corresponding dates.
- Date of the appearance of the serious AE.
- Full description of the serious AE.
- Signs or symptoms of the serious AE and its causality.
- Interventions carried out on the volunteer after the serious AE, including drugs administered with their doses, route of administration, and the date of the first and last dose.
- Date of the resolution of the serious AE or death.
- Consequences for the volunteer’s health and on his permanence in the trial.
- Assessment and categorization of serious AE in relation to the study activities.
- Specific recommendations to guarantee the safety of volunteers, which can be translated into changes to the protocol.

The written report will be reviewed by the local clinical safety monitor and will subsequently be sent to the ethics committee's presidents; this will be done within the first three business days after the AE is presented. All the AEs and the interventions will be recorded in the file of each of the volunteers and will be included in the reports made to the ethics committees.

14.4 AEs follow-up period.

All adverse events will be followed until the outcome is classified into one of the following options:

7. Fatal
8. Unsolved
9. Resolved
10. Resolved with sequels
11. In resolution
12. Unknown

Pregnancies that have occurred in the period between the infectious challenge and 7 days after completing the antimalarial treatment will be followed up by the clinical trial group until delivery.

15. ETHIC CONSIDERATIONS

15.1 Approval of the ethics committees and organization plan.

The protocol will be submitted for review in Colombia to the Human Ethics Committees. This protocol contains the IC forms that must be signed by the participants (Annex A, B, and C) which includes the conditions on the nature and scientific integrity of the research and the information about the guarantees provided to the study volunteers. During the study, the principal investigator will be responsible for reporting on all events that may affect the safety of individuals and the continuation of the clinical trial. Recruitment activities cannot begin until the local Ethics Committees issue their approval.

15.2 Ethics committees affiliation to the United States FWA.

The CECIV is registered with the United States FWA (Federal Wide Assurance) for the protection of human subjects of the United States Department of Health and Human Services (DHHS), Office of Human Research Protection (OHRP), under the guidelines of regulation 45CFR46.103. (CECIV: FWA: FWA00016072,). The activities of these institutions with human subjects and all the activities of the Ethics Committee (IRBs) will be conducted following the dispositions of the Declaration of Helsinki. (As they have been adopted in 1996

or 2000).

15.3 Research-related injuries.

Once the volunteers are included in steps 1 and 3 of the study, they will be affiliated to the contributory regime of the General System of Social Security in Health (Health Provider Company -EPS), to a prepaid medicine service, and a life insurance policy, these services will be provided at no cost to them and for the entire time of the study.

If the volunteer belongs to the subsidized regime, they will be transferred to the contributory regime and affiliated to the prepaid medicine service. If he/she already belongs to the contributory regime, the center will assume the payments of the volunteer and will affiliate him in the same way to the prepaid Medicine service. However, if the volunteer is a beneficiary of the “identification system for beneficiaries of social subsidies” (SISBEN), he/she may decide whether to continue in it so as not to lose the different benefits and subsidies it provides, renouncing the benefit of affiliation to the contributory regime and to prepaid Medicine, which is provided for the participation in the study. This way, the volunteer will only get the benefit of life insurance

Participants that result injured due to their cooperation with the study will get medical care at no cost to them in a health institution of level III complexity; they will not receive any other compensation derived from the injury. This situation does not eliminate the volunteer's right to seek legal assistance to which he is entitled.

16. CIV AND ASOCLINIC GOOD CLINICAL PRACTICES (GCP) AND GOOD LABORATORY PRACTICES (GLP).

The CIV located in Cali will be the place to carry out the post-challenge follow-up visits to the volunteers. The CIV was created in 2000 under the advice of the WHO (World Health Organization) as part of the TDR program (Tropical Diseases Research and Training Program) WHO/TDR and is currently developing a training program in Good Laboratory Practices (BPL) (M. Arévalo- Herrera Ph.D.). Additionally, the WHO special program for Research and Training in Tropical Diseases (TDR) has provided support and guidance for the establishment of GCPs in the CIV. The WHO/TDR chose Dr. Ricardo Palacios as the monitor of the phase I clinical trial to assess the safety and immunogenicity of the PvCS vaccine candidate for *P. vivax* in two clinical trials we developed between 2005 - 2008. Dr. Ricardo Palacios has remained linked to the CIV and later on participated as an external monitor of the two previous challenge trials (Herrera, et al, 2009; Herrera, et al, 2010). The Meridional R&D company, founded and directed by him in São Paulo (Brazil), has adapted these monitoring procedures and is authorized to act as a Contract Research Organization (CRO) by the National Council for Scientific and Technological Development (CNPq) of Brazil. During the last 5 years, most of the group

members in Cali have participated in GCP workshops organized and sponsored by the NIAID in Brazil and the USA and by other agencies in Colombia. The quality controls related to the study materials will be ensured by both the clinical monitors and the CUIC.

The screening will be carried out according to the standard screening parameters implemented at the Blood Bank and to the standards required by the Ministry of Social Protection, in a clinical laboratory duly authorized to perform these activities.

17. CONFIDENTIALITY

All information collected from volunteers will be kept strictly confidential. Each person who participates in the selection process will be assigned a 5-digit identification code. Although the names of the participants will be available in the inclusion form, this information will be kept under lock and key, as noted above. All volunteer data will be entered into the electronic database in the REDcap program. The list with the names and codes of the volunteers will have a username and password that will only be accessed by staff authorized by the Principal Investigator. In case of finding any anomaly in the results of the tests of the volunteers, they will be contacted as soon as possible to personally deliver the laboratory reports and medical recommendations. The records may be examined by monitors, auditors, and/or regulatory authorities. All individual reviews of records are bound by strict confidentiality rules.

18. RULES FOR STUDY INTERRUPTION.

This study will have a total duration of 6 months from the moment of inclusion of the volunteers. The clinical study monitor and the principal investigator will review all serious AEs according to the GCP guidelines. The occurrence of serious AEs possibly related to the study procedures under consideration by the principal investigator and the clinical monitor will lead to the suspension of the study. All review institutions and ethics committees will be informed of the development of these serious EAs by the Principal Investigator and the monitor. The CRFs will be reviewed by the clinical monitor and sent to the presidents of the IRBs within no more than three business days. The ethics committees will review the AEs and decide whether the study can continue or not. The CIV will also review the reports as well as the recommendations of the IRBs, and will make the final decision regarding the continuation of the study. The principal investigator will be informed by the CIV of the final decision

19. USE OF THE INFORMATION AND PUBLICATIONS ARISING FROM THE STUDY.

The results of this study are confidential and will be published only after the authorization of

the Principal Investigator and the sponsoring institution. It is anticipated that the results of this protocol will be presented to the scientific community through oral presentations at meetings and in written publications in scientific journals. Researchers who are not named from the beginning in this protocol will need to submit to the Investigator Assurance Agreement.

20. DEVIATIONS AND MODIFICATIONS TO THE PROTOCOL.

Inadvertent non-compliance with any section of this protocol will be reported as a deviation from the protocol and will be reported to each of the monitors and the ethics committees. Any modification to the protocol will be reported and submitted to the consideration of the Ethics Committees of each of the Institutions.

21. WITHDRAWAL OF VOLUNTEERS FROM THE STUDY.

Volunteers who participate in any of the three steps of the clinical trial may withdraw at any time from the study. A memorandum for registration will be written during the study to document volunteer departures or withdrawals. There will be a CRF to report the withdrawal of volunteers.

21.1 Follow-up of volunteers who do not continue in the study.

If a volunteer is excluded from the study for any reason after the challenge but before the infection is detected, he/she will be treated immediately according to the protocol and every effort will be made to adequately monitor the volunteer.

If the volunteer presents clinical manifestations secondary to malaria, all required efforts will be made to provide the appropriate treatment and perform the pertinent follow-up evaluations up to 1 year after the challenge. The reason for the withdrawal of any volunteer will be registered in the FRC and accompanied by a memorandum supporting such information.

22. TIMETABLE

2010 2011 2012 2013 2014 2015

ACTIVITIES	2	4	6	8	10	12	14	16	18	20	22	24	26	28	30	32	34	36	38	40	42	44	46	48	50	52	54	56	58	60	62	64	66	68	70	72
Discussion, experimental design, and writing of study protocol								■	■	■	■	■	■																							
Antigen preparation and shipment	■	■	■	■	■	■	■	■	■	■	■	■	■	■	■	■	■	■	■	■	■	■	■													
Ethics committee review and approval (CECIV)													■	■																						
Logistics (provision of facilities, staff education, and training)					■	■	■	■	■	■	■	■	■	■	■	■	■	■	■	■	■	■	■													
INVIMA review and approval																			■	■																
Optimization of the infectious challenge model.																				■	■	■	■													
IMBANACO Ethics Committee review and approval																							■													
**Pre-selection and selection of volunteers**																											■	■	■							
**Immunizations**																														■	■		■			
**Evaluation of the immune response**																																■	■	■		
**Volunteers follow-up**																																■	■	■		
**Challenge**																																		■		
**Post-challenge follow-up**																																		■	■	■
**Data Analysis**																																			■	■
**Final report**																																				■

23. REFERENCES

Alonso P.L., Sacarlal J., Aponte J.J., Leach A., Macete E., Milman J., Mandomando I., Spiessens B., Guinovart C., Espasa M., Bassat Q., Aide P., Ofori-Anyinam O., Navia M.M., Corachan S., Ceuppens M., Dubois M.C., Demoitie M.A., Dubovsky F., Menendez C., Tornieporth N., Ballou W.R., Thompson R., Cohen J. (2004) Efficacy of the RTS,S/AS02A vaccine against *Plasmodium* falciparum infection and disease in young African children: randomised controlled trial. Lancet 364:1411-20. DOI: S0140673604172231 [pii] 10.1016/S0140-6736(04)17223-1.

Arevalo-Herrera M., Herrera S. (2001) *Plasmodium vivax* malaria vaccine development. Mol Immunol 38:443-55. DOI: S0161589001000803 [pii].

Arevalo-Herrera M., Roggero M.A., Gonzalez J.M., Vergara J., Corradin G., Lopez J.A., Herrera S. (1998) Mapping and comparison of the B-cell epitopes recognized on the *Plasmodium* vivax circumsporozoite protein by immune Colombians and immunized Aotus monkeys. Ann Trop Med Parasitol 92:539-51.

Arevalo-Herrera M., Vera O., Castellanos A., Cespedes N., Soto L., Corradin G., Herrera S. (2011a) Preclinical vaccine study of *Plasmodium vivax* circumsporozoite protein derived-synthetic polypeptides formulated in montanide ISA 720 and montanide ISA 51 adjuvants. Am J Trop Med Hyg 84:21-7. DOI: 84/2_Suppl/21 [pii]10.4269/ajtmh.2011.10-0110.

Arevalo-Herrera M., Soto L., Perlaza B.L., Cespedes N., Vera O., Lenis A.M., Bonelo A., Corradin G., Herrera S. (2011b) Antibody-mediated and cellular immune responses induced in naïve volunteers by vaccination with long synthetic peptides derived from the *Plasmodium vivax* circumsporozoite protein. Am J Trop Med Hyg 84:35-42. DOI: 84/2_Suppl/35 [pii] 10.4269/ajtmh.2011.09-0507.

Arévalo-Herrera M., Chitnis C., Herrera S. (2010) Current status of *Plasmodium vivax* vaccine. Hum Vaccin 6:124-132.

Arnot D.E., Stewart M.J., Barnwell J.W. (1990) Antigenic diversity in Thai *Plasmodium vivax* circumsporozoite proteins. Mol Biochem Parasitol 43:147-9. DOI: 0166-6851(90)90140-H [pii].

Arnot D.E., Barnwell J.W., Tam J.P., Nussenzweig V., Nussenzweig R.S., Enea V. (1985) Circumsporozoite protein of *Plasmodium vivax*: gene cloning and characterization of the immunodominant epitope. Science 230:815-8.

Barnwell J.W., Galinski M.R. (1995) *Plasmodium vivax*: a glimpse into the unique and shared biology of the merozoite. Ann Trop Med Parasitol 89:113-20.

Barnwell J.W., Galinski M.R., DeSimone S.G., Perler F., Ingravallo P. (1999) *Plasmodium vivax*, P. cynomolgi, and P. knowlesi: identification of homologue proteins associated with the surface of merozoites. Exp Parasitol 91:238-49. DOI: S0014-4894(98)94372-7 [pii] 10.1006/expr.1998.4372.

Bojang K.A., Milligan P.J., Pinder M., Vigneron L., Alloueche A., Kester K.E., Ballou W.R., Conway D.J., Reece W.H., Gothard P., Yamuah L., Delchambre M., Voss G., Greenwood B.M., Hill A., McAdam K.P., Tornieporth N., Cohen J.D., Doherty T. (2001) Efficacy of RTS,S/AS02 malaria vaccine against *Plasmodium falciparum* infection in semi-immune

adult men in The Gambia: a randomised trial. Lancet 358:1927-34. DOI: S0140-6736(01)06957-4 [pii] 10.1016/S0140-6736(01)06957-4.

Breman J.G., Egan A., Keusch G.T. (2001) The intolerable burden of malaria: a new look at the numbers. Am J Trop Med Hyg 64:iv-vii.

Burkot T.R., Wirtz R.A., Paru R., Garner P., Alpers M.P. (1992) The population dynamics in mosquitoes and humans of two *Plasmodium vivax* polymorphs distinguished by different circumsporozoite protein repeat regions. Am J Trop Med Hyg 47:778-786.

Cattani J.A., Tulloch J.L., Vrbova H., Jolley D., Gibson F.D., Moir J.S., Heywood P.F., Alpers M.P., Stevenson A., Clancy R. (1986a) The epidemiology of malaria in a population surrounding Madang, Papua New Guinea. Am J Trop Med Hyg 35:3-15.

Cattani J.A., Tulloch J.L., Vrbova H., Jolley D., Gibson F.D., Moir J.S., Heywood P.F., Alpers M.P., Stevenson A., Clancy R. (1986b) The epidemiology of malaria in a population surrounding Madang, Papua New Guinea. Am J Trop Med Hyg 35:3-15.

Cerami C., Frevert U., Sinnis P., Takacs B., Clavijo P., Santos M.J., Nussenzweig V. (1992) The basolateral domain of the hepatocyte plasma membrane bears receptors for the circumsporozoite protein of *Plasmodium falciparum* sporozoites. Cell 70:1021-33. DOI: 0092-8674(92)90251-7 [pii].

Clyde D.F. (1975) Immunization of man against *falciparum* and *vivax* malaria by use of attenuated sporozoites. Am J Trop Med Hyg 24:397-401.

Clyde D.F., McCarthy V.C., Miller R.M., Hornick R.B. (1973) Specificity of protection of man immunized against sporozoite-induced *falciparum* malaria. Am J Med Sci 266:398-403.

Cochrane A.H., Aikawa M., Jeng M., Nussenzweig R.S. (1976) Antibody-induced ultrastructural changes of malarial sporozoites. Journal of Immunology 116:859-67.

Cochrane A.H., Nardin E.H., de Arruda M., Maracic M., Clavijo P., Collins W.E., Nussenzweig R.S. (1990) Widespread reactivity of human sera with a variant repeat of the circumsporozoite protein of *Plasmodium vivax*. Am J Trop Med Hyg 43:446-51.

Collins W.E., Jeffery G.M. (1999) A retrospective examination of sporozoite- and trophozoite-induced infections with *Plasmodium falciparum* in patients previously infected with heterologous species of *Plasmodium*: effect on development of parasitologic and clinical immunity. Am J Trop Med Hyg 61:36-43.

Collins W.E., Sullivan J.S., Morris C.L., Galland G.G., Richardson B.B. (1996) Observations on the biological nature of *Plasmodium vivax* sporozoites. J Parasitol 82:216-9.

Comer R.D., Young M.D., Porter J.A., Jr., Gauld J.R., Merritt W. (1968) Chloroquine resistance in *Plasmodium falciparum* malaria on the Pacific coast of Colombia. Am J Trop Med Hyg 17:795-9.

Charoenvit Y., Collins W.E., Jones T.R., Millet P., Yuan L., Campbell G.H., Beaudoin R.L., Broderson J.R., Hoffman S.L. (1991) Inability of malaria vaccine to induce antibodies to a protective epitope within its sequence. Science 251:668-71.

Chitnis C.E. (2001) Molecular insights into receptors used by malaria parasites for erythrocyte invasion. Curr Opin Hematol 8:85-91.

Chulay J.D., Schneider I., Cosgriff T.M., Hoffman S.L., Ballou W.R., Quakyi I.A., Carter R., Trosper J.H., Hockmeyer W.T. (1986) Malaria transmitted to humans by mosquitoes infected from cultured *Plasmodium falciparum*. Am J Trop Med Hyg 35:66-8.

del Portillo H.A., Longacre S., Khouri E., David P.H. (1991) Primary structure of the merozoite surface antigen 1 of *Plasmodium vivax* reveals sequences conserved between different *Plasmodium* species. Proc Natl Acad Sci U S A 88:4030-4.

Druihle P., Reina L., Fidock D.A. (1998) Immunity to liver stages. , in: I. Sherman (Ed.), Malaria: Parasite biology, pathogenesis and protection Washington DC. pp. 513-543.

Egan J.E., Hoffman S.L., Haynes J.D., Sadoff J.C., Schneider I., Grau G.E., Hollingdale M.R., Ballou W.R., Gordon D.M. (1993) Humoral immune responses in volunteers immunized with irradiated *Plasmodium falciparum* sporozoites. Am J Trop Med Hyg 49:166-73.

Eyles D.E. (1950) A stain for malarial oocysts in temporary preparations. J Parasitol 36:501.

Fairley N.H. (1947) Sidelights on malaria in man obtained by subinoculation experiments. Trans R Soc Trop Med Hyg 40:621-76.

Franke E.D., Lucas C.M., San Roman E., Wirtz R.A. (1992a) Prevalence of antibody to the variant repeat of the circumsporozoite protein of *Plasmodium vivax* in Peru. Am J Trop Med Hyg 46:708-10.

Franke E.D., Lucas C.M., Chauca G., Wirtz R.A., Hinostroza S. (1992b) Antibody response to the circumsporozoite protein of *Plasmodium vivax* in naturally infected humans. Am J Trop Med Hyg 46:320-6.

Frevert U., Sinnis P., Cerami C., Shreffler W., Takacs B., Nussenzweig V. (1993) Malaria circumsporozoite protein binds to heparan sulfate proteoglycans associated with the surface membrane of hepatocytes. J. Exp. Med. 177:1287-1298.

Galinski M.R., Corredor-Medina C., Povoa M., Crosby J., Ingravallo P., Barnwell J.W. (1999) *Plasmodium vivax* merozoite surface protein-3 contains coiled-coil motifs in an alanine-rich central domain. Mol Biochem Parasitol 101:131-47. DOI: S0166-6851(99)00063-8 [pii].

Galinski M.R., Ingravallo P., Corredor-Medina C., Al-Khedery B., Povoa M., Barnwell J.W. (2001) *Plasmodium vivax* merozoite surface proteins-3beta and-3gamma share structural similarities with *P. vivax* merozoite surface protein-3alpha and define a new gene family. Mol Biochem Parasitol 115:41- 53. DOI: S0166-6851(01)00267-5 [pii].

Genton B., Corradin G. (2002) Malaria vaccines: from the laboratory to the field. Curr Drug Targets Immune Endocr Metabol Disord 2:255-67.

Glynn J.R., Collins W.E., Jeffery G.M., Bradley D.J. (1995) Infecting dose and severity of falciparum malaria. Trans R Soc Trop Med Hyg 89:281-3.

Gonzalez J.M., Hurtado S., Arevalo-Herrera M., Herrera S. (2001) Variants of the *Plasmodium vivax* circumsporozoite protein (VK210 and VK247) in Colombian isolates. Mem Inst Oswaldo Cruz 96:709- 12. DOI: S0074-02762001000500023 [pii].

Grassi B.A., Bignami, Bastianelli G. (1899) Ulteriori ricerche sul ciclo dei parassiti malarici umani nel corpo del zanzarone. Atti Reale Accad. Lincei 5:8-21.

Guerra C.A., Howes R.E., Patil A.P., Gething P.W., Van Boeckel T.P., Temperley W.H., Kabaria C.W., Tatem A.J., Manh B.H., Elyazar I.R., Baird J.K., Snow R.W., Hay S.I. (2010) The international limits and population at risk of *Plasmodium vivax* transmission in 2009. PLoS Negl Trop Dis 4:e774. DOI: 10.1371/journal.pntd.0000774.

Guinovart C., Aponte J.J., Sacarlal J., Aide P., Leach A., Bassat Q., Macete E., Dobano C., Lievens M., Loucq C., Ballou W.R., Cohen J., Alonso P.L. (2009) Insights into long-

lasting protection induced by RTS,S/AS02A malaria vaccine: further results from a phase IIb trial in Mozambican children. PLoS One 4:e5165. DOI: 10.1371/journal.pone.0005165.

Gunewardena D.M., Carter R., Mendis K.N. (1994) Patterns of acquired anti-malarial immunity in Sri Lanka. Mem Inst Oswaldo Cruz 89:63-5.

Gysin J., Moisson P., Pereira da Silva L., Druilhe P. (1996) Antibodies from immune African donors with a protective effect in *Plasmodium falciparum* human infection are also able to control asexual blood forms of the parasite in Saimiri monkeys. Res Immunol 147:397-401. DOI: 0923249496820487 [pii].

Herrera M.A., de Plata C., Gonzalez J.M., Corradin G., Herrera S. (1994) Immunogenicity of multiple antigen peptides containing *Plasmodium vivax* CS epitopes in BALB/c mice. Mem Inst Oswaldo Cruz 89:71-6.

Herrera S., De Plata C., Gonzalez M., Perlaza B.L., Bettens F., Corradin G., Arevalo-Herrera M. (1997) Antigenicity and immunogenicity of multiple antigen peptides (MAP) containing *P. vivax* CS epitopes in Aotus monkeys. Parasite Immunol 19:161-70.

Herrera S., Fernandez O.L., Vera O., Cardenas W., Ramirez O., Palacios R., Chen-Mok M., Corradin G., Arevalo-Herrera M. (2011a) Phase I safety and immunogenicity trial of *Plasmodium vivax* CS derived long synthetic peptides adjuvanted with montanide ISA 720 or montanide ISA 51. Am J Trop Med Hyg 84:12-20. DOI: 84/2_Suppl/12 [pii] 10.4269/ajtmh.2011.09-0516.

Herrera S., Solarte Y., Jordan-Villegas A., Echavarria J.F., Rocha L., Palacios R., Ramirez O., Velez J.D., Epstein J.E., Richie T.L., Arevalo-Herrera M. (2011b) Consistent safety and infectivity in sporozoite challenge model of *Plasmodium vivax* in malaria-naïve human volunteers. Am J Trop Med Hyg 84:4- 11. DOI: 84/2_Suppl/4 [pii] 10.4269/ajtmh.2011.09-0498.

Herrera S., Fernández O., Manzano M.R., Murrain B., Vergara J., Blanco P., Palacios R., Vélez J.D., Epstein J.E., Chen-Mok M., Reed Z.H., Arévalo-Herrera M. (2009a) Successful Sporozoite Challenge Model in Human Volunteers with *Plasmodium vivax* Strain Derived from Human Donor. Am J Trop Med Hyg Accepted.

Herrera S., Solarte Y., Parra J., Jordan A., Echavarria J.F., Rocha L., Palacios R., Ramirez O., Velez J.D., Epstein J.E., Richie T., Arevalo-Herrera M. (2009b) Reproducibility of a sporozoite challenge model for *Plasmodium vivax* in human naïve volunteers. Am J Trop Med Hyg aceptted.

Herrera S., Fernandez O., Manzano M.R., Murrain B., Vergara J., Blanco P., Palacios R., Velez J.D., Epstein J.E., Chen-Mok M., Reed Z.H., Arevalo-Herrera M. (2009c) Successful sporozoite challenge model in human volunteers with *Plasmodium vivax* strain derived from human donors. Am J Trop Med Hyg 81:740-6. DOI: 81/5/740 [pii] 10.4269/ajtmh.2009.09-0194.

Herrera S., Bonelo A., Perlaza B.L., Fernandez O.L., Victoria L., Lenis A.M., Soto L., Hurtado H., Acuna L.M., Velez J.D., Palacios R., Chen-Mok M., Corradin G., Arevalo-Herrera M. (2005) Safety and elicitation of humoral and cellular responses in colombian malaria-naïve volunteers by a *Plasmodium vivax* circumsporozoite protein-derived synthetic vaccine. Am J Trop Med Hyg 73:3-9. DOI: 73/5_suppl/3 [pii].

Herrington D.A., Nardin E.H., Losonsky G., Bathurst I.C., Barr P.J., Hollingdale M.R., Edelman R., Levine M.M. (1991) Safety and immunogenicity of a recombinant sporozoite malaria vaccine against *Plasmodium vivax*. Am J Trop Med Hyg 45:695-701.

Hoffman S.L., Doolan D.L. (2000) Malaria vaccines-targeting infected hepatocytes. Nat Med 6:1218- 9. DOI: 10.1038/81315.

Hoffman S.L., Goh L.M., Luke T.C., Schneider I., Le T.P., Doolan D.L., Sacci J., de la Vega P., Dowler M., Paul C., Gordon D.M., Stoute J.A., Church L.W., Sedegah M., Heppner D.G., Ballou W.R., Richie T.L. (2002) Protection of humans against malaria by immunization with radiation-attenuated *Plasmodium falciparum* sporozoites. J Infect Dis 185:1155-64. DOI: JID010922 [pii] 10.1086/339409.

Hurtado S., Salas M.L., Romero J.F., Zapata J.C., Ortiz H., Arevalo-Herrera M., Herrera S. (1997) Regular production of infective sporozoites of *Plasmodium falciparum* and *P. vivax* in laboratory- bred Anopheles albimanus. Ann Trop Med Parasitol 91:49-60.

Kain K.C., Brown A.E., Webster H.K., Wirtz R.A., Keystone J.S., Rodriguez M.H., Kinahan J., Rowland M., Lanar D.E. (1992) Circumsporozoite genotyping of global isolates of *Plasmodium vivax* from dried blood specimens. J Clin Microbiol 30:1863-6.

Kumar S., Epstein J.E., Richie T.L., Nkrumah F.K., Soisson L., Carucci D.J., Hoffman S.L. (2002) A multilateral effort to develop DNA vaccines against falciparum malaria. Trends Parasitol 18:129-35. DOI: S1471492201022073 [pii].

Macete E., Aponte J.J., Guinovart C., Sacarlal J., Ofori-Anyinam O., Mandomando I., Espasa M., Bevilacqua C., Leach A., Dubois M.C., Heppner D.G., Tello L., Milman J., Cohen J., Dubovsky F., Tornieporth N., Thompson R., Alonso P.L. (2007) Safety and immunogenicity of the RTS,S/AS02A candidate malaria vaccine in children aged 1-4 in Mozambique. Trop Med Int Health 12:37-46. DOI: TMI1754 [pii] 10.1111/j.1365-3156.2006.01754.x.

Machado R.L., Povoa M.M. (2000) Distribution of *Plasmodium vivax* variants (VK210, VK247 and P. vivax-like) in three endemic areas of the Amazon region of Brazil and their correlation with chloroquine treatment. Trans R Soc Trop Med Hyg 94:377-81.

Maheswary N.P., Perpanich B., Rosenberg R. (1992) Presence of antibody to a heterologous circumsporozoite protein of *Plasmodium vivax* (VK247) in southeastern Bangladesh. Trans R Soc Trop Med Hyg 86:28.

Mann V.H., Huang T., Cheng Q., Saul A. (1994) Sequence variation in the circumsporozoite protein gene of *Plasmodium vivax* appears to be regionally biased. Mol Biochem Parasitol 68:45-52. DOI: 0166-6851(94)00148-0 [pii].

Mendis K., Sina B.J., Marchesini P., Carter R. (2001) The neglected burden of *Plasmodium vivax* malaria. Am J Trop Med Hyg 64:97-106.

Miller L.H., McAuliffe F.M., Mason S.J. (1977) Erythrocyte receptors for malaria merozoites. Am J Trop Med Hyg 26:204-8.

Mourya, D. T., Gokhale, M. D., & Kumar, R. (2007). Xenodiagnosis: use of mosquitoes for the diagnosis of arboviral infections. *Journal of vector borne diseases*, *44*(4), 233.

Nardin E., Clavijo P., Mons B., van Belkum A., Ponnudurai T., Nussenzweig R.S. (1991) T cell epitopes of the circumsporozoite protein of *Plasmodium vivax*. Recognition by

lymphocytes of a sporozoite- immunized chimpanzee. J Immunol 146:1674-8.

Nussenzweig R., Vanderberg J., Most H. (1969) Protective immunity produced by the injection of x- irradiated sporozoites of *Plasmodium berghei*. IV. Dose response, specificity and humoral immunity. Mil Med 134:1176-82.

Qari S.H., Goldman I.F., Povoa M.M., di Santi S., Alpers M.P., Lal A.A. (1992) Polymorphism in the circumsporozoite protein of the human malaria parasite *Plasmodium vivax*. Mol. Biochem. Parasitol. 55:105-114.

Ramasamy R., Nagendran K., Ramasamy M.S. (1994) Antibodies to epitopes on merozoite and sporozoite surface antigens as serologic markers of malaria transmission: studies at a site in the dry zone of Sri Lanka. Am J Trop Med Hyg 50:537-47.

Richie T.L., Saul A. (2002) Progress and challenges for malaria vaccines. Nature 415:694-701. DOI: 10.1038/415694a415694a [pii].

Rieckmann K.H., Beaudoin R.L., Cassells J.S., Sell K.W. (1979) Use of attenuated sporozoites in the immunization of human volunteers against falciparum malaria. Bull World Health Organ 57:261-5.

Rodriguez M.H., Betanzos-Reyes A.F., Hernandez-Avila J.E., Mendez-Galvan J.F., Danis-Lozano R., Altamirano-Jimenez A. (2009) The participation of secondary clinical episodes in the epidemiology of *vivax* malaria during pre- and post-implementation of focal control in the state of Oaxaca, Mexico. Am J Trop Med Hyg 80:889-95. DOI: 80/6/889 [pii].

Rogers W.O., Malik A., Mellouk S., Nakamura K., Rogers M.D., Szarfman A., Gordon D.M., Nussler A.K., Aikawa M., Hoffman S.L. (1992) Characterization of *Plasmodium falciparum* sporozoite surface protein 2. Proc Natl Acad Sci U S A 89:9176-80.

Rosenberg R., Wirtz R.A., Lanar D.E., Sattabongkot J., Hall T., Waters A.P., Prasittisuk C. (1989) Circumsporozoite protein heterogeneity in the human malaria parasite *Plasmodium vivax*. Science 245:973-6.

Rougemont M., Van Saanen M., Sahli R., Hinrikson H.P., Bille J., Jaton K. (2004) Detection of four *Plasmodium* species in blood from humans by 18S rRNA gene subunit-based and species-specific real-time PCR assays. J Clin Microbiol 42:5636-43. DOI: 42/12/5636 [pii] 10.1128/JCM.42.12.5636-5643.2004.

Sachs J., Malaney P. (2002) The economic and social burden of malaria. Nature 415:680-5. DOI: 10.1038/415680a 415680a [pii].

Sattabongkot J., Tsuboi T., Zollner G.E., Sirichaisinthop J., Cui L. (2004) *Plasmodium vivax* transmission: chances for control? Trends Parasitol 20:192-8. DOI: 10.1016/j.pt.2004.02.001 S1471492204000327 [pii].

Sinnis P., Nussenzweig V. (1996) Preventing sporozoite invasion of hepatocytes, in: S. L. Hoffman (Ed.), Malaria vaccine development. A multi-immune response approach, ASM Press, Washington, D.C. pp. 15-34.

Solarte Y., Manzano M.R., Rocha L., Hurtado H., James M.A., Arévalo-Herrera M., Herrera S. (2011) *Plasmodium vivax* sporozoite production in Anopheles albimanus mosquitoes for vaccine clinical trials. The American journal of tropical medicine and hygiene 84:28-34.

Soto J., Toledo J., Gutierrez P., Luzz M., Llinas N., Cedeno N., Dunne M., Berman J. (2001) *Plasmodium vivax* clinically resistant to chloroquine in Colombia. Am J Trop Med Hyg

65:90-3.

Stoute J.A., Slaoui M., Heppner D.G., Momin P., Kester K.E., Desmons P., Wellde B.T., Garcon N., Krzych U., Marchand M. (1997) A preliminary evaluation of a recombinant circumsporozoite protein vaccine against *Plasmodium falciparum* malaria. RTS,S Malaria Vaccine Evaluation Group. N Engl J Med 336:86-91. DOI: 10.1056/NEJM199701093360202.

Stoute J.A., Heppner D.G., Jr., Mason C.J., Siangla J., Opollo M.O., Kester K.E., Vigneron L., Voss G., Walter M.J., Tornieporth N., Cohen J.D., Ballou W.R. (2006) Phase 1 safety and immunogenicity trial of malaria vaccine RTS,S/AS02A in adults in a hyperendemic region of western Kenya. Am J Trop Med Hyg 75:166-70. DOI: 75/1/166 [pii].

Stoute J.A., Kester K.E., Krzych U., Wellde B.T., Hall T., White K., Glenn G., Ockenhouse C.F., Garcon N., Schwenk R., Lanar D.E., Sun P., Momin P., Wirtz R.A., Golenda C., Slaoui M., Wortmann G., Holland C., Dowler M., Cohen J., Ballou W.R. (1998) Long-term efficacy and immune responses following immunization with the RTS,S malaria vaccine. J Infect Dis 178:1139-44.

Templeton T.J., Kaslow D.C. (1997) Cloning and cross-species comparison of the thrombospondin- related anonymous protein (TRAP) gene from *Plasmodium knowlesi, Plasmodium vivax* and *Plasmodium gallinaceum*. Mol Biochem Parasitol 84:13-24. DOI: S0166-6851(96)02775-2 [pii].

Thomas A.W., Trape J.F., Rogier C., Goncalves A., Rosario V.E., Narum D.L. (1994) High prevalence of natural antibodies against *Plasmodium falciparum* 83-kilodalton apical membrane antigen (PF83/AMA-1) as detected by capture-enzyme-linked immunosorbent assay using full-length baculovirus recombinant PF83/AMA-1. Am J Trop Med Hyg 51:730-40.

Tsuji M., Zavala F. (2001) Peptide-based subunit vaccines against pre-erythrocytic stages of malaria parasites. Mol Immunol 38:433-42. DOI: S0161589001000797 [pii].

Walther M., Dunachie S., Keating S., Vuola J.M., Berthoud T., Schmidt A., Maier C., Andrews L., Andersen R.F., Gilbert S., Poulton I., Webster D., Dubovsky F., Tierney E., Sarpotdar P., Correa S., Huntcooke A., Butcher G., Williams J., Sinden R.E., Thornton G.B., Hill A.V. (2005) Safety, immunogenicity and efficacy of a pre-erythrocytic malaria candidate vaccine, ICC-1132 formulated in Seppic ISA 720. Vaccine 23:857-64. DOI: S0264-410X(04)00642-5 [pii] 10.1016/j.vaccine.2004.08.020.

WHO. (2010) World malaria report 2010, World Health Organization, Washington.

Williams T.N., Maitland K., Bennett S., Ganczakowski M., Peto T.E., Newbold C.I., Bowden D.K., Weatherall D.J., Clegg J.B. (1996) High incidence of malaria in alpha-thalassaemic children. Nature 383:522-5. DOI: 10.1038/383522a0.

Wirtz R.A., Burkot T.R., Graves P.M., Andre R.G. (1987) Field evaluation of enzyme-linked immunosorbent assays for *Plasmodium falciparum* and *Plasmodium vivax* sporozoites in mosquitoes (Diptera: Culicidae) from Papua New Guinea. J Med Entomol 24:433-7.

Wirtz R.A., Rosenberg R., Sattabongkot J., Webster H.K. (1990) Prevalence of antibody to heterologous circumsporozoite protein of *Plasmodium vivax* in Thailand. Lancet 336:593-5. DOI: 0140-6736(90)93393-4 [pii].

Wombou Toukam CM, Solano P, Bengaly Z, Jamonneau V, Bucheton B. (2011) Experimental

evaluation of xenodiagnosis to detect trypanosomes at low parasitaemia levels in infected hosts. Parasite 18(4):295-302

Yadava A., Sattabongkot J., Washington M.A., Ware L.A., Majam V., Zheng H., Kumar S., Ockenhouse C.F. (2007) A novel chimeric *Plasmodium vivax* circumsporozoite protein induces biologically functional antibodies that recognize both VK210 and VK247 sporozoites. Infect Immun 75:1177-85. DOI: IAI.01667-06 [pii] 10.1128/IAI.01667-06.

ANNEX 1

PROCEDURE FOR THE *P. vivax* CS PROTEIN VACCINE FORMULATION

Peptides N, R and C have been packaged in 5 mL vials in quantities of 120 µg for each peptide, in two presentations:

- Mixture 1. N + C peptides (120 µg of each, total: 240 µg of protein).
- Mixture 2. N + R + C peptides (120 µg of each, total: 360 µg of protein).

Each group will receive a 50 µg dose of each of the N and C peptides, in the first injection (mix 1); and 50 µg of peptides N, R and C in the second and third injections (mix 2).

Note: Each presentation corresponds to the dose for two volunteers

First injection preparation.

For the first injection, 8 vials of 240 µg of protein will be used. Each 240 µg vial will be dissolved in 500 µL of distilled water and mixed with 500 µL of Montanide ISA-51 (Seppic, France) for a final volume of 1000 µL. Mix 20x with a 10mL glass syringe, the total (final) volume. The mixtures will be injected within the next 5 hours.

Second and third injection preparation.

For the second and third injection, 16 vials of 360 µg of protein will be used. Each 360 µg vial will be dissolved in 500 µL of distilled water and mixed with 500 µL of Montanide ISA-51 (Seppic, France) for a final volume of 1000 µL. Mix 20x with a 10mL glass syringe, the total (final) volume. The mixtures will be injected within the next 5 hours.

Montanide preparation for the control group.

500 µL of distilled water and 500 µL of Montanide ISA-51will be taken for a final volume of 1000 µL.

Each preparation will be packaged in 1 mL syringes with a 21G short needle, and 500 µL per volunteer will be injected intramuscularly into the left deltoid.

A loss of approximately 20% of each peptide is considered during this procedure. For this reason the presentation of the products is 240 and 360 µg to ensure a dose of 100 for the first injection and 150 µg for the second and third per volunteer.

Randomized clinical trial to assess the protective efficacy of a *Plasmodium vivax* CS synthetic vaccine

Supplemental Material

Supplement Note 2

Paraclinical Safety Tests

Safety paraclinical tests were taken at the time of recruitment (selection), at the first immunization (month 0), after the first immunization (month 1), before the second (month 2), after the second immunization (month 3), before the third (month 6) and after the third (pre-challenge).

Paraclinical safety alterations during immunizations totaled 191 and consisted of: Anemia 24% (46), hematuria 15% (29), hyperglycemia 12% (23), proteinuria 9% (17), prolonged partial thromboplastin time 8 % (16), eosinophilia 7% (14), elevation of glutamic pyruvic transaminase 7.3% (14), prolongation of thrombin times 6% (11), elevation of indirect bilirubin without alteration of the AST/ALT pattern 2% (4), glutamic oxaloacetic transaminase elevation 1% (2), Leukocytopenia 3% (6), Leukocytosis 2% (4), Neutropenia 2% (4).

**Indirect bilirubin up to 0.83mg / dL**				
	**CS1018**	**CS1025**	**CS1030**	**CS1535**
Selection	0.08	0.13	0.28	0.28
Lab Control Month 0	0.34	0.10	0.88	0.04
Lab Control Month 1	0.02	0.06	0.2	0.22
Lab Control Month 2	0.1	0.86	0.12	0.13
Lab Control Month 3	0.16	0.05	0.58	0.1
Lab Control Month 6	0.9	0.10	0.11	0.88
Lab Control Pre- challenge	0.11	0.08	0.02	0.01

Table 4. Indirect bilirubin in the paraclinical follow-up of immunized volunteers who presented an adverse event (AE)

Renal function:
There was no alteration in renal function: creatinine and urea nitrogen values. All values were within the Protocol's standard ranges.
Glycemia:
There was a glycemia elevation in 17 volunteers in the selection paraclinical and on the first day of immunization. However, despite the request to present fasting for volunteers, some paraclinical were not taken on an empty stomach. Volunteers took their glycemia levels when they had time disposition. To determine glycemia, it was pertinent to measure fasting glucose levels or with a glucose tolerance curve.

	**Selection**	**Grade**	**Lab Control Month 0**	**Grade**
**CS1001**	121	1		
**CS1003**	104		126	2
**CS1015**	123	1	99	
**CS1018**	113	1	110	1
**CS1036**	110	1		

**CS1037**	110	1		
**CS1506**	111	1	117	1
**CS1535**	87		156	2
**CS1537**	77		124	1
**CS1538**	94		152	2
**CS1547**	95		119	1
**CS1549**	97		130	2
**CS1553**	87		112	1
**CS1554**	90		121	1
**CS1565**	111	1	119	1
**CS1570**	101		125	1
**CS1572**	92		113	1
**CS1575**	97		112	1
**CS1581**	110	1	110	1

Table 5. Glycemia in the paraclinical follow-up of immunized volunteers who presented an AE.

<u>Transaminases:</u>
Grade 1 transaminase elevation (ALT and AST) (elevation 1.1-2.5 ULN) occurred in 7 volunteers (CS1575, CS1025, CS1030, CS1031, CS1538, CS1570, CS1581).
Elevated Glutamic Pyruvic Transaminase:
Grade 1 elevation (elevation 1.1-2.5 ULN) occurred in 7 volunteers. Bearing in mind that the reference values vary according to whether you are male or female. In men's case, the reference value is Up to 40 U / L; therefore, a Grade 1 AE corresponds to an elevation greater than 44 U / L in the case of men (CS 1575). In the case of women, the reference value is up to 32 U / L. Therefore, AE Grade 1 corresponds to values higher than 35 (CS1025, CS1031, CS1538, CS1570, CS1581). Altered values at the time of selection are not related to immunizations due to temporality; subsequent elevations may or may not be related.
It should be noted that the volunteer CS1565 and CS1538 presented elevations in glutamic pyruvic transaminase from the beginning of the study before the administration of the immunization. Both belong to the experimental group. Volunteers CS1025, CS1030, CS1031, CS1575, CS1581 were part of the experimental group. Volunteer CS1570 belonged to the control group.

**Glutamic Pyruvic Transaminase U / L**								
Moment	**CS1025**	**CS1031**	**CS1538**	**CS1565**	**CS1570**	**CS1575**	**CS1581**	**Grado**
Selection	31	31	39	39	26	29	28	1
Lab Control Month 0	33	24	50	25	13	40	28	1

Lab Control Month 1	54	22	61	22	20	41	23	1
Lab Control Month 2	22	48	53	19	15	41	36	1
Lab Control Month 3	25	31	59	29	12	27	27	1
Lab Control Month 6	38	52	19	23	28	80	19	1
Lab Control pre-challenge	26	14	72	31	53	37	15	1

Table 6. Glutamic Pyruvic transaminase in the paraclinical follow-up of immunized volunteers who presented an AE.

Elevation of glutamic oxaloacetic transaminase:

There was no evidence of elevated SGOT in any male volunteers from the moment of recruitment until the safety paraclinical after the third immunization.

Volunteer CS1538, who belonged to the experimental group, presented Grade 1 elevation of the SGOT in control paraclinical after the first and third immunization.

**Glutamic Oxaloacetic Transaminase (SGOT) Woman Reference value: 8-39 U/L**		
	**CS1538**	**Grade**
Selection	34	1
Lab Control Month 0	40	1
Lab Control Month 1	51	1
Lab Control Month 2	40	1
Lab Control Month 3	43	1
Lab Control Month 6	23	1
Labo Control pre-challenge	46	1

Table 7. Glutamic Oxalacetic Transaminase in the paraclinical follow-up of immunized volunteers who presented an AE.

Clotting times:

Prothrombin time: (standard value 12-15 sec)

There was a slight prolongation considering a Grade I adverse event in 9 volunteers, of which eight corresponded to the experimental group (CS1006, CS1015, CS1028, CS1031,

CS1036, CS1506, CS1565, CS1575) and 1 to the control group (CS1037).
However, two volunteers who belong to the experimental group and one who belongs to the control group presented prolonged selection paraclinical.

**PT VN 12-15 sec**	**CS1006**	**CS1015**	**CS1028**	**CS1031**	**CS1036**	**CS1037**	**CS1506**	**CS1565**	**CS1575**
Selection	8.3	9.5	13.7	8.4	13.6	14.2	9.7	8.3	9.6
Lab Control Month 0	8.2	9.3	9.1	8.4	8.2	8.70	9.5	8.6	8.3
Lab Control Month 1	9.8	9.2	8.7	10.2	8.5	10.3	8.2	9.2	9.3
Lab Control Month 2	8.6	10.8	10.4	9.8	10.5	8.3	11.2	9.4	10.0
Lab Control Month 3	11.0	9.8	9.9	11.1	9.8	9.5	9.5	11.0	11.0
Lab Control Month 6	8.7	9.9	9.2	8.5	8.5	9.8	9.0	11.1	8.9
Lab Control pre-challenge	9.8	9.0	9.3	8.2	9.8	9.6	11.8	10.2	7.5
**Grade**	1	1	4	1	4	4	1	1	1

Table 8. Prothrombin time in the paraclinical follow-up of immunized volunteers who presented an AE.

Partial Thromboplastin Time (PTT):
(normal value 25-35 sec)
There was an alteration in the partial thromboplastin time values, following the Protocol's adverse event values in 14 volunteers.
10 volunteers (CS1023, CS1028, CS1031, CS1038, CS1506, CS1511, CS1537, CS1547, CS1553, CS1565) belonged to the experimental group 4 volunteers (CS1037, CS1549, CS1554, CS1574).
However, 4 volunteers of the 14 who presented prolongation of the TTP showed this alteration in the selection paraclinical. Of these three volunteers correspond to the experimental group and one to the control group.

PTT VN 25-35 sec	CS1023	CS1028	CS1031	CS1037	CS1038	CS1506	CS1511	CS1537	CS1547	CS1549	CS1553	CS1565	CS1574
**Selection**	31.1	36.5	31.8	36.7	22.3	24.8	22.9	30.2	26.8	28.3	25.9	28.4	25.3
**Lab Control Month 0**	24.8	25.3	28.6	22.70	22.80	28.8	22.3	28.2	27.4	24.8	23.0	23.7	25.8
**Lab Control Month 1**	26.9	23.3	28.6	26.7	26.1	24.3	26.7	23.6	23.6	22.2	24.2	27.1	28.1
**Lab Control Month 2**	25.3	29.9	26.2	26.6	27.1	27.2	26.5	25.3	27.7	28.1	22.0	26.1	18.2
**Lab Control Month 3**	23.1	25.4	27.1	26.2	27.1	25.1	26.1	26.1	26.1	22.1	23.2	24.5	22.8
**Lab Control Month 6**	25.3	27.9	30.1	25.5	32.0	31.0	27.9	26.5	30.7	35.7	28.1	25.1	27.7
**Lab Control pre-challenge**	22.8	24.6	22.3	24.9	28.5	31.9	31.8	34.5	37.7	39.3	34.0	32.9	32.9
**Grade**	1	1	1	1	1	1	1	1	1	1	1	1	1
**Grade**						1			2	2			

Table 9. Partial thromboplastin time in the paraclinical follow-up of immunized volunteers who presented an AE.

<u>Hemogram:</u>

Anemia:

The Protocol's reference values to categorize anemia are Hb less than or equal to 12g / dL in women and less than or equal to 13.5g / dL in men.

Thirteen volunteers presented a slightly low hemoglobin value ranging from 11.2 - 11.8 g / dL in 10 women and three men. Among 9 volunteers (CS1005, CS1012, CS1013, CS1028, CS1511, CS1537, CS1549, CS1574, CS1581), 10 presented Grade 1 classification and only 2 volunteers were found in Grade 2. Among the men, only one volunteer presented Grade I anemia (CS1572).

Anemia													
	**CS1005**	**CS1006**	**CS1012**	**CS1013**	**CS1028**	**CS1511**	**CS1537**	**CS1547**	**CS1549**	**CS1572**	**CS1575**	**CS1574**	**CS1581**
Selection	12.5	12.50	12.00	10.80	12.30	11.50	12.70	14.70	12.30	12.90	13.70	12.80	12.10
Lab Control Month 0	12.70	12.60	11.20	11.80		11.80	12.20	14.50	12.10	13.20	14.00	12.20	12.10
Lab Control Month 1	13.20	12.40	11.70	11.60	12.40	11.40	11.60	13.50	11.30	12.80	14.10	12.20	10.40
Lab Control Month 2	12.80	12.50	11.10	11.50	11.60	11.20	12.10	14.20	10.60	13.30	13.70	12.00	11.20
Lab Control Month 3	11.30	12.00	11.20	12.60	13.00	11.10	12.30	14.90	11.50	12.10	16.10	12.20	11.30
Lab Control Month 6	12.50	12.40	11.50	11.90	11.60	11.60	12.00	14.20	10.70	12.80	13.30	11.90	11.50
Lab Control pre-challenge	13.00	13.20	11.80	13.10	12.60	11.30	11.90	14.50	12.10	13.10	14.00	12.00	12.30
**Grade**	1	1	1	2	1	1	1	1	2	1	1	1	2

Table 10. Hemoglobin in the paraclinical follow-up of immunized volunteers who presented an AE

Thrombocytopenia:

Thrombocytopenia was not evidenced in the volunteers during the immunizations, considering the reference values of the Protocol.

Leukocytopenia:

He had Grade 1 leukopenia (3500-2500 cells / mm3), 3 volunteers (CS1006, CS1013 and CS1025). Volunteer CS1006 and CS1013 belonged to the experimental group.

**Leukocytopenia (Reference value: <3500)**				
	**CS1006**	**CS1013**	**CS1025**	
Selection	7.40	4.30	5.10	**Grade**
Lab Control Month 0	3.40	7.10	4.80	1
Lab Control Month 1	3.60	3.30	5.30	1
Lab Control Month 2	3.20	6.40	6.20	1
Lab Control Month 3	4.00	4.40	3.50	1
Lab Control Month 6	3.10	10.60	7.00	1
Lab Control pre-challenge	2.80	4.30	5.30	1

Table 11. Leukopenia in the paraclinical follow-up of immunized volunteers who presented an AE

Leukocytosis:

Three volunteers presented Grade I leukocytosis at four times, following the reference values of the Protocol. Volunteers CS1015, CS1535 and CS1537 belonged to the experimental group.

**Leukocytosis (Reference values >10.800)**				
	**CS1015**	**CS1535**	**CS1537**	**Grade**
Selection	5.5	12.10	9.4	1
Lab Control Month 0	7.20	11.40	11.80	1
Lab Control Month 1	10.90	8.90	6.70	1

Lab Control Month 2	5.60	8.00	5.80	
Lab Control Month 3	4.60	8.60	6.50	
Lab Control Month 6	7.00	10.30	8.20	
Lab Control pre-challenge	4.70	7.90	8.70	

Table 12. Leukocytosis in the paraclinical follow-up of immunized volunteers who presented an AE

Lymphopenia:

Lymphocyte levels below the ranges determined by the Protocol that characterize lymphopenia were not evidenced in volunteers during immunizations.

Neutropenia:

Three volunteers from the experimental group had mild neutropenia (CS1013, CS1006, CS1025)

Time	CS1006	CS1013	CS1025
Selection	6.00	2.40	2.50
Lab Control Month 0	1.80	4.50	2.00
Lab Control Month 1	1.90	1.20	
Lab Control Month 2	1.10	3.90	
Lab Control Month 3	1.90	2.60	1.30
Lab Control Month 6	1.60	7.50	
Lab Control pre-challenge	1.10	2.70	3.60
**Grade**	1	1	1

Table 13. Neutropenia in the paraclinical follow-up of immunized volunteers who presented an AE

Eosinophilia:

Within the reported values, five volunteers had Grade I eosinophilia, and one volunteer had Grade II eosinophilia at four times. Four volunteers belonged to the control group (CS1018, CS1554, CS1572, CS1574) and 5 to the experimental group (CS1023, CS1511, CS1535, CS1538, CS1569).

	CS1018	CS1023	CS1511	CS1535	CS1538	CS1554	CS1569	CS1572	CS1574
Selection	**0**	728,0	**0**	1089,0	888,0	344,0	288,0	882,0	768,0
Lab Control Month 0	1332,0	0	84,0	228,0	288,0	87,00	110,0	448,0	2765,0
Lab Control Month 1	602,0	0	0,0	1157,0	0	0	116,0	171,0	2160,0
Lab Control Month 2	0	0	249,0	560,0	0	552,0	0	58,0	2263,0
Lab Control Month 3	62,0	210,0	0,0	258,0	0	345,0	348,0	0	1932,0
Lab Control Month 6	0	0	675,0	0	0	711,0	1050,0	462,0	0

Lab Control pre-challenge	0	0	0	0	0	0	0	0	0
**Grade**	1	1	1	1	1	1	1	1	

Table 14. Eosinophilia in the paraclinical follow-up of immunized volunteers who presented an AE.

Urinalysis:
Proteinuria:
Proteinuria was found in 13 volunteers. Of these, 3 had proteinuria in paraclinical on the day of immunization.
Proteinuria was classified as Grade 1 (trace) in 4 volunteers (CS1006, CS1036, CS1037, CS1047); Grade 2 (+) in 5 volunteers (CS1001, CS1005, CS1012, CS1030, CS1038) and Grade 1 and 2 in 4 volunteers (CS1003, CS1015, CS1018, CS1031).
Of these, 8 volunteers belonged to the experimental group (CS1001, CS1006, CS1015, CS1030, CS1031, CS1036, CS1038, CS1547) and 5 to the control group (CS1003, CS1005, CS1012, CS1018, CS1037)

	**CS1001**	**CS1003**	**CS1005**	**CS1006**	**CS1012**	**CS1015**	**CS1018**	**CS1030**	**CS1031**	**CS1036**	**CS1037**	**CS1038**	**CS1547**
Selection	Neg	Neg	Neg	Neg	Neg	Neg	Neg	Neg	Neg	Neg	Neg	Neg	Neg
Lab Control Month 0	Neg	Neg	Neg	Neg	Neg	Pos	Pos	Pos		Neg	Neg	Neg	Neg
Lab Control Month 1	Pos	Neg	Pos	Pos	Pos	Pos	Neg	Neg	Pos	Neg	Neg	Neg	Neg
Lab Control Month 2	Neg	Pos	Neg	Neg	Neg	Neg	Neg	Neg	Neg	Pos	Pos	Neg	Pos
Lab Control Month 3	Neg	Neg		Neg	Neg	Neg	Pos	Neg	Neg	Neg	Neg	Neg	Neg
Lab Control Month 6	Neg	Pos		Neg	Neg	Neg	Neg	Neg	Pos	Neg	Neg	Pos	Neg

Table 15. Proteinuria in the paraclinical follow-up of immunized volunteers who presented an AE

Glycosuria:
He presented severe glycosuria (500mg / dL) in volunteer CS1584, subsequently diagnosed with diabetes mellitus (HbA1c: 8.1%).
Hematuria:

Hematuria was mild in 14 volunteers at 26 moments classified as Grade I. In two moments, it was classified as Grade II (Lab control month 0 in CS1569 and Lab control pre-challenge in CS1038).

	Selection	Lab Control Month 0	Lab Control Month 1	Lab Control Month 2	Lab Control Month 3	Lab Control Month 6	Lab Control pre-challenge	
Code	Erythrocytes	Erythrocytes	Erythrocytes	Erythrocytes	Erythrocytes	Erythrocytes	Erythrocytes	Classification EA
CS1005	0	-	-	5 - 8 xc	NA	NA	-	Grade I
CS1012	0-2xc	3-5xc	-	-	-	-	-	Grade I
CS1013	4-6xc	NA	5 xc	5 xc	1 xc	1 xc	eumorphs	Grade I
CS1023	*-	0-1xc	-	-	-	-	-	Grade I
CS1031	-		0-2 xc	-	-	-	-	Grade I
CS1037	0-2xc	0	3	5-8 xc	-	-	-	Grade I
CS1038	-	-	-	-	-	-	8-12xc	Grade I
CS1506	0-1xc	-	-	-	-	-	0 eumorphs	Grade I
CS1535	0-2xc	-	-	-	-	2 xc	2 xc	Grade I

CS1537	-	0-2 xc	5 xc	-	-	-	-	Grade I
CS1549	0	0	0	0	-	0	2 xc	Grade I
CS1565	0-2 xc	-	-	-	-	-	2xc	Grade I
CS1569	1-3xc	>25 xc eu, 1-3xc dis	-	-	-	5xc	0	Grade I-II
CS1575	2-4 xc	2 xc	5 xc	-	-	-	-	Grade I

Table 16. Hematuria in the paraclinical follow-up of immunized volunteers who presented an AE

Clinical manifestations after the Infectious Challenge (CHMI)

As expected, there were symptoms and signs of malaria infection.
Arthralgia: Of the 32 volunteers exposed to the infectious challenge, on eight occasions, they reported arthralgia in the face-to-face and telephone medical follow-ups after the infectious challenge. 75% of the time was related to the infectious challenge, and 12.5% of the time possibly and probably related.
Deterioration: On 16 occasions, the volunteers exposed to the infectious challenge reported deterioration, which was related in 43% (7/16) of the cases, possibly in 13%, and probably in 43% (7/16) infectious challenge.
Diaphoresis: On one occasion, a volunteer presented diaphoresis with hypotension (TA: 80/50), which responded to intravenous fluids.
Diarrhea: On five occasions, some volunteers had diarrhea which was found to be possibly related in 40% (2/5), in 40% (1/5) probably related, and 20% (1/5) probably unrelated to the infectious challenge.
Abdominal pain: On seven occasions, they had abdominal pain. Of these 28% (2/7) presented in the epigastrium and 72% (5/7) it was not specified, also in 28% (2/7) of the cases were considered probably related, in 14% (1/7) possibly related, in 28% (2/7), probably not related, in 14% (1/7) not related to the infectious challenge; in one case it was not specified.
Headache: Headache was reported on 48 occasions after the infectious challenge, of these: in 16% (8/48), it was considered related, in 29% (14/48), it was considered probably related, in the 20% (10/48) was considered possibly related, in 18% (9/48) it was considered probably unrelated, in 8% (4/48) it was deemed to be unrelated, there is no data for 6% (3 / 48).
Arm pain: In 85% (6/7), it was considered not related to the infectious challenge, and in 15% (1/7), it was considered related to the infectious challenge.
Chills: On 21 occasions, the volunteers had chills. In 76% (16/21), it was considered related, in 4% (1/21) probably related, in 4% (1/21) possibly related, and in the 14% (3/21) probably unrelated.
Fever: On 20 occasions, the volunteers presented fever after the infectious challenge. It was considered in 85% (17/20) related and in 15% probably unrelated (3/20).
Insomnia: On one occasion, insomnia occurred, which was considered possibly related to the infectious challenge.
General discomfort: On 41 occasions, the volunteers presented general discomfort. It was considered: in 39% (16/41) related, in 7% (3/41) probably related, in 7% (3/41) possibly related, in 20% (8/41) probably not related, in 2% (1/41) not related to the infectious challenge. However, in ten cases (24%), the general malaise was not related to the infectious challenge.
Myalgias: On 23 occasions, the volunteers presented myalgias. It was considered: in 61% (14/23) related, in 9% (2/23) probably related, in 17% (4/23) possibly related, probably 13% (3/23) unrelated to the CHMI.
Nausea: On 20 occasions the volunteers had nausea, it was considered: in 5% (1/20) related, in 50% (10/20) probably related, in 20% (4/20) possibly related, in 15% (3/20) probably unrelated, in 10% (2/20) unrelated.
Itching: On two occasions, itching was reported, which was not related to the infectious challenge or treatment.
Urticaria: On two occasions, the volunteers presented urticaria (20 minutes), which was

not related to the CHMI but rather to the xenodiagnosis. Urticaria lasting 4 min, unrelated to the CHMI, was also reported on one occasion.
Blurred vision: Blurred vision was reported on six occasions. It was considered: in 83% (5/6) possibly related, and in 17% (1/6) probably not related to the CHMI.
Others: 29 findings were reported as others which are recognized as alterations in the area of exposure to xenodiagnosis, the clinical picture of dyspnea and cough in treatment with salbutamol, viral vision, emesis, dizziness, myalgia of the lower limbs, dyspnea predominantly nocturnal, possible origin psychosomatic, cyst in the left ovary, rhinorrhea and earache, mild dizziness, dry cough, and vomiting.

Table 17. Clinical manifestations after the infectious challenge

Categorized Adverse Event	Description	Relationship to the Challenge	Relationship to Treatment	Total
**Arthralgia**	**NA**	Definitely related	Not related	5
			Probably unrelated	1
		Possibly related	Not related	1
		Probably related	Not related	1
	**Total NA**			**8**
**Total Arthralgia**				**8**
**Decay**	**NA**	Definitely related	Not related	7
		Possibly related	Not related	2
		Probably related	Not related	7
	**Total NA**			**16**
**Total Decay**				**16**
**Diaphoresis**	**Hypotension 80/50 responded to treatment with intravenous fluids**	NA	NA	1
	**Total, hypotension 80/50 responded to treatment with intravenous fluids**			**1**
	**NA**	Definitely related	Probably unrelated	1
	**Total NA**			**1**
**Total Diaphoresis**				**2**
**Diarrhea**	**NA**	Possibly related	Not related	1
			Possibly related	1

		Probably unrelated	Not related	1
		Probably related	Not related	1
			Possibly related	1
	**Total NA**			**5**
**Total Diarrhea**				**5**
**Abdominal pain**	**Abdominal pain in the epigastrium**	Probably unrelated	Not related	1
	**Total, Abdominal pain in the epigastrium**			**1**
	**epigastric pain**	Probably unrelated	Probably related	1
	**Total, epigastric pain**			**1**
	**NA**	NA	NA	1
		Not related	Possibly related	1
		Possibly related	Not related	1
		Probably related	Possibly related	1
			Probably related	1
	**Total NA**			**5**
**Total Abdominal pain**				**7**
**Headache**	**Global mild headache**	Probably unrelated	Probably related	1
	**Total Global mild headache**			**1**
	**Migraine-like headache**	Probably unrelated	Not related	1
	**Total Migraine-like headache**			**1**
	**NA**	Definitely related	Not related	6
			Possibly related	1

			Probably unrelated	1
		NA	NA	3
		Not related	Not related	2
			Possibly related	1
			Probably related	1
		Possibly related	Not related	9
		Probably unrelated	Not related	6
			Probably unrelated	1
		Probably related	Not related	14
	**Total NA**			**45**
	**Refers mild headache**	Possibly related	Not related	1
	**Total Refers mild headache**			**1**
**Total Headache**				**48**
**Arm pain**	**Pain in mosquito exposure area**	Not related	Not related	1
	**Total pain in mosquito exposure area**			**1**
	**Pain in the area of exposure to xenodiagnosis**	Not related	Not related	1
	**Total pain in the area of exposure to xenodiagnosis**			**1**
	**Pain in the area of exposure to xenodiagnosis**	Not related	Not related	1
	**Total pain in the area of exposure to xenodiagnosis**			**1**
	**Pain in the area of exposure to xenodiagnosis**	Not related	Not related	1

	**Total pain in the area of exposure to xenodiagnosis**			**1**
	**Pain in the area of exposure to xenodiagnosis**	Not related	Not related	1
	**Total pain in the area of exposure to xenodiagnosis**			**1**
	**NA**	Definitely related	Not related	1
	**Total NA**			**1**
	**Patient who had an accident with a polisher with lesion in the left hand thenar region**	Not related	Not related	1
	**Total Patient who had an accident with a polisher with lesion in the left hand thenar region**			**1**
**Total arm pain**				**7**
**Chills**	**NA**	Definitely related	Definitely related	1
			Not related	14
			Probably unrelated	1
		Possibly related	Not related	1
		Probably unrelated	Not related	3
		Probably related	Not related	1
	**Total NA**			**21**
**Total Chills**				**21**
**Fever**	**NA**	Definitely related	Not related	16
			Probably unrelated	1
		Probably unrelated	Not related	2
	**Total NA**			**19**
	**Unquantified fever**	Probably unrelated	Probably unrelated	1
	**Total Unquantified fever**			**1**

**Total Fever**				**20**
**Insomnia**	**NA**	Possibly related	Not related	1
	**Total NA**			**1**
**Total Insomnia**				**1**
**Malaise**	**Flu-like symptoms**	Not related	Not related	1
	**Total flu-like symptoms**			**1**
	**Viral infection**	Probably unrelated	Not related	1
	**Total viral infection**			**1**
	**Malaise, flu-like symptoms**	Possibly related	Not related	1
	**Total malaise, flu-like symptoms**			**1**
	**NA**	Definitely related	Not related	14
			Probably unrelated	2
		NA	NA	10
		Possibly related	Not related	1
		Probably unrelated	Not related	6
		Probably related	Not related	3
	**Total NA**			**36**
	**Malaise**	Probably unrelated	Probably unrelated	1
	**Total again after three days general malaise begins**			**1**
	**General malaise spontaneous resolution**	Possibly related	Not related	1
	**Total refers to general discomfort yesterday, spontaneously resolved**			**1**
**Total Malaise**				**41**
**Myalgia**	**NA**	Definitely related	Not related	13

			Probably unrelated	1
		Possibly related	Not related	4
		Probably unrelated	Not related	3
		Probably related	Not related	2
	**Total NA**			**23**
**Total Myalgia**				**23**
**Other**	**Alterations in the xenodiagnosis exposure area**	Not related	NA	1
	**Total Alterations in the xenodiagnosis exposure area**			**1**
	**A clinical case of more than one month of evolution consisting of dyspnea associated with dry cough, apparently without triggers. Treated with salbutamol inhaler.**	Probably unrelated	NA	1
	**Total, A clinical case of more than one month of evolution consisting of dyspnea associated with dry cough, apparently without triggers. Treated with salbutamol inhaler.**			**1**
	**Flu-like symptoms**	Not related	NA	1
	**Total, Flu-like symptoms**			**1**
	**Pain at mosquito exposure site**	Not related	NA	1
	**Total, pain at mosquito exposure site**			**1**
	**Vomiting**	Definitely related	NA	1

**Total, vomiting**			**1**
**With fever**	Probably unrelated	NA	1
**Total, with fever**			**1**
**Dizziness**	Probably unrelated	NA	1
**Total dizziness**			**1**
**Myalgia, only in lower limbs**	Probably unrelated	NA	1
**Total, myalgia, only in lower limbs**			**1**
**NA**	Not related	NA	7
	Probably unrelated	NA	2
**Total NA**			**9**
**Dyspnea, predominantly nocturnal in the sitting and ulnar position, is not exacerbated by exercise nor associated with respiratory symptoms. Possible psychosomatic origin is questioned, and it is decided to continue follow-up.**	Not related	NA	1
**Dyspnea, predominantly nocturnal in the sitting and ulnar position, is not exacerbated by exercise nor associated with respiratory symptoms. Possible psychosomatic origin is questioned, and it is decided to continue follow-up.**			**1**
**Flu-like symptoms**	Probably unrelated	NA	1
**Total Flu-like symptoms**			**1**

**Flu-like symptoms**	Probably unrelated	NA	1
**Total Flu-like symptoms**			**1**
**Probably of gynecological origin**	Not related	NA	1
**Total, Probably of gynecological origin**			**1**
**Cyst on the left side under follow-up by gynecology**	Not related	NA	1
**Cyst on the left side under follow-up by gynecology**			**1**
**Reports that dizziness is associated with the intake of primaquine.**	Probably unrelated	NA	1
**Total, Reports that dizziness is associated with the intake of primaquine.**			**1**
**Hyaline rhinorrhea / otalgia**	Not related	NA	1
**Total, Hyaline rhinorrhea /otalgia**			**1**
**Dizziness (feeling)**	Possibly related	NA	1
	Probably unrelated	NA	1
**Total, dizziness (feeling)**			**2**
**Mild dizziness (feeling)**	Probably unrelated	NA	1
**Total Mild dizziness (feeling)**			**1**
**Dry cough**	Probably unrelated	NA	1
**Total, dry cough**			**1**
**Vomiting**	Probably related	NA	1

	**Total, vomiting**			**1**
**Total NA**				**29**
**Nausea**	**NA**	Definitely related	Possibly related	1
		Not related	Not related	1
			Possibly related	1
		Possibly related	Not related	1
			Possibly related	1
			Probably unrelated	1
			Probably related	1
		Probably unrelated	Possibly related	1
			Probably related	1
		Probably related	Not related	7
			Probably related	3
	**Total NA**			**19**
	**Nausea accompanying epigastric pain**	Probably unrelated	Probably related	1
	**Total, Nausea accompanying epigastric pain**			**1**
**Total Nausea**				**20**
**Pruritus**	**1-week clinical picture of generalized itching, predominantly in the back.**	Not related	Not related	1
	**Total, 1-week clinical picture of generalized itching, predominantly in the back.**			**1**
	**Pruritus in the xenodiagnosis area**	Not related	Not related	1
	**Total, Pruritus in the xenodiagnosis area**			**1**

**Total Pruritus**				**2**
**Urticaria (20 minutes)**	**Pruritus in the xenodiagnosis area**	Not related	Not related	1
	**Total, Pruritus in the xenodiagnosis area**			**1**
	**Maculopapular rash at the mosquito exposure site**	NA	NA	1
	**Total, Maculopapular rash at mosquito exposure site**			**1**
**Total Urticaria (20 minutes)**				**2**
**Urticaria (4 minutes)**	**Pruritus in the exposure area**	NA	NA	1
	**Total, Pruritus in the exposure area**			**1**
**Total Urticaria (4 minutes)**				**1**
**Blurred vision**	**after taking antimalarials**	Probably unrelated	Possibly related	1
	**Total, after taking antimalarials**			**1**
	**NA**	Possibly related	Not related	3
			Possibly related	2
	**Total NA**			**5**
**Total blurred vision**				**6**
**Total, general**				**259**

Results of paraclinical in Infectious Challenge

The alterations in safety paraclinical during the CHMI were 27. They consisted of hyperglycemia 4% (1), the elevation of glutamic-pyruvic transaminase 19% (5), the elevation of indirect bilirubin without alteration of the AST / ALT pattern 4% (1), anemia 41 (11), leukopenia 11% (3), leukocytosis 11% (3), lymphopenia 4% (1), hematuria 7% (2). According to the FDA classification of AE, 85% of paraclinical AE during the infectious challenge correspond to Grade 1 and the remaining 15% to Grade 2. Safety paraclinical after the CHMI were taken on day 33 of the challenge.

Paraclinical results were registered in the RedCap database; the reference values were obtained from the Protocol. All recorded values were analyzed, and the paraclinical test alterations were classified according to the Protocol.

Glycemia: A fasting sample was taken. Therefore, the values were found in normal ranges. Volunteer CS1575 had blood glucose levels of 140 mg/dl, which would lead to a Grade I adverse event.

Creatinine and BUN: There was no elevation evidence to ranges considered an AE according to the protocol classification.

Transaminases: They presented Grade 1 elevation of Pyruvic Glutamic Transaminase, five volunteers of which 3 were women, and 2 were men. No elevation of Oxaloacetic Transaminases was evidenced in any of the volunteers after the challenge.

Volunteer	Value ALT (U/L)	Sex	Grade
CS1025	40	Woman	1
CS 1538	58	Woman	1
CS 1565	43	Woman	1
CS1015	54	Man	1
CS1575	74	Man	1

Table 18. Alteration in Glutamic Pyruvic and Oxaloacetic Transaminases

<u>**Direct bilirubin:**</u> No elevation was evidenced in post-challenge paraclinical.

Indirect bilirubin without alteration of the AST / ALT pattern: Only volunteer CS1506 presented Grade 1 elevation.

Paraclinical	Value
Bilirubin indirect	0.87
ALT	27
AST	29

Table 19. Indirect bilirubin alteration without compromise of the AST / ALT pattern

<u>Prothrombin Time- Partial Thromboplastin Time</u>: There were no alterations in clotting times

Blood count

On day 33, post-CHMI paraclinical tests indicated:

<u>Anemia</u>: 11 volunteers had mild anemia. According to the reference values of the protocol for the classification of the AE, seven volunteers (CS1005, CS1006, CS1554, CS1565, CS1570, CS1574, CS1581) had Grade 1 anemia (Hb 11-12 g / dL) and four volunteers

(CS1511, CS1537, CS1549, CS1569) Grade 2 (Hb 9.5-10.9 g / dL):

Volunteer	Hemoglobin	Grade
CS1005	11.8	1
CS1006	11.7	1
CS1511	10.6	2
CS1537	10.3	2
CS1549	10.8	2
CS1554	11.4	1
CS1565	11.2	1
CS1569	10.4	2
CS1570	11.6	1
CS1574	11.8	1
CS1581	11.0	1

Table 20. Alteration in Hb values

Leukopenia: Grade 1 leukopenia was evidenced in three volunteers (CS1006, CS1028, CS1037).

Volunteer	Leucocytes /ul	Grade
CS1006	2800	1
CS1028	3000	1
CS1037	3000	1

Table 21. Leukopenia post-CHMI paraclinical follow-up.

Leukocytosis: Grade 1 leukocytosis was evidenced in three volunteers (CS1012, CS1547, CS1570).

Volunteer	Leucocytes /ul	Grade
CS1012	11.5	1
CS1570	11.2	1
CS1547	11.2	1

Table 22. Leukocytosis in post-CHMI paraclinical follow-up

Lymphopenia: In post-challenge paraclinical, only volunteer CS1006 had Grade 1 lymphopenia (896).
Thrombocytopenia: Thrombocytopenia was not evidenced.

Urine test

Glycosuria: in the RedCap report, there is no evidence of altered glucose values in urine.
Proteinuria: no proteinuria was evidenced in any of the volunteers post-CHMI.
Hematuria: Volunteer CS1013 and volunteer CS1553 presented Grade 1 hematuria.

	**Selection**	**Lab Control Month 0**	**Lab Control Month 1**	**Lab Control Month 2**	**Lab Control Month 3**	**Lab Control Month 6**	**Lab Control pre-challenge**	**Challenge**
**Code**	**Erythrocytes:**	**Erythrocytes:**	**Erythrocytes:**	**Erythrocytes:**	**Erythrocytes:**	**Erythrocytes:**	**Erythrocytes:**	**Erythrocytes:**
CS1013	4-6xc	-	5.00	5	1	1	eumorphs	5 eumorphs
CS1553	-	-	-	-	-	-	-	1 eumorphs

Table 23. Hematuria in post-challenge paraclinical follow-up

However, volunteer CS1013 from the selection paraclinical presented Grade I hematuria. There was likely no relationship between the hematuria post-CHMI and the investigation (immunizations and CHMI). Volunteer CS1553 presented Grade I hematuria on the lower levels.

Supplements

Supplementary methods

Enzyme-linked immunosorbent assays (ELISA)

Peptides specific antibodies against *Pv*CS N, R, or C fragments (#2304-49326202, batch P0RGB, Pharmacie, Centre Hospitalier Universitaire Vaudois, Lausanne, Switzerland) were assessed by ELISA as described elsewhere[18]. Briefly, microplates (Nunc-Immuno Plate, Maxisorp, Roskilde, Denmark) were coated overnight at 4º C with CS peptides at a concentration of 1 mg/ml in PBS (# P4417-100TAB, Sigma Chemical Co., St Louis, MO, USA). Plates were then blocked with 5% skim milk (Difco ™, Skim Milk, ref: 232100) in PBS pH 7.4 for 2 h at room temperature. After washing, serial dilution of the samples was carried out in PBS/2.5% skim-milk/0.05% Tween-20 (#BP337-500, CAS9005-64-5, Fisher Scientific Co L.L.C., USA), and incubated in duplicate for 1 h at room temperature. IgG antibodies were detected using phosphatase-conjugated goat anti-human IgG immunoglobulin (Fc specific, Alkaline Phosphatase antibody, #A9544, batch 019M4818V, Sigma-Aldrich, Co, St Louis, MO, USA) at a dilution of 1:1000. The enzymatic activity was developed after incubation for 45 min at room temperature with para-nitrophenyl phosphate substrate. Absorbance was measured at 450 nm in a Microplate Reader (Dinex Technologies, Inc., MRX Chantilly, VA).

IFN-γ-ELIspot production

The number of IFN-γ producing PBMC was determined using human IFN-γ ELIspot reagents from MABTECH AB, Stockholm, Sweden as previously described [17]. Microtitre plate wells (Millipore, MAHA S45, Bedford, MA, USA) were coated with 5 mg/ml of anti-human IFN-γ mAb (1-D1K, code 3420-3-1000, batch 64.1, MABTECH AB, Sweden) overnight at 4ºC. After blocking the plates with RPMI (RPMI 1640, Ref:6404, Sigma-Aldrich, Co, St Louis, MO, USA), medium plus 10% fetal calf serum (FCS, Ref: F0926, Merck KGaA, Darmstadt, Germany) for 2 h at room temperature, a suspension of $2x10^5$ fresh PBMC/well was mixed with the synthetic peptides at a concentration of 10 mg/ml. Plates were incubated for 40 h at 37ºC in a 5% CO2 in an atmosphere of 95% air. After washing with PBS–Tween-20 (PBS-T) 0.05%, a biotinylated anti-IFN-γ mAb (7-B6-1, Code 3420-6-1000, batch 33.1MABTECH AB, Sweden) at 1 mg/ml was added and

incubated overnight at 4ºC. Streptavidine-alkaline phosphatase (code 330310-10, batch 40318-10, MABTECH AB, Sweden) diluted 1:1000 was added and the reaction developed with the substrate BCIP/NBT (5-bromo-2-chloro-3-indolyl phosphatase/ Nitroblue Tetrazolium) (Sigma, St Louis, MO, USA) leading to the appearance of dark blue spots. The number of spots was determined using a stereomicroscope by two independent readers at a 40x magnification. Results are expressed as the mean number of IFN-γ spot-forming cells (SFCs) per 10^6 PBMC.

Indirect immunofluorescence / IFAT.

Plasmodium vivax (VK210) sporozoites fixed slides produced by experimental infection of *An. Albimanus* mosquitoes were used [21]. Briefly, sporozoites fixed to multisport microscope slides with PBS containing 2% bovine serum albumin (BSA) were incubated in a humid chamber in the dark for 1 hour at 37°C, with 25 µl of serum diluted in PBS-BSA 2%, starting with a 1:20 dilution. After three washes in PBS, a FITC-labeled anti-human IgG (Fluorecein conjugate affinity pure goat anti-human IgG (H+L, catalogue number 109-095-003, Lot140577, Jackson Immuno Reseach Laboratories, Inc, Baltimore, USA) was added at 1: 200 dilution in PBS-Evans Blue 0.05%. Slides were examined under an epifluorescence microscope (Nikon Elipse 50i.LH-M100C-1 03908, Nikon, Japan). Control positive was a pool *P. vivax* serum from infected donors and the negative controls was a pool of naïve donors. Antibody titers were determined as the reciprocal endpoint dilution that showed positive fluorescence. Titers >1:20 were considered as positive
