# Supplementary material for: Randomized clinical trial to assess the protective efficacy of a Plasmodium vivax CS synthetic vaccine
Source: Nat Commun. 2022 Mar 25;13:1603. doi: 10.1038/s41467-022-29226-3 (PMC8956637; doi:10.1038/s41467-022-29226-3)
Supplement: Supplementary file 3 — Reporting Summary [file 41467_2022_29226_MOESM3_ESM.pdf]

nature research

Corresponding author(s): Sócrates Herrera
Last updated by author(s): 2022/02/03

Reporting Summary

Nature Research wishes to improve the reproducibility of the work that we publish. This form provides structure for consistency and transparency in reporting. For further information on Nature Research policies, see our Editorial Policies and the Editorial Policy Checklist.

Statistics

For all statistical analyses, confirm that the following items are present in the figure legend, table legend, main text, or Methods section.

n/a	Confirmed	
☐	☒	The exact sample size (*n*) for each experimental group/condition, given as a discrete number and unit of measurement
☐	☒	A statement on whether measurements were taken from distinct samples or whether the same sample was measured repeatedly
☐	☒	The statistical test(s) used AND whether they are one- or two-sided<br>*Only common tests should be described solely by name; describe more complex techniques in the Methods section.*
☐	☒	A description of all covariates tested
☐	☒	A description of any assumptions or corrections, such as tests of normality and adjustment for multiple comparisons
☐	☒	A full description of the statistical parameters including central tendency (e.g. means) or other basic estimates (e.g. regression coefficient) AND variation (e.g. standard deviation) or associated estimates of uncertainty (e.g. confidence intervals)
☐	☒	For null hypothesis testing, the test statistic (e.g. *F*, *t*, *r*) with confidence intervals, effect sizes, degrees of freedom and *P* value noted<br>*Give P values as exact values whenever suitable.*
☒	☐	For Bayesian analysis, information on the choice of priors and Markov chain Monte Carlo settings
☐	☒	For hierarchical and complex designs, identification of the appropriate level for tests and full reporting of outcomes
☒	☐	Estimates of effect sizes (e.g. Cohen's *d*, Pearson's *r*), indicating how they were calculated

Our web collection on statistics for biologists contains articles on many of the points above.

Software and code

Policy information about availability of computer code

Data collection	Data were collected in REDCap version 5.10.1(Nashville, TN, USA) electronic data capture
Data analysis	Data analyzed was using SPSS version 16.0 software (SPSS Inc., Chicago, IL, USA) and plotted using Graph Pad Prism version 6.0 (GraphPad Software, San Diego, California, USA).

For manuscripts utilizing custom algorithms or software that are central to the research but not yet described in published literature, software must be made available to editors and reviewers. We strongly encourage code deposition in a community repository (e.g. GitHub). See the Nature Research guidelines for submitting code & software for further information.

Data

Policy information about availability of data

All manuscripts must include a data availability statement. This statement should provide the following information, where applicable:

- Accession codes, unique identifiers, or web links for publicly available datasets
- A list of figures that have associated raw data
- A description of any restrictions on data availability

The author confirms that the data supporting the findings of this study are available within the article and its Supplementary Material. Raw data that support the findings are in the REDCap database, available from the corresponding author upon reasonable request; S.H (sherrera@inmuno.org). The timeframe for responding to the requests will be approximately 25 business days. The data are not publicly available because they contain information compromising research participants' privacy/consent. This trial is registered on ClinicalTrials.gov under the identifier NCT02083068.

Field-specific reporting

Please select the one below that is the best fit for your research. If you are not sure, read the appropriate sections before making your selection.

- [x] Life sciences
- [ ] Behavioural & social sciences
- [ ] Ecological, evolutionary & environmental sciences

For a reference copy of the document with all sections, see nature.com/documents/nr-reporting-summary-flat.pdf

Life sciences study design

All studies must disclose on these points even when the disclosure is negative.

Sample size	The study sample size was calculated with a confidence level of 95%, taking into account a previous census of ~5,603 subjects with recent malaria infections in Buenaventura, 36 where the estimated prevalence of the Fy+ genotype (30%) and G6PD deficiency (12%) in the target population indicated that only 1,479 subjects would be suitable for participation. Likewise, we estimated that only 3-10% of the subjects (44-147) would volunteer for the trial based on previous studies in the same area. Citation 36: Vallejo, A. F. et al. High prevalence of sub-microscopic infections in Colombia. Malar J 14, 201, doi:10.1186/s12936-015-0711-6 (2015).
Data exclusions	As exclusion criteria, Volunteers were excluded if they had diseases or medical conditions that would alter the vaccine's assessment or any condition that could increase the risk of adverse outcomes. For the subject's selection and enrollment, naïve volunteer's group were excluded from enrolment for the, 1) malaria history of having lived in an endemic area for the past 6 months, and 2) excluded from the semi-immune group if they had negative IFAT (< 1:20) for P. vivax. In addition, Potential parasite donors were excluded from enrolment if 1) they had negative IFAT (< 1:20) for P. vivax in screening tests, 2) if patients had chronic or acute disease, different from malaria by P. vivax; 3) hemoglobin levels <9 g/dL at the time of recruitment, 4) having received antimalarial treatment before blood draw; 5) having a history of disease or clinical conditions that according to medical criteria might increase significantly the risk related with participation on the study.<br><br>For this trial from 121 volunteers who initially accepted screening (38 naïve, 83 semi-immune), 86 were excluded or declined participation and 35 were enrolled. Seventeen were naïve and were allocated to Phase IIa study [12 Experimental (Exp) + 5 Control (Ctrl)], and 18 semi-immune to the Phase IIb study (13 Exp + 5 Ctrl). All 35 volunteers (age range 19-44 years) were immunized with PvCS LSP or placebo formulated in Montanide ISA-51. Two volunteers withdrew after the first immunization, and one more (semi-immune) was dropped out because of diabetes mellitus diagnosis.
Replication	All assays were performed in duplicate to verify the reproducibility of the experimental findings. All attempts at replication were successful.
Randomization	Participants were randomly (simple) assigned in a 2:1 ratio. A blinded data manager controlled the allocation to receive the vaccine (Experimental; Exp, n=25) or placebo (Control; Ctrl, n=10) (Figure 1). The naïve group was further divided into Exp (n=12) and Ctrl (n=5) and the semi-immune group into Exp (n=13) and Ctrl (n=5).
Blinding	Access to the randomization code was strictly controlled at the pharmacyn to guarantee concealment sequence.

Reporting for specific materials, systems and methods

We require information from authors about some types of materials, experimental systems and methods used in many studies. Here, indicate whether each material, system or method listed is relevant to your study. If you are not sure if a list item applies to your research, read the appropriate section before selecting a response.

Materials & experimental systems

n/a	Involved in the study	
[ ]	[x]	Antibodies
[x]	[ ]	Eukaryotic cell lines
[x]	[ ]	Palaeontology and archaeology
[ ]	[x]	Animals and other organisms
[ ]	[x]	Human research participants
[ ]	[x]	Clinical data
[x]	[ ]	Dual use research of concern

Methods

n/a	Involved in the study	
[x]	[ ]	ChIP-seq
[x]	[ ]	Flow cytometry
[x]	[ ]	MRI-based neuroimaging

Antibodies

Antibodies used	Phosphatase-conjugated goat anti-human IgG immunoglobulin (Fc specif.ic, Alkaline Phosphatase antibody, #A9544, batch 019M4818V, Sigma-Aldrich, Co, St Louis, MO, USA).<br>- Anti-human IFN-g mAb (1-D1K, code 3420-3-1000, batch 64.1, MABTECH AB, Sweden).<br>- Biotinylated anti-IFN-g mAb (7-B6-1, Code 3420-6-1000, batch 33.1MABTECH AB, Sweden).

- FITC-labeled anti-human IgG (Fluorescein conjugate affinity pure goat anti-human IgG (H+L, catalogue number 109-095-003, Lot140577, Jackson Immuno Research Laboratories, Inc, Baltimore, USA)

Validation	Only commercially available secondary antibodies were used.

Animals and other organisms

Policy information about studies involving animals; ARRIVE guidelines recommended for reporting animal research

Laboratory animals	Female, Anopheles Albuminous mosquitoes, 3-4 days after egg emerging.
Wild animals	Study did not involve wild animals.
Field-collected samples	No field collected samples were used in the study
Ethics oversight	The study protocol was reviewed and approved by the Institutional Review Board of Malaria Vaccine and Drug Development Center (CECIV-MVDC) and Centro Medico Imbanaco (CMI# 0992304-493-26202)

Note that full information on the approval of the study protocol must also be provided in the manuscript.

Human research participants

Policy information about studies involving human research participants

Population characteristics	Naïve volunteers were from Cali, (Capital of Valle del Cauca department), a malaria-free area, located at the southwest of Colombia at 1.000 m.a.s.l. Volunteer were e based on no history of malaria and negative P. vivax serology. The semi-immune volunteers were recruited in Buenaventura, a main port on the Pacific coast of Colombia, at 80 km from Cali with 7 m.a.s.l. of altitude. The region is a low to moderate malaria-endemic area were both P. vivax and P. falciparum parasites are transmitted.
Recruitment	Participants were recruited from 03 October 2014 (first patient in) until 22 December 2014 (Last patient in). Invitation to participate was based on a available census of 5603 patients (database) . No potential biases were identified.
Ethics oversight	The study protocol was reviewed and approved by the Institutional Review Boards of the Malaria Vaccine and Drug Development Center (CECIV-MVDC) and Centro Médico Imbanaco (CMI # 0992304-493-26202).

Note that full information on the approval of the study protocol must also be provided in the manuscript.

Clinical data

Policy information about clinical studies

All manuscripts should comply with the ICMJEguidelines for publication of clinical research and a completedCONSORT checklist must be included with all submissions.

Clinical trial registration	NCT02083068
Study protocol	Supplementary information
Data collection	Naïve volunteers were from Cali (Colombia), a non-malaria endemic city, with eligibility based on no history of malaria and negative serology against P. vivax blood stages. Semi-immune volunteers were recruited from Buenaventura, a low to moderate malaria-endemic area of Colombia. Participants were recruited from 03 October 2014 (first patient in) until 22 December 2014 (Last patient in).
Outcomes	The primary outcome was the P. vivax CS LSO vaccines' protective efficacy against the P.vivax CHMI in malaria-naïve and semi-immune volunteers as determined by the parasite reduction in terms of frequency and density and the secondary outcome, the B and T-cell immune response associated with protection. B cell (specific antibodies) response was determined by ELISA and T-cell response was determined by ELIspot to measure production on INF- upon stimulation with the vaccine fragments
